# A first-in-class leucyl-tRNA synthetase inhibitor, ganfeborole, for rifampicin-susceptible tuberculosis: a phase 2a open-label, randomized trial

**DOI:** 10.1038/s41591-024-02829-7

**Published:** 2024-02-16

**Authors:** Andreas H. Diacon, Clifton E. Barry, Alex Carlton, Ray Y. Chen, Matt Davies, Veronique de Jager, Kim Fletcher, Gavin C. K. W. Koh, Irina Kontsevaya, Jan Heyckendorf, Christoph Lange, Maja Reimann, Sophie L. Penman, Rhona Scott, Gareth Maher-Edwards, Simon Tiberi, Georgios Vlasakakis, Caryn M. Upton, David Barros Aguirre

**Affiliations:** 1 TASK, Cape Town, South Africa; 2 National Institutes of Health, Bethesda, MD USA; 3 Institute of Infectious Disease and Molecular Medicine, University of Cape Town, Cape Town, South Africa; 4 GSK, London, UK; 5 Research Center Borstel, Leibniz Lung Center, Borstel, Germany; 6 German Center for Infection Research, Borstel, Germany; 7 Respiratory Medicine and Infectious Diseases, University of Lübeck, Lübeck, Germany; 8 Research Center Borstel, Leibniz Lung Center, German Center for Infection Research, Borstel and the University of Lübeck, Lübeck, Germany; 9 Department of Internal Medicine I, University Medical Center Schleswig-Holstein, Kiel, Germany; 10 Baylor College of Medicine and Texas Children’s Hospital, Houston, TX USA; 11 Blizard Institute, Barts and The London School of Medicine and Dentistry, Queen Mary University of London, London, UK; 12 GSK, Philadelphia, PA USA; 13 GSK, Tres Cantos, Spain; 14 Present Address: Generate:Biomedicines, Boston, MA USA; 15 Present Address: Imperial College London, London, UK

**Keywords:** Tuberculosis, Drug development

## Abstract

New tuberculosis treatments are needed to address drug resistance, lengthy treatment duration and adverse reactions of available agents. GSK3036656 (ganfeborole) is a first-in-class benzoxaborole inhibiting the *Mycobacterium tuberculosis* leucyl-tRNA synthetase. Here, in this phase 2a, single-center, open-label, randomized trial, we assessed early bactericidal activity (primary objective) and safety and pharmacokinetics (secondary objectives) of ganfeborole in participants with untreated, rifampicin-susceptible pulmonary tuberculosis. Overall, 75 males were treated with ganfeborole (1/5/15/30 mg) or standard of care (Rifafour e-275 or generic alternative) once daily for 14 days. We observed numerical reductions in daily sputum-derived colony-forming units from baseline in participants receiving 5, 15 and 30 mg once daily but not those receiving 1 mg ganfeborole. Adverse event rates were comparable across groups; all events were grade 1 or 2. In a participant subset, post hoc exploratory computational analysis of ^18^F-fluorodeoxyglucose positron emission tomography/computed tomography findings showed measurable treatment responses across several lesion types in those receiving ganfeborole 30 mg at day 14. Analysis of whole-blood transcriptional treatment response to ganfeborole 30 mg at day 14 revealed a strong association with neutrophil-dominated transcriptional modules. The demonstrated bactericidal activity and acceptable safety profile suggest that ganfeborole is a potential candidate for combination treatment of pulmonary tuberculosis.

ClinicalTrials.gov identifier: NCT03557281.

## Abstract (web summary)

A new inhibitor of *Mycobacterium tuberculosis* leucyl-tRNA synthetase is associated with bactericidal activity in humans and may be a candidate for combination therapy of tuberculosis.

## Main

In 2021, tuberculosis (TB) was the thirteenth leading cause of death, with 1.6 million deaths worldwide, and the second highest cause of death due to an infectious disease, briefly surpassed only by coronavirus disease 2019 (refs. ^1,2^). Without adequate treatment, approximately 70% of patients with active TB will die^3^. Despite international efforts, the global incidence of TB has not declined substantially over the past decade, and the rise of drug-resistant strains of *Mycobacterium tuberculosis* is severely hindering the World Health Organization’s end TB strategy^2,4^.

The current standard of care (SOC) for drug-susceptible TB consists of a 6-month course of four first-line antimicrobial drugs: rifampicin, isoniazid, pyrazinamide and ethambutol^5^. However, resistance to available anti-TB agents has emerged, and the overall treatment success of patients with multidrug-resistant TB (MDR-TB; resistance to at least isoniazid and rifampicin) is 60% (ref. ^6^). MDR-TB is related to 15–20% of antimicrobial resistance-attributed deaths globally^7^, despite regulatory approval of three new agents in the past 10 years. Bedaquiline^8^, delamanid^9^ and pretomanid^10^ are licensed for the treatment of drug-resistant TB; however, their optimal dose, use in combination treatments and potential for treatment shortening are still being explored. Furthermore, many second-line drugs are more costly, and less tolerable and effective than the first-line treatment options^11^, and their high pill burden, lengthy treatment duration and toxicity can impact patient compliance^11^. The emergence of *M. tuberculosis* strains resistant to all available TB drugs in the past two decades highlights the clinical need for further treatment options to combat TB^12–16^. Additional drugs and investigational treatments are currently in development to address drug resistance, lengthy treatment duration and adverse drug reactions of available agents^17^.

GSK3036656 (ganfeborole) is a novel 3-aminomethyl 4-chloro benzoxaborole, selectively inhibiting the *M. tuberculosis* leucyl-tRNA synthetase^18,19^. Ganfeborole has displayed promising activity in vitro against laboratory strains of *M. tuberculosis*, including drug-susceptible TB, MDR-TB and extensively drug-resistant TB (MDR plus resistance to at least a fluoroquinolone and either linezolid and/or bedaquiline^20^) clinical isolates^19^. In mice, ganfeborole showed 100% bioavailability, excellent exposure and low clearance, and antitubercular activity^19^. Furthermore, in vivo assessments in a marmoset monkey model of *M. tuberculosis* infection demonstrated that ganfeborole at 2 mg/kg/day and 0.5 mg/kg/day per day for 8 weeks led to a mean decrease in bacterial burden by 2.7 and 2.6 log_10_colony-forming units (CFUs)/lung lesion, respectively^21^. A phase 1 study assessed the safety, tolerability and pharmacokinetics (PK) following single (5, 15 and 25 mg once daily (QD)) and repeat (5 and 15 mg QD for 14 days) doses of ganfeborole in healthy participants^22^. The compound was well tolerated, and no relevant adverse events (AEs) were noted. A population PK analysis together with a translational PK/pharmacodynamics (PK/PD) model^23^ were then used to inform the clinical doses in this subsequent phase 2a study in participants with a predicted early bactericidal activity (EBA) in clinical doses ranging from 5 to 25 mg QD.

In this article, a phase 2a randomized study, the aim was to assess the EBA, safety, and PK of ganfeborole monotherapy at a range of dosages in male participants with a new episode of untreated, rifampicin-susceptible pulmonary TB. In addition, a post hoc exploratory analysis was undertaken to assess changes in positron emission tomography/computed tomography (PET/CT) signals and transcriptional responses. See Extended Data Table 1 for a plain language summary of this study.

## Results

### Participant disposition and characteristics

**Fig. 1:**
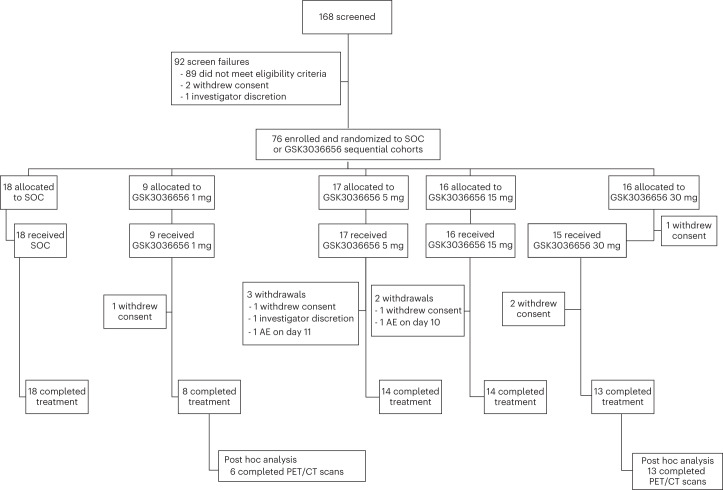
Participant disposition. CONSORT diagram of study enrollment, allocation to groups, treatment and treatment withdrawals. This study utilized a sequential dose cohort design. AE, adverse event; PET/CT, positron emission tomography/computed tomography; SOC, standard of care.

Of the 168 male participants with newly diagnosed, rifampicin-susceptible pulmonary TB screened, 76 (45%) were enrolled and randomized to either control (SOC; weight-based combination treatment with rifampicin, isoniazid, pyrazinamide and ethambutol) or sequential dose cohorts of ganfeborole 1, 5, 15 and 30 mg QD for 14 days (with a loading dose of 5, 15, 30 and 75 mg on day 1, respectively). Note that initial cohorts were 5, 15 and 30 mg, and the 1 mg group was subsequently added to help define the dose response curve (see Methods for further explanation). The study was not designed or powered to compare across study cohorts and within cohort comparisons are made to baseline. Overall, 100% (*n* = 18), 89% (*n* = 8), 82% (*n* = 14), 88% (*n* = 14) and 87% (*n* = 13) of treated participants completed the study treatment for SOC, ganfeborole 1, 5, 15 and 30 mg, respectively (Fig. 1).

The participants were categorized into the following populations:

- Safety population: all randomized participants who received at least one dose of the study treatment.
- Efficacy population: all the participants in the safety population who provided at least two evaluable overnight sputum samples (one of which had to be a baseline sample).
- PK population: all the participants in the safety population who received at least one dose of ganfeborole and had at least one evaluable PK sample.

All demographic characteristics, including age and body mass index (BMI), were similar across groups (Table 1). All participants were Black, and most smoked <20 cigarettes per day.

**Table 1:** Demographic and baseline characteristics (safety population)

	SOC	GSK3036656 (ganfeborole)			
Characteristic	*N* = 18	1 mg	5 mg	15 mg	30 mg
		*N* = 9	*N* = 17	*N* = 16	*N* = 15
Male sex, *n* (%)	18 (100)	9 (100)	17 (100)	16 (100)	15 (100)
Age (years), mean (s.d.)	37.0 (10.20)	37.8 (8.38)	38.1 (10.33)	39.8 (12.33)	38.5 (9.82)
Race, *n* (%)					
Black	18 (100)	9 (100)	17 (100)	16 (100)	15 (100)
Height (cm), mean (s.d.)	172.9 (4.85)	169.6 (4.07)	169.9 (7.71)	172.8 (5.81)	170.7 (5.60)
Weight (kg), mean (s.d.)	55.22 (6.410)	51.36 (4.085)	53.24 (6.235)	58.49 (10.457)	53.21 (6.376)
BMI (kg/m²), mean (s.d.)	18.46 (1.861)	17.87 (1.399)	18.44 (1.727)	19.55 (2.956)	18.27 (2.099)
Cigarettes per day	16	9	15	14	12
*n* (%)					
<20	15 (94)	7 (78)	13 (87)	10 (71)	12 (100)
≥20	1 (6)	2 (22)	2 (13)	4 (29)	0
HIV positive, *n* (%)	0	0	0	2 (13)	0
Baseline CFU (log_10_CFU) (efficacy population)					
*n*	17	9	17	16	14
Mean (s.d.)	6.28 (0.736)	6.30 (1.485)	5.95 (0.949)	5.94 (0.810)	5.97 (1.498)
Baseline TTP (h) (efficacy population)					
Mean (s.d.)	107.74 (34.979)	120.03 (53.002)	112.28 (27.885)	95.63 (21.615)	119.85 (42.229)

BMI, body mass index; CFU, colony-forming units; HIV, human immunodeficiency virus; SD, standard deviation; SOC, standard of care; TTP, time to sputum culture positivity.

### Early bactericidal activity

Assessing the EBA (the measure of efficacy used in this study) of anti-TB drugs by determining the viable sputum load of *M. tuberculosis* over the first 14 days of treatment is an established method for the early clinical evaluation of new agents^24,25^. Drug activity is determined by the reduction in log_10_CFU or the increase of time to sputum culture positivity (TTP) over time, which correlates well with log_10_CFU^26^. A control group of SOC is included in such studies to determine the reproducibility of the microbiological assays; this was an open-label trial and was not designed to directly compare the EBA of SOC with ganfeborole.

**Fig. 2:**
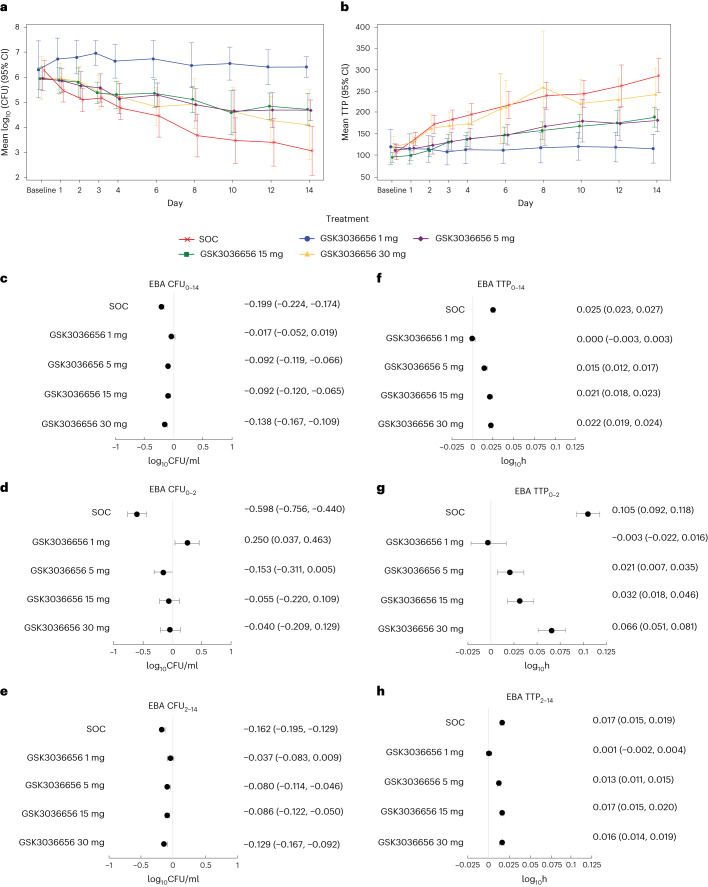
EBA over time during the study and rate of change per day in EBA over the periods baseline to day 14, baseline to day 2, and day 2 to day 14 (mixed model repeated measures analysis). **a**, log_10_CFU over time for the different treatment groups. **b**, TTP (in h) over time for the different treatment groups. Data are mean estimate (95% CI). Efficacy population (SOC, *N* = 18; GSK3036656 (ganfeborole) 1 mg, *N* = 9; GSK3036656 (ganfeborole) 5 mg, *N* = 17; GSK3036656 (ganfeborole) 15 mg, *N* = 16; GSK3036656 (ganfeborole) 30 mg, *N* = 15). **c**, EBA CFU_0–14_. **d**, EBA CFU_0–2_. **e**, EBA CFU_2–14_. **f**, EBA TTP_0–14_. **g**, EBA TTP_0–2_. **h**, EBA TTP_2–14_. Data are mean estimate (95% CI). Efficacy population (SOC, *N* = 18; GSK3036656 (ganfeborole) 1 mg, *N* = 9; GSK3036656 (ganfeborole) 5 mg, *N* = 17; GSK3036656 (ganfeborole) 15 mg, *N* = 16; GSK3036656 (ganfeborole) 30 mg, *N* = 15). EBA CFU_0–14_, EBA CFU_0–2_ and EBA CFU_2–14_, log_10_CFU/ml sputum samples over the periods baseline to day 14, baseline to day 2, and day 2 to day 14, respectively. EBA TTP_0–14_, EBA TTP_0–2_ and EBA TTP_2–14_, TTP over the periods baseline to day 14, baseline to day 2, and day 2 to day 14, respectively. CFU, colony-forming units; CI, confidence interval; EBA, early bactericidal activity; SOC, standard of care; TTP, time to culture sputum positivity.

Participants receiving ganfeborole 5–30 mg and SOC displayed a decrease in log_10_CFU/ml sputum (Fig. 2a) and an increase in TTP (Fig. 2b) over time, indicating positive responses for both endpoints. Log_10_CFU/ml sputum, the primary endpoint, decreased numerically from baseline to day 14 (EBA CFU_0–14_) for the ganfeborole 5–30 mg and SOC groups (Fig. 2c). The 1 mg group led to the smallest decline in bacterial load. A bilinear trend was not found for the ganfeborole groups, which is consistent with the log_10_CFU graph (Fig. 2a), in which the decrease appears linear.

Over the periods baseline to day 2 and day 2 to day 14 (secondary endpoints EBA CFU_0–2_ and EBA CFU_2–14_, respectively) CFU counts also decreased for the ganfeborole 5–30 mg and SOC groups (Fig. 2d,e). Similarly, TTP for all endpoints (EBA TTP_0–14_, EBA TTP_0–2_ and EBA TTP_2–14_) increased for participants receiving ganfeborole 5–30 mg and SOC (Fig. 2f–h).

### Safety

AE rates were comparable across groups (Table 2). All AEs reported during this study were either grade 1 or grade 2. No serious AEs (SAEs) were reported. One participant in the ganfeborole 15 mg group was withdrawn on day 10 due to suspected disease progression, and one participant in the ganfeborole 5 mg group was withdrawn on day 11 due to increasing hemoptysis (both discontinuations were considered related to TB progression by the investigator). The most common AEs across all ganfeborole groups were hemoptysis (overall *n* = 8) and pruritus (overall *n* = 7) (Table 2). The most common AEs in the SOC group were abdominal pain (*n* = 3) and headache (*n* = 3). Dyspepsia, vomiting, neutropenia and eye irritation were reported exclusively in the ganfeborole groups (Table 2). There was no increase in drug-related AEs with increasing ganfeborole dose.

**Table 2:** Summary of AEs and AEs experienced by more than one participant across any treatment group (safety population)

	SOC	GSK3036656 (ganfeborole)			
Event	*N* = 18	1 mg	5 mg	15 mg	30 mg
		*N* = 9	*N* = 17	*N* = 16	*N* = 15
Any AE, *n* (%)	14 (78)	7 (78)	14 (82)	15 (94)	9 (60)
Grade 1	10 (56)	6 (67)	11 (65)	12 (75)	9 (60)
Grade 2	4 (22)	1 (11)	3 (18)	3 (19)	0
Grade 3	0	0	0	0	0
SAE, *n* (%)	0	0	0	0	0
AE leading to withdrawal from study treatment, *n* (%)	0	0	1 (6)	1 (6)	0
AE leading to withdrawal from study, *n* (%)	0	0	0	0	0
Drug-related AE, *n* (%)	5 (28)	4 (44)	4 (24)	5 (31)	2 (13)
AEs experienced by more than one participant across any treatment groups					
Gastrointestinal disorders					
Abdominal pain	3 (17)	0	0	0	2 (13)
Nausea	2 (11)	0	0	2 (13)	0
Diarrhea	1 (6)	0	2 (12)	0	0
Dyspepsia	0	1 (11)	1 (6)	1 (6)	1 (7)
Vomiting	0	0	1 (6)	1 (6)	2 (13)
Nervous system disorders					
Headache	3 (17)	2 (22)	1 (6)	2 (13)	1 (7)
Dizziness	2 (11)	1 (11)	1 (6)	1 (6)	0
Skin and subcutaneous tissue disorders					
Pruritus	2 (11)	2 (22)	1 (6)	4 (25)	0
Rash pruritic	2 (11)	1 (11)	0	0	1 (7)
Rash papular	1 (6)	0	0	2 (13)	0
Skin hypopigmentation	1 (6)	0	0	0	1 (7)
Musculoskeletal and connective tissue disorders					
Arthralgia	2 (11)	0	1 (6)	4 (25)	0
Myalgia	1 (6)	1 (11)	0	2 (13)	0
Respiratory, thoracic and mediastinal disorders					
Hemoptysis	2 (11)	0	5 (29)	2 (13)	1 (7)
Pleuritic pain	1 (6)	0	2 (12)	1 (6)	0
Blood and lymphatic system disorders					
Neutropenia	0	0	2 (12)	0	0
Eye disorders					
Eye irritation	0	0	0	1 (6)	1 (7)

AE, adverse event; SAE, serious adverse event; SOC, standard of care.

### Pharmacokinetics

**Extended Data Fig. 1:**
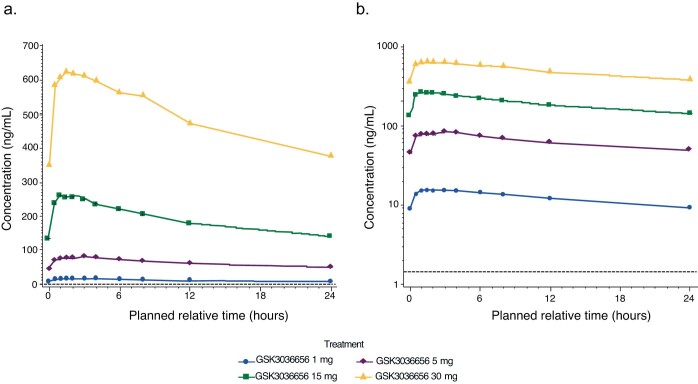
Mean GSK3036656 (ganfeborole) plasma concentration–time plots at Day 14. a, linear and b, semi-logarithmic scale. Pharmacokinetics population. (GSK3036656 (ganfeborole) 1 mg, N = 9; GSK3036656 (ganfeborole) 5 mg, N = 17; GSK3036656 (ganfeborole) 15 mg, N = 15; GSK3036656 (ganfeborole) 30 mg, N = 15).

The absorption of ganfeborole was fast, with a median time to the maximum concentration (*T*_max_) ranging from 1.00 to 2.49 h across the four groups (Table 3). Maximum concentration (*C*_max_) and area under the plasma concentration–time curve from treatment initiation to 24 h (AUC_0–24_) were approximately dose proportional for doses 1–15 mg, and a more than dose-proportional increase in AUC_0–24_ was observed at the 30 mg dose (Table 3 and Extended Data Fig. 1). *C*_max_ and AUC_0–24_ displayed low-to-moderate variability (Table 3).

**Table 3:** Summary of PK parameters at day 14 (PK population)

	GSK3036656 (ganfeborole)			
PK parameter	1 mg	5 mg	15 mg	30 mg
	*n* = 8	*n* = 14	*n* = 14	*n* = 13
AUC_0−24_ (ng*h/ml), geometric mean (% CVb)	282.1 (25.8)	1,497.1 (12.4)	4,493.9 (12.5)	11,540.0 (17.8)
*C*_max_ (ng/ml), geometric mean (% CVb)	16.93 (23.7)	94.60 (24.7)	291.41 (18.9)	705.24 (20.2)
*T*_max_ (h), median (range)	1.50 (0.50, 6.00)	2.49 (0.50, 4.00)	1.00 (0.50, 4.00)	2.00 (0.50, 8.00)

All GSK3036656 (ganfeborole) cohorts received a loading dose on day 1.

AUC_0–24_, area under the plasma concentration–time curve from treatment initiation to 24 h; *C*_max_, maximum concentration; CVb, inter-subject variability; PK, pharmacokinetics; *T*_max_, time to the maximum concentration.

### Post hoc exploratory analyses

#### PET/CT scans

**Fig. 3:**
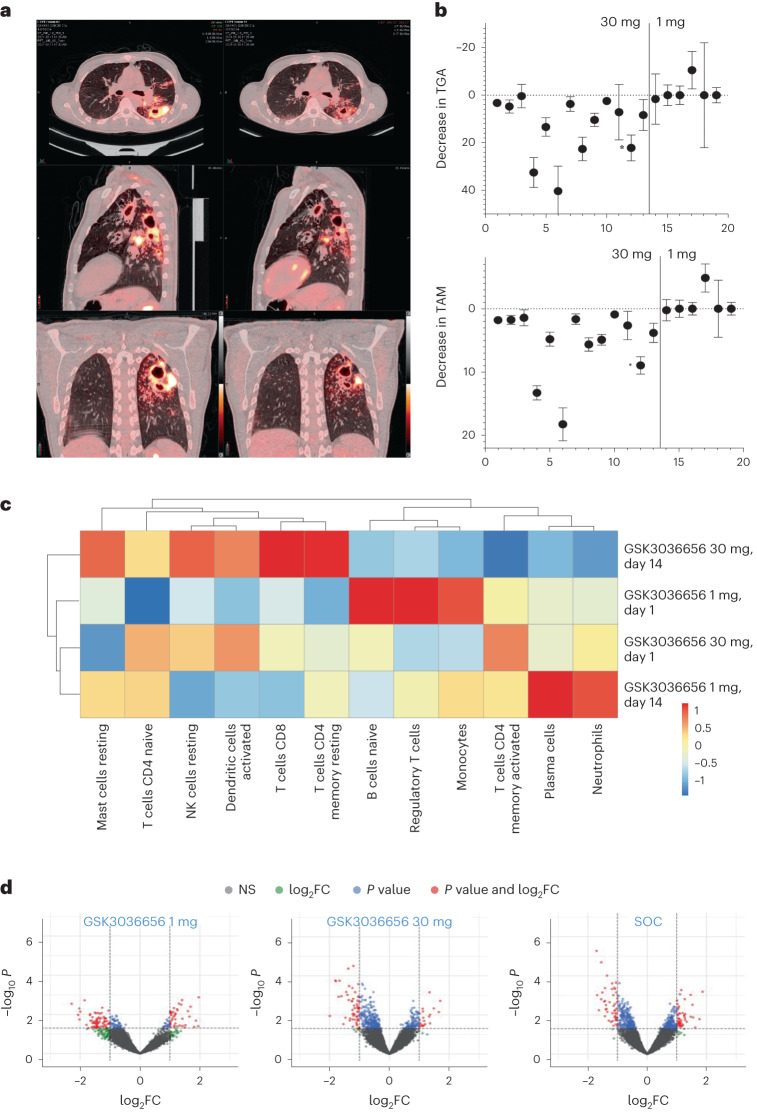
Post hoc exploratory analyses: ^18^F-FDG-PET/CT scans and transcriptional profiling. **a**, Representative ^18^F-FDG-PET/CT scan of a participant receiving GSK3036656 (ganfeborole) 30 mg at baseline (left column) and after 14 days of treatment (right column). Both scans are shown at a maximum scale of SUV 7. The axial slices shown at the top indicate the rapid response of a peri-cavitary lesion in the left inferior lobe, the sagittal slices shown in the middle indicate the response in cavities located in the superior and inferior lobes, and the coronal slices shown at the bottom indicate the response in and around the largest cavitary feature in this participant. **b**, Top: changes in TGA for each participant, computed from the sum of the body weight-adjusted SUV values per voxel, at day 14 versus baseline for those receiving GSK3036656 (ganfeborole) 30 mg (left) and GSK3036656 (ganfeborole) 1 mg (right). Bottom: changes in TAM, computed from the individual HU value per voxel + 1,000 to adjust for negative air values in the same participants. Data marked with an asterisk are from the participant illustrated in **a**. *N* by subject from left to right in the graph: *N* = 4,470, *N* = 4,824, *N* = 3,310, *N* = 4,916, *N* = 6,120, *N* = 755, *N* = 3,922, *N* = 1,459, *N* = 2,205, *N* = 2,826, *N* = 2,272, *N* = 1,626, *N* = 1,440, *N* = 2,531, *N* = 3,039, *N* = 7,701, *N* = 921, *N* = 944 and *N* = 3,971. Data are presented as mean values (± standard error of the mean). **c**, Heatmap of CIBERSORT gene expression profiles to estimate frequencies of each cell type. Mean values between participants in two groups: (1) those receiving GSK3036656 (ganfeborole) 1 mg; and (2) those receiving GSK3036656 (ganfeborole) 30 mg. **d**, Differential gene regulation at day 14 versus baseline for participants receiving GSK3036656 (ganfeborole) 1 mg (*n* = 7), GSK3036656 (ganfeborole) 30 mg (*n* = 13) and SOC (*n* = 13). Green indicates high log FC, but not significant, blue indicates significant but relatively low log FC, red indicates significant and high log FC, and gray indicates not significant, low log FC. ^18^F-FDG-PET/CT, ^18^F-fluorodeoxyglucose positron emission tomography/computed tomography; CD, cluster of differentiation; FC, fold change; HU, Hounsfield units; NK, natural killer; NS, not significant; SOC, standard of care; SUV, standardized uptake value; TAM, total associated mass; TGA, total glycolytic activity.

**Extended Data Fig. 2:**
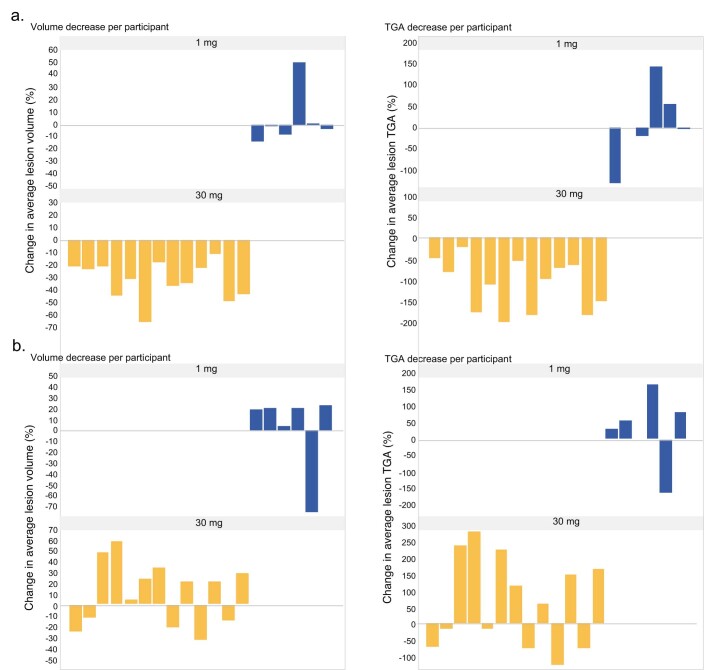
Impact of GSK3036656 (ganfeborole) by lesion density per participant. a, Response in cubes containing <20% voxels corresponding to air (N = 55,682). b, Response in cubes containing >20% voxels corresponding to air (cavity walls) (N = 3570). The GSK3036656 (ganfeborole) 1 mg group (N = 6) is depicted at the top of the graphs (blue) and the 30 mg group (N = 13) at the bottom (yellow); each bar represents one participant (participants 1–19); bars below 0 indicate positive change in average lesion volume or lesion TGA. TGA, total glycolytic activity.

**Extended Data Fig. 3:**
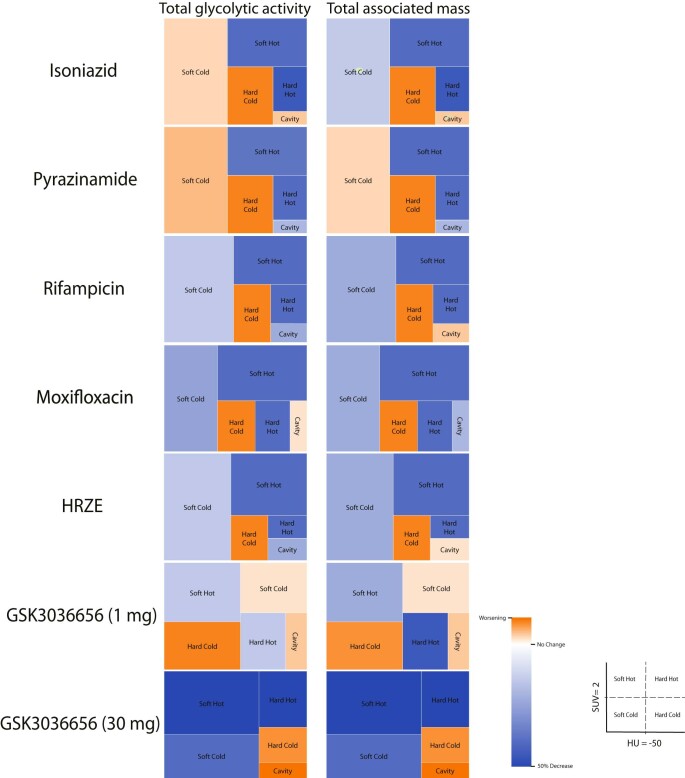
PET/CT scan summary response measures by lesion feature. Changes in TAM, computed from the individual HU value per voxel +1000 to scale adjust for negative air values, and total glycolytic activity, computed from the sum of the body weight-adjusted SUV values per voxel, at Day 14 versus baseline for first-line TB drugs, moxifloxacin (recomputed from Xie, et al.^27^ to adjust to the same voxel size) and GSK3036656 (ganfeborole) 1 mg and 30 mg. Lesion cubes were divided based on baseline features into five categories based on the indicated HU and SUV thresholds as well as the presence of more than 10% voxels corresponding to −1000 HU (indicating air), which represent the walls of cavities. Lesion features were ‘hard’ (>−50 HU) and ‘soft’ <−50 HU with a SUVmean >2 and were grouped by baseline PET values < or >2 as ‘hot’ and ‘cold’; cubes that contained >10% air were considered ‘cavities’. The size of the block represents the total volume of disease with those characteristics across all of the participants receiving that particular treatment. HRZE, rifampicin, isoniazid, pyrazinamide and ethambutol; HU, Hounsfield units; PET/CT, positron emission tomography/computed tomography; SUV, standardized uptake value; TAM, total associated mass; TB, tuberculosis; TGA, total glycolytic activity.

PET/CT scans of the lungs were obtained for 19 participants (Extended Data Table 2; ganfeborole 1 mg, *n* = 6 (PET/CT scans were not possible for all eight participants in this group as there were no available slots for the scans within the permitted timeframe); 30 mg, *n* = 13). Baseline and day 14 scans were computationally segmented and aligned at the point where the trachea splits into the main bronchi, and matched cubes of lesion material (0.68 × 0.68 × 0.5 mm) were evaluated. As a representative example, Fig. 3a shows aligned scans from one participant to show changes in three planes. Treatment with ganfeborole 30 mg was associated with a reduction in lesion-associated radiodensity across aggregated matched cubes in all participants and to a reduction in lesion total glycolytic activity in 11/13 participants (Fig. 3b). In contrast, participants receiving ganfeborole 1 mg, which demonstrated no EBA, showed unchanged or increasing lesion volume and total glycolytic activity (Fig. 3b). Ganfeborole 30 mg had the largest impact on lesions with high baseline fluorodeoxyglucose uptake, such as consolidations and infiltrates (Extended Data Fig. 2a), and a lower but appreciable impact on cavity walls (Extended Data Fig. 2b). To further explore the PET/CT scan features, we computationally aligned and analyzed 311 mm^3^ voxel groupings across participants receiving ganfeborole 30 mg and compared the changes in features observed with results from a previous 14-day EBA study of participants with pulmonary TB, which included four monotherapy groups^27^ (Extended Data Fig. 3). When lesions were stratified according to their baseline characteristics, ganfeborole appeared qualitatively to be most similar in activity to rifampicin and moxifloxacin^27^, acting on both ‘hard’ (≥50 Hounsfield units [HU]) and ‘soft’ (≤50 HU) lesion features with a standardized uptake value (SUV) mean >2. In addition to dividing lesion features into ‘hard’ and ‘soft’, we also grouped them by baseline PET values <2 or >2 as ‘hot’ and ‘cold’; cubes that contained >10% air were considered ‘cavities’. Ganfeborole appeared to have the strongest impact on lesions that were ‘hot’ regardless of initial CT density.

#### Transcriptional profiling

At the blood RNA level, the strongest differences in gene expression after 14 days of therapy compared with baseline were in phagocytosis, adaptive immune system and humoral immune response, and complement activation with ganfeborole 1 and 30 mg. With ganfeborole 30 mg, further differences were detectable in neutrophil degranulation, neutrophil activation and neutrophil-mediated immunity.

Whole blood samples from participants treated with ganfeborole 30 and 1 mg were compared at baseline and day 14 using a gene set enrichment analysis (ClusterProfiler) to determine the up- and downregulated pathways. Fig. 3c shows the results of the CIBERSORT algorithm based on transcriptomic profiles from whole blood for deriving cell fractions for participants treated with ganfeborole 30 mg and ganfeborole 1 mg at baseline and after 14 days of treatment. Participants who received ganfeborole 1 mg showed significantly higher neutrophil activity and plasma cell activity after 14 days than those participants receiving ganfeborole 30 mg after 14 days. While these cell types are significantly more active after 14 days than at the start of therapy, the activity of these two cell types at ganfeborole 30 mg after 14 days is significantly lower than at the start of therapy. In contrast, the cluster of differentiation (CD)8 T cells showed a significantly higher activity at ganfeborole 30 mg after 14 days than at baseline and at ganfeborole 1 mg after 14 days; here, too, there appears to be a downregulation of the T cell CD8 cells after 14 days with 1 mg. The situation was shown to be similar with the natural killer cells. However, the initial analysis using the CIBERSORT algorithm was limited to predefined cell patterns; hence, no conclusions about the involvement or dysfunctionality of specific cells can be made. The subsequent enrichment analysis was not limited to predefined immune-related pathways but includes the broad functionality of a human’s immune system. Fig. 3d shows the differential gene regulation at day 14 versus baseline.

## Discussion

In this study, daily treatment with ganfeborole at 5, 15 and 30 mg was associated with EBA, as measured by the rate of change in log_10_CFU and TTP over 14 days in participants with rifampicin-susceptible TB. The 30 mg dose displayed the highest EBA across the ganfeborole groups, whereas the 1 mg dose showed no EBA. The SOC results were in line with what would be expected.

Ganfeborole was well tolerated. No SAEs were reported, and AE rates did not appear to be dose dependent. The most frequently reported AEs in participants receiving ganfeborole were hemoptysis and pruritus; however, incidence rates were low. Hemoptysis was not deemed drug related in any of the cases, and no notable findings with respect to coagulation parameters or platelet counts were seen. Furthermore, the rate of hemoptysis observed was consistent with that commonly detected in the TB population^28^. Pruritus was considered drug related in 2/7 participants (5 mg, *n* = 1; 15 mg, *n* = 1). Due to the small sample size, conclusions cannot be drawn as to the reasoning for the occurrence of hemoptysis and pruritus.

Ganfeborole absorption was fast; plasma PK parameters were approximately dose proportional for doses 1–15 mg, with a higher degree of accumulation observed at 30 mg. These data are in line with the phase 1 PK study results^22^, which demonstrate similar PK characteristics between healthy subjects and participants with TB.

Dose ranging was employed to examine the relationship between dose/exposure and EBA, and to select an optimal dose for further clinical progression. The starting dosage in the study was 5 mg QD, with subsequent selection of doses based on emerging PK data from previous cohorts and derivation of probability of target attainment to ensure that participants receiving the next dose level would not exceed the predefined safety exposure limits. The totality of the clinical pharmacology evidence in the ganfeborole program and in silico PK/PD simulations describing the PD relevance of ganfeborole’s clinical exposures to achieve PD markers within the site of action (TB lesions) (unpublished data) suggest that ganfeborole 20 mg QD would be the optimal dosage to be used in future combination therapy studies. In this study, a full dose–response curve was explored, supporting this type of a study as a dose finding tool of novel anti-TB agents.

On PET/CT scans (post hoc exploratory analysis), ganfeborole appeared to show the strongest impact on lesions that were ‘hot’ regardless of initial CT density, consistent with a large impact on airway infiltrates surrounding cavities that are primarily bronchial and largely thought to be composed of infiltrating neutrophils at the airway surface^29^. Consistent with this, correlation of the transcriptional profiles from peripheral blood with the change in PET/CT values revealed a strong association with neutrophil-dominated transcriptional modules. Although only conducted in a small number of participants, taken together, these results suggest that ganfeborole acts in concert with the inflammatory response to affect containment of the bacteria.

This study has several limitations. The study design did not include a negative control as it is unethical to have a no treatment or placebo group of participants with active TB. All participants received SOC after 14 days in the study. SOC was used as a benchmark laboratory control and demonstrated EBA within the expected range. As a combination therapy, SOC is not directly comparable with ganfeborole monotherapy, and no statistical comparison was performed. Data were generated in participants with drug-susceptible TB, and although resistant bacilli may be affected by biological fitness costs^30^, these effects are believed to hold validity for drug-resistant TB as well, since bacterial mutations conferring drug resistance to other agents are not expected to affect susceptibility to ganfeborole due to its different mechanism of action, and EBA and tolerability are expected to be translatable to participants with resistance to other agents. The activity of ganfeborole needs to be further explored in longer studies and in combination regimens, including participants with TB resistant to current agents and those receiving co-medication with antiretroviral drugs. Additionally, in this study, all participants were male due to preclinical teratogenicity data that became available after the phase 1 study. Subsequent studies recruiting female participants have been initiated including NCT05382312, which includes female participants of childbearing potential using non-user-dependent contraception. Finally, all participants were Black, which (although not atypical for the TB population in South African clinics^31^) may challenge the generalizability to other populations; thus, additional testing in other populations is required. Nevertheless, a previous population PK analysis showed that race was not a significant covariate on the PK of ganfeborole (data on file), suggesting that similar results would be expected in other populations.

A new pan-TB treatment regimen, consisting of novel agents to which no drug resistance exists, is a goal of the World Health Organization, with drugs that strike a balance between antimycobacterial activity, safety and duration of treatment^17^. Ganfeborole offers the possibility of a low-dose QD treatment for TB, with a low drug–drug interaction potential^32^. Overall, the novel mechanism of action of ganfeborole, demonstrated EBA, and acceptable safety and tolerability profile suggest this agent is a potential candidate for a future combination treatment to address the TB epidemic. Future studies will aid in selecting the optimal ganfeborole duration of treatment and appropriate partner agents to complement its anti-TB action.

## Methods

### Study design

This single-center, open-label, randomized phase 2a study was conducted from March 2019 to December 2021 at the TASK clinical research center in Cape Town, South Africa (ClinicalTrials.gov registration NCT03557281). Participants were recruited in four cohorts and randomized 3:1 to receive GSK3036656 (ganfeborole) or SOC for drug-susceptible TB. The ganfeborole dose for each cohort was determined using data (safety, tolerability and PK, as well as activity, if available) from the prior cohort(s) to proceed to each subsequent dose level. The study protocol was reviewed and approved by a national ethics committee or institutional review board, in accordance with the International Council for Harmonisation of Technical Requirements for Pharmaceuticals for Human Use guideline for good clinical practice and applicable country-specific requirements. The study was conducted in accordance with ethical principles that have their origins in the Declaration of Helsinki and the principles of good clinical practice. Ethical approval was granted by Pharma-Ethics, South Africa. Regulatory approval was granted by the South African Health Products Regulatory Authority. The first participant first visit was March 22, 2019, and the last participant first visit was November 16, 2021.

Protocol amendments included the following: the addition of a minimum age requirement of 25 years for participation in the PET/CT subgroup; a stricter minimum renal function requirement for eligibility due to emergence of new clinical data showing the main route of clearance for ganfeborole is renal^22^; and sample size re-estimations due to the observed variability in the rate of change of log_10_CFU being lower than expected. We consider it unlikely that these amendments influenced the study outcomes.

### Participants

TASK is a dedicated facility for TB clinical studies, recruiting trial participants from local clinics. All participants provided written informed consent before the performance of any study-specific procedures.

Participants were eligible if they were male, aged 18–65 years, weighed 40–90 kg and had a new episode of untreated, rifampicin-susceptible pulmonary TB. All participants were male due to preclinical teratogenicity data from animal models that became available after the phase 1 study. The presence of a chest X-ray consistent with TB, and at least one sputum sample positive on direct microscopy for acid-fast bacilli or molecular test either medium or high positive for *M.* *tuberculosis*, normal troponin and creatinine clearance ≥75 ml/min (Cockroft–Gault formula) were required. Further inclusion criteria included a normal echocardiogram or echocardiogram with normal left ventricular function with at most trace to mild valvular regurgitation and no valvular stenosis; in preclinical studies in dogs (unpublished data), adverse heart valvular and vascular pathology effects were detected at very high doses and multiples of the clinical exposures. These findings were not reproduced in longer toxicology studies or in the first-in-human study^22^. To mitigate risk in clinical studies, echocardiograms were performed to exclude participants with pre-existing valve or other cardiac abnormalities, and to detect the presence of any abnormalities after study completion.

Participants were excluded if they presented with clinically significant evidence of extra-thoracic TB, an electrocardiogram QT interval (corrected by Fridericia’s correction formula) >450 ms, or history of allergy to any of the trial investigational products or related substances as confirmed by the investigator. Due to some skin pigmentation being reported in preclinical studies in dogs, participants with vitiligo were also excluded; the exclusion of these participants and regular visual examination of the skin throughout the study meant that development of any skin pigmentation was monitored. Participants with human immunodeficiency virus infection with a CD4^+^ count <350 cells/µl, who had received antiretroviral or antifungal therapy in the past 30 days, or with acquired immunodeficiency syndrome-defining illness were also excluded.

As it is recognized that the risk from medical radiation is higher in younger age groups, participants completing the PET/CT scan assessments were required to be aged ≥25 years at the time of providing informed consent.

### Randomization and masking

Within each cohort, participants were randomized 3:1 to receive ganfeborole or SOC for drug-susceptible TB using permutated blocks. On or before day 1, eligible participants were assigned a unique randomization number in ascending numerical order at the study site. The randomization number encoded the participant’s assignment to ganfeborole or SOC, according to the randomization schedule generated before the study. The study was open label, so neither the participants nor the primary investigators were blinded. However, all laboratory staff involved in analyzing and reporting the log_10_CFU counts and TTP endpoints results were blinded to treatment allocation.

### Treatment and assessments

Eligible participants were screened within 9 days before receiving the first dose. Participants orally received either SOC or ganfeborole 1–30 mg QD. SOC (Rifafour e-275 or generic alternative) was weight adjusted per the South African National Tuberculosis Management Guidelines 2014 (ref. ^33^). Each tablet contained 150, 75, 400 and 275 mg of rifampicin, isoniazid, pyrazinamide and ethambutol, respectively. The SOC group was included as a benchmark for the EBA quantitative mycobacteriology and to evaluate whether the SOC drugs in this population give similar EBA results to those demonstrated in prior studies with this combination. All participants received SOC after 14 days in the study.

A dose-escalation approach was used, whereby preliminary safety, tolerability and PK data from the previous cohort were reviewed by the Dose Escalation Committee before selecting the next cohort dose; efficacy data were also considered, if available. Dose escalation was based on population PK modeling and probabilistic analysis to ensure that a proposed new dose would not exceed the predefined safety exposure margins. In addition, dose escalation was not allowed to be higher than three-fold. Historically, doses in phase 2a proof-of-concept trials are selected to maximize bactericidal activity, resulting in doses located at the top plateau of the dose–response curve. Delineation of the dose–response curve in phase 2a trials may guide selection of the phase 2b dose based on exposure–response relationships for activity and safety. Understanding how safety and efficacy might be affected by changes in ganfeborole exposure is vital before advancing into combination trials where potential drug–drug interactions and concomitant medications might impact clinical exposures. In this study, dose cohorts proceeded sequentially, with safety, tolerability and PK data from the previous cohort reviewed before selecting the dose for the next cohort. The protocol allowed for up to five dose cohorts and selection of a dose of ganfeborole that was lower than the dose in the previous cohort, based on the available data. Following review of data from the 5, 15 and 30 mg cohorts, a dose of 1 mg was selected for the final cohort to fully delineate the dose–response curve. The sequential cohorts received ganfeborole as follows:

- Cohort 1, the ganfeborole group (5 mg group) received a 15 mg loading dose on day 1 and 5 mg QD maintenance doses on days 2–14.
- Cohort 2, the ganfeborole group (15 mg group) received a 30 mg loading dose on day 1 and 15 mg QD maintenance doses on days 2–14.
- Cohort 3, the ganfeborole group (30 mg group) received a 75 mg loading dose on day 1 and 30 mg QD maintenance doses on days 2–14.
- Cohort 4, the ganfeborole group (1 mg group) received a 3 mg loading dose on day 1 and 1 mg QD maintenance doses on days 2–14.

On day 15, after the study treatment was completed, all participants initiated SOC.

Serial overnight sputum samples were collected initially daily (baseline to day 4), followed by alternate days (days 6–14). Median daily decrease in CFU/ml sputum (solid culture) (EBA CFU_0–14_) and increase in TTP in liquid media were estimated with mixed-effects modeling.

Safety assessments (monitoring of AEs, clinical laboratory tests, vital signs, electrocardiograms, physical examinations and echocardiograms) were carried out at the site from the start of the study treatment until the follow-up visit (day 28). Blood samples were collected (single pre-dose on days 12 and 13 and serial samples on day 14) for measurement of plasma concentrations and PK of ganfeborole and for RNA sequencing analysis.

PET/CT scans were performed in eligible participants at baseline and day 14 (1 and 30 mg cohorts only). A final follow-up visit took place on day 28 to collect safety data and ensure ongoing engagement with the healthcare facility.

### Objectives and endpoints

The primary objective of the study was to determine the EBA of ganfeborole QD over 14 days of repeat dosing, as assessed by the rate of change in log_10_CFU/ml counts per ml of sputum from baseline to day 14 (EBA CFU_0–14_).

The secondary EBA objectives were to determine the EBA of ganfeborole QD over the first 2 days and the last 12 days. They were assessed by the change in log_10_CFU/ml sputum from baseline to day 2 (EBA CFU_0–2_) and day 2 to day 14 (EBA CFU_2–14_), and by the rate of change in TTP from baseline to day 14 (EBA TTP_0–14_), baseline to day 2 (EBA TTP_0–2_), and day 2 to day 14 (EBA TTP_2–14_).

Additional secondary objectives were to evaluate the safety and tolerability of ganfeborole QD over 14 days, determined by AE reporting and monitoring of clinical laboratory values, vital signs and electrocardiogram parameters. Furthermore, the aim of the study was to characterize the PK of ganfeborole in participants following repeat QD dosing for 14 days. PK parameters of interest were derived using non-compartmental analysis including AUC, *C*_max_ and *t*_max_.

Characterizing the radiological features of TB lesions by PET/CT scan to detect treatment-induced changes was a pre-planned exploratory objective. Changes in lesion volume, total glycolytic activity and impact on extent of disease as measured by lesion characteristics (consolidations, infiltrates and cavities) were determined. Furthermore, the changes in PET/CT signal were correlated with the RNA profiles of the participants at baseline and day 14.

### Statistical analysis

The original estimated enrollment totaled 100 participants. As no formal statistical hypothesis was tested, no formal sample size calculation was performed. However, simulations were conducted to determine the impact of the size of the study on the ability to detect a treatment effect based on the mean daily decline from baseline in log_10_CFU/ml sputum in participants treated with ganfeborole. Based on earlier EBA studies, a standard deviation (s.d.) of 0.2 was used for the change from baseline in log_10_CFU for the simulations. Assuming a true underlying change from baseline of −0.06 and a sample size of 15 participants per group, there was approximately 90% probability of the observed mean change being a negative value (that is, a positive response indicated by a decline in bacterial load being observed). Due to a slower than anticipated rate of recruitment (during the COVID-19 pandemic), a review of the s.d. of daily change in log_10_CFU data from participants in the first two cohorts concluded that the observed s.d. was smaller than that used in the initial sample size simulation. Based on subsequent simulations, it was estimated that assuming a true underlying change from baseline of −0.06, an s.d. of 0.15, and a sample size of nine participants per group, there was approximately 90% chance of the observed mean change being a negative value; therefore, nine participants were selected for ongoing and future ganfeborole cohorts.

The primary and secondary efficacy analyses were performed using a mixed model repeated measures analysis. The model included covariates for treatment, time, BMI and treatment by time. Baseline bacterial load values (log_10_CFU or TTP) were included as covariates for the models for days 2–14. Summaries of the mean daily rate of change and associated 95% confidence were presented for each treatment group. Baseline was defined as the mean of day −2 and day −1; if data were available at only one of these time points, that value was used as baseline. A bilinear Bayesian regression model was also used for EBA CFU and TTP for baseline to Day 14; this model is recommended for the analysis of EBA endpoints in TB because they can follow a bilinear rate of change. The model incorporated covariates for intercept, BMI, treatment, treatment by day before node, treatment by day after node and participant. The node is the inflection point of the curve and was independent for each treatment.

Safety data were summarized descriptively according to the Medical Dictionary for Regulatory Activities version 24.1 and GSK’s Integrated Data Standards Library standards.

PET/CT scan images were segmented and analyzed manually and computationally. Analysis was blind to dose. For the manual analysis, which was carried out by an appropriately trained reader, regions of interest spanning the entire left or right lung were defined in MIM Maestro (6.7.12). PET/CT scan images were scored by two or more independent observers blinded to treatment arm. Custom segmentation software was used for the computational analysis. Segmentation masks were created and applied to the thoracic cavity representing air spaces, including cavitary air and pneumothorax, the pleural surface and mediastinum, the trachea and large airways, normal lung areas, vasculature of the lung and TB-related lesions. Baseline and day 14 scans were computationally registered on the basis of non-deformed scans and were subdivided into ‘cubes’ that were 11 voxels in each dimension.

For the analysis of the RNA data, the count matrix was first created. This formed the basis for the application of the CIBERSORT algorithm (https://cibersortx.stanford.edu/). Furthermore, an enrichment analysis was performed to distinguish between participants who showed improvement in PET/CT signal and those who showed deterioration. The 50 genes with the strongest effect to discriminate between participants with improvement and deterioration were assigned using Kyoto Encyclopedia of Genes and Genomes pathway identifiers^34^ to link them to their respective pathways. Data are presented as stated in the relevant tables and figures.

### Reporting summary

Further information on research design is available in the Nature Portfolio Reporting Summary linked to this article.

## Online content

Any methods, additional references, Nature Portfolio reporting summaries, source data, extended data, supplementary information, acknowledgements, peer review information; details of author contributions and competing interests; and statements of data and code availability are available at 10.1038/s41591-024-02829-7.

### Supplementary information

Supplementary Information Study protocol.

Reporting Summary

## Figures and Tables

**Extended Data Table 1:**
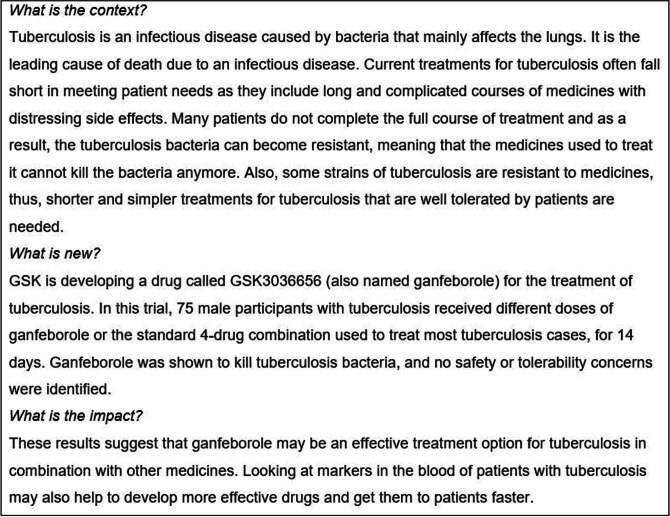
Plain Language Summary

A plain langauge summary which describes the study and its outcomes in langauge suitable for a lay audience.

**Extended Data Table 2:**
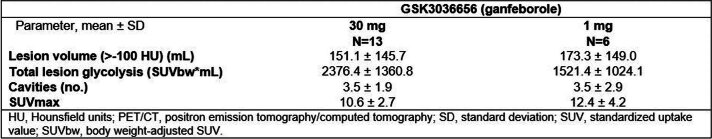
Baseline radiologic characteristics of participants in the PET/CT post-hoc exploratory analysis

This table includes the baseline radiologic characteristics, such as the lesion volume and number of cavities, of 19 participants included in the PET/CT post-hoc exploratory analysis.

## Data Availability

GSK is committed to provide access to anonymized patient-level data that sit behind the results of our clinical trials. External researchers can request access to anonymized patient-level clinical trial data and supporting clinical trial documents through either of two multi-sponsor data sharing portals. Consistent with the PhRMA-EFPIA Principles for Responsible Data Sharing and with expectations of good scientific practice, researchers can request access to our studies by providing a research proposal with a commitment to publish their findings. Researchers whose requests are approved by an independent panel and accepted by GSK are provided access to data in a secure environment upon signing a Data Sharing Agreement (DSA). For more details, please refer to GSK weblink to access GSK’s data sharing policies and as applicable seek anonymized subject level data via the link https://www.gsk-studyregister.com/en/.
