# Supplementary material for: A first-in-class leucyl-tRNA synthetase inhibitor, ganfeborole, for rifampicin-susceptible tuberculosis: a phase 2a open-label, randomized trial
Source: Nat Med. 2024 Feb 16;30(3):896–904. doi: 10.1038/s41591-024-02829-7 (PMC10957473; doi:10.1038/s41591-024-02829-7)
Supplement: Supplementary file 1 — Study protocol. [file 41591_2024_2829_MOESM1_ESM.pdf]

nature medicine

Article

https://doi.org/10.1038/s41591-024-02829-7

A first-in-class leucyl-tRNA synthetase inhibitor, ganfeborole, for rifampicin-susceptible tuberculosis: a phase 2a open-label, randomized trial

In the format provided by the authors and unedited

TITLE PAGE

Protocol Title: A Phase IIa open-label trial to investigate the early bactericidal activity, safety and tolerability of GSK3036656 in participants with drug-sensitive pulmonary tuberculosis.

Protocol Number: 201214 / Amendment 05

Short Title: GSK3036656 EBA Study

**Compound Number:**	GSK3036656

Sponsor Name and Legal Registered Address:

GlaxoSmithKline Research & Development Limited
980 Great West Road
Brentford
Middlesex, TW8 9GS
UK

Medical Monitor Name and Contact Information can be found in the Study Reference Manual

Approval Date: 12-MAY-2021

Copyright 2021 the GlaxoSmithKline group of companies. All rights reserved. Unauthorised copying or use of this information is prohibited.

SPONSOR SIGNATORY:

Protocol Title: A Phase IIa open-label trial to investigate the early bactericidal activity, safety and tolerability of GSK3036656 in participants with drug-sensitive pulmonary tuberculosis.

Protocol Number: 201214 /Amendment 05

**Compound Number:**	GSK3036656

Dr Alison Webster

Head, Global Health Clinical

Date

The signed page is a separate document.

Medical Monitor Name and Contact Information [can be found in the Study Reference Manual]

PROTOCOL AMENDMENT SUMMARY OF CHANGES TABLE

DOCUMENT HISTORY		
**Document**	**Date**	**DNG Number**
Amendment 5	*12-MAY-2021*	*TMF-13331420*
Amendment 4	*17-FEB-2021*	*TMF-11820616*
Amendment 3	*24-JUL-2019*	*2017N332005_03*
Amendment 2	*24-SEP-2018*	*2017N332005_02*
Amendment 1	*20-FEB-2018*	*2017N332005_01*
Original Protocol	*09-FEB-2018*	*2017N332005_00*

Amendment 5: 12-MAY-2021

Overall Rationale for the Amendment:

Variability in the rate of change of log_{10}CFU observed in the trial to-date is lower than anticipated. Due to this, and slower than anticipated recruitment, the study sample size has been re-calculated. The potential to modify the haemoglobin exclusion and stopping criteria in subsequent cohorts investigating lower doses of GSK3036656 has been added.

Further changes made to the protocol are detailed in the table below.

Section #	Description of Change	Brief Rationale
Section 1 Synopsis	Changed from:<br>"Up to 5 cohorts; 20 participants in each cohort (15 participants will receive GSK3036656, and 5 participants will receive SoC in each cohort)."<br><br>To:<br>"Up to 5 cohorts; 12-20 participants in each cohort (9-15 participants will receive GSK3036656, and 3-5 participants will receive SoC in each cohort)."	Updated to reflect changes in number of participants due to sample size re-calculations
Section 3.3.1 Risk Assessment	Changed from:<br>"Participants whose haemoglobin drops below pre-specified limits (<8.0 g/dL) will be withdrawn from the study (Section 8.1.3)."<br><br>To:<br>"Participants whose haemoglobin drops below pre-specified limits will be withdrawn from the study (Section 8.1.3)."	Removed specific value (<8.0 g/dL) as cross reference to Section 8.1.3 provides further information
Section 5.1 Overall Design	Changed from:<br>"The study will comprise up to 5 cohorts of participants. It is intended that 15 participants in each cohort will receive GSK3036656 and 5 will receive standard-of-care regimen for DS-TB (i.e. Rifafour e-275 or equivalent generic alternative)."<br><br>To:<br>"The study will comprise up to 5 cohorts of participants. It is intended that 9-15 participants in each cohort (dependent on the cohort) will receive GSK3036656 and 3-5 will receive standard-of-care regimen for DS-TB (i.e. Rifafour e-275 or equivalent generic alternative), with a randomisation ratio of 3:1"	Updated to reflect changes in number of participants due to sample size re-calculations
Section 5.1 Overall Design	Changed from:<br>"Cohorts will proceed sequentially starting A through D"<br><br>To:<br>"Cohorts will proceed sequentially starting A through E"	Corrected to align with study design of up to 5 cohorts

Section #	Description of Change	Brief Rationale
Section 5.1 Overall Design	Changed from:<br>"It is anticipated that it may be necessary to administer a dose of GSK3036656 the same or lower than in a previous cohort, dependent on preliminary safety, tolerability and pharmacokinetic data"<br><br>To:<br>"It is anticipated that it may be necessary to administer a dose of GSK3036656 the same or lower than in a previous cohort, dependent on preliminary safety, tolerability, pharmacokinetic or efficacy data"	Corrected to align with text in Section 11, Dose Escalation.
Section 5.3 Number of Participants	Changed from:<br>"Up to 20 participants will be enrolled into each cohort."<br><br>To:<br>"It is intended that 12- 20 participants will be enrolled into each cohort, dependent on the cohort."	Updated to reflect changes in number of participants due to sample size re-calculations
Section 5.3 Number of Participants	Changed from:<br>"If participants prematurely discontinue the study, additional replacement participants may be recruited at the discretion of the Sponsor in consultation with the Investigator."<br><br>To:<br>"If participants prematurely discontinue the study, or if their respiratory sputum cultures are contaminated, additional replacement participants may be recruited at the discretion of the Sponsor in consultation with the Investigator."	The primary endpoint for this study is derived from respiratory sputum cultures. If sputum cultures are contaminated, critical data for study analysis could be lost

Section #	Description of Change	Brief Rationale
Section 6.1 Inclusion Criteria	Changed from:<br>"6. Ability to produce an adequate volume of sputum as estimated from an overnight sputum collection sample (estimated 10 ml or more). (Estimated from a spot sputum sample and confirmed with the first overnight collection. If less than 10 ml is collected overnight this may be repeated once)."<br><br>To:<br>"6. Ability to produce an adequate volume of sputum as estimated from an overnight sputum collection sample (estimated 10 mL or more). (If less than 10 mL is collected overnight this may be repeated once)."	A random spot sputum sample does not provide a good estimation of a participant's ability to produce at least 10 mL sputum compared to a 16-hour overnight collection period, thus this sentence has been removed
Section 6.2 Exclusion Criteria	Changed from:<br>"13. Participants with the following abnormal laboratory values at screening as graded by the enhanced Common Terminology Criteria for Adverse Events (CTCAE v 5 2017):<br><br>a. haemoglobin <10.0 g/dL;"<br><br>To:<br>"13. Participants with the following abnormal laboratory values at screening as graded by the enhanced Common Terminology Criteria for Adverse Events (CTCAE v 5 2017):<br><br>a. haemoglobin <10.0 g/dL; this criterion may be adjusted following DEC review (see Section 11)."	Cross reference to Section 11 has been added and provides further details that the Dose Escalation Committee are empowered to make regarding haemoglobin exclusion criteria
Section 8.1.3 Haematology Stopping Criteria	Changed from:<br>"A reduction in haemoglobin to below 8.0 g/dL"<br><br>To:<br>"A reduction in haemoglobin to below 8.0 g/dL; this criterion may be adjusted following DEC review (see Section 11)."	Cross reference to Section 11 has been added and provides further details that the Dose Escalation Committee are empowered to make regarding haemoglobin stopping criteria

Section #	Description of Change	Brief Rationale
Section 9.1 Microbiological Efficacy/Activity Assessments	The following text was added:<br>"MIC data reporting will likely occur after the clinical database has been authorised for the primary and secondary efficacy and safety endpoints at the end of the study (i.e. after Database Freeze [DBF]). The report detailing the data will remain as a separate report and will not be included in the GSK clinical study report."	The lab conducting microbiology assessments are in the process of validating the EUCAST liquid MIC protocol which will be used to determine the MIC. The lab will require additional time to validate the EUCAST MIC protocol, so the MIC data will be reported outside of the clinical study report
Section 9.4 Vital Signs	Changed from:<br>"Vital signs (to be taken before blood collection for laboratory tests) will consist of at least 1 pulse and blood pressure measurement. Where a measurement is abnormal or significantly different from previous measurements, this will be repeated. Where BP and pulse is repeated above, a further 2 readings recorded at least 1 min apart will be obtained for confirmation and recorded in the CRF."<br><br>To:<br>"Vital signs (to be taken before blood collection for laboratory tests) will consist of at least 1 pulse and blood pressure measurement. Where a measurement is abnormal or significantly different from previous measurements, a further 2 readings will be taken. When vital signs are measured in triplicate, values should be taken at least 1 minute but not more than 5 minutes apart and recorded in the CRF."	Updated to specify window for repeat vital signs, as documented in the Study Reference Manual
Section 10.1 Sample Size Determination	Details of Sample Size Re-estimation added (Section 10.1.2) and separated from Original Sample Size Calculations (Section 10.1.1)	Variability in the rate of change of log_{10}CFU observed in the trial to-date is lower than anticipated. Due to this, and slower than anticipated recruitment, the study sample size has been re-calculated.

Section #	Description of Change	Brief Rationale
Section 10.3.1 Activity/ Pharmacokinetic Analyses	For the analysis of the primary endpoint, EBA CFU_{0-14}, and the secondary endpoints, EBA CFU_{0-2}, EBA CFU_{2-14}, EBA TTP_{0-14}, EBA TTP_{0-2} and EBA TTP_{2-14}, the covariates to be included in the mixed model have been changed from dose and baseline value, to treatment, day, BMI and treatment-by-day	A repeated measures analysis, including day as a covariate is more appropriate for measuring the rate of change over the treatment period. Dose has been replaced by treatment, with data for participants receiving standard-of-care included, and BMI has been added due to it's potential influence on the EBA endpoints.
Section 11 Dose Escalation	Changed from:<br>"PK data in at least 10 subjects receiving GSK3036656 and selected safety data from all subjects in a cohort."<br><br>To:<br>"PK data in at least 7 subjects receiving GSK3036656 and selected safety data from all subjects in a cohort"	Based on the PK variability observed in the first two Cohorts, a sample size of 7 will give at least 80% power in the derivation of key PK parameters with an acceptable precision.
Section 11 Dose Escalation	Changed from:<br>"The primary endpoint may be analysed for all available cohorts after the final visit of the last subject on the second consecutive cohort that reaches or exceeds the target PK plasma exposure for efficacy (based on the murine infection model), and subsequent cohorts."<br><br>To:<br>"The primary endpoint may be analysed once data is available for 12 evaluable subjects, as per the sample size re-estimation calculations, on the second consecutive cohort that reaches or exceeds the target PK plasma exposure for efficacy (based on the murine infection model), and subsequent cohorts."	Updated to reflect changes in number of participants due to sample size re-calculations

Section #	Description of Change	Brief Rationale
Section 11 Dose Escalation	The following text was added:<br>"To adjust the haemoglobin exclusion and stopping criteria if no haematological abnormalities are observed in the review of safety data from the highest dose cohort (Cohort 3). For the subsequent cohort(s) receiving a lower dose of GSK3036656, the haemoglobin exclusion criterion will be lowered to <8.0g/dL, and the haemoglobin stopping criterion will be lowered to <6.0g/dL. Patients with a confirmed haemoglobin <8.0g/dL after randomisation will require enhanced observations, with a repeat haemoglobin level and discussion of patient status with the GSK medical monitor."	A high number of screen fails have occurred due to haemoglobin levels being slightly lower than the current exclusion criteria (<10.0 g/dL), which is a common characteristic in this study population.

TABLE OF CONTENTS

PAGE

PROTOCOL AMENDMENT SUMMARY OF CHANGES TABLE 3

1. SYNOPSIS 12

2. SCHEDULE OF ACTIVITIES (SOA) 15

3. INTRODUCTION 19
   3.1. Study Rationale 19
   3.2. Background 19
   3.3. Benefit/Risk Assessment 20
      3.3.1. Risk Assessment 21
      3.3.2. Benefit Assessment 30
      3.3.3. Overall Benefit:Risk Conclusion 30

4. OBJECTIVES AND ENDPOINTS 31

5. STUDY DESIGN 32
   5.1. Overall Design 32
   5.2. Study Schematic 33
   5.3. Number of Participants 33
   5.4. Participant and Study Completion 33
   5.5. Scientific Rationale for Study Design 34
   5.6. Dose Justification 34

6. STUDY POPULATION 36
   6.1. Inclusion Criteria 36
   6.2. Exclusion Criteria 37
   6.3. Lifestyle Restrictions 38
      6.3.1. Meals and Dietary Restrictions 38
      6.3.2. Caffeine, Alcohol, and Tobacco 38
      6.3.3. Activity 39
   6.4. Screen Failures 39

7. TREATMENTS 39
   7.1. Treatments Administered 40
   7.2. Method of Treatment Assignment 41
   7.3. Preparation/Handling/Storage/Accountability 41
   7.4. Treatment Compliance 42
   7.5. Concomitant Therapy 42

8. DISCONTINUATION CRITERIA 42
   8.1. Discontinuation of Study Treatment 43
      8.1.1. Liver Chemistry Stopping Criteria 43
      8.1.2. QTc Stopping Criteria 44
      8.1.3. Haematology Stopping Criteria 44
   8.2. Individual/Cohort Stopping Criteria 44
   8.3. Trial Stopping Criteria 45
   8.4. Withdrawal from the Study 45
   8.5. Lost to Follow Up 45

9. STUDY ASSESSMENTS AND PROCEDURES ... 46
9.1. Microbiological Efficacy/Activity Assessments ... 46
9.2. Adverse Events ... 47
9.2.1. Time Period and Frequency for Collecting AE and SAE Information ... 48
9.2.2. Method of Detecting AEs and SAEs ... 48
9.2.3. Follow-up of AEs and SAEs ... 48
9.2.4. Regulatory Reporting Requirements for SAEs ... 48
9.2.5. Cardiovascular and Death Events ... 49
9.2.6. Pregnancy ... 49
9.3. Treatment of Overdose ... 49
9.4. Safety Assessments ... 50
9.4.1. Physical Examinations ... 50
9.4.2. Vital Signs ... 50
9.4.3. Electrocardiograms ... 50
9.4.4. Echocardiograms ... 51
9.4.5. Clinical Safety Laboratory Assessments ... 51
9.5. Pharmacokinetics ... 52
9.6. Potential Surrogate Disease/Biomarkers ... 52
CCI [redacted] ... 52
... 53
9.7. Incidental findings ... 54

10. STATISTICAL CONSIDERATIONS ... 54
10.1. Sample Size Determination ... 54
10.1.1. Original Sample Size Calculation ... 54
10.1.2. Sample Size Re-estimation ... 56
10.2. Populations for Analyses ... 58
10.3. Statistical Analyses ... 58
10.3.1. Activity/Pharmacokinetic Analyses ... 58
10.3.2. Safety Analyses ... 60
10.3.3. Other Analyses ... 61
10.3.4. Efficacy Analyses ... 61

11. DOSE ESCALATION ... 61

12. REFERENCES ... 63

13. APPENDICES ... 65
13.1. Appendix 1: Abbreviations and Trademarks ... 65
13.2. Appendix 2: EBA definitions ... 67
13.3. Appendix 3: Clinical Laboratory Tests ... 68
13.4. Appendix 4: Study Governance Considerations ... 70
13.5. Appendix 5: Adverse Events: Definitions and Procedures for Recording, Evaluating, Follow-up, and Reporting ... 74
13.6. Appendix 6: Contraceptive Guidance and Collection of Pregnancy Information ... 80
13.7. Appendix 7: Liver Safety: Required Actions and Follow-up Assessments ... 83
13.8. Appendix 8: RADIATION DOSE ... 87
13.9. Appendix 9: Protocol Amendment History ... 88

1. SYNOPSIS

Protocol Title: A Phase IIa open-label trial to investigate the early bactericidal activity, safety and tolerability of GSK3036656 in participants with drug-sensitive pulmonary tuberculosis.

Short Title: GSK3036656 EBA Study

Objectives and Endpoints:

Objectives	Endpoints
**Primary**	
• To determine the early bactericidal activity of GSK3036656 over 14 days of once daily repeat dosing.	• Rate of change in log_{10}CFU per mL direct respiratory sputum samples over the period baseline to Day 14 (EBA $CFU_{0–14}$).
**Secondary**	
• To assess the safety and tolerability of GSK3036656 administered once daily over 14 days to participants with tuberculosis.	• Adverse events (AE), clinical laboratory values, vital signs, electrocardiogram (ECG) parameters.
• To characterise the pharmacokinetics of 14-day once daily dosing of GSK3036656 in tuberculosis participants.	• Derived pharmacokinetic parameters for GSK3036656 including area under the plasma drug concentration versus time curve ($AUC_{0–t}$, $AUC_{0–24}$), maximum observed plasma drug concentration (Cmax), and time to maximum observed plasma drug concentration (tmax), as appropriate.
• To determine the early bactericidal activity of GSK3036656 over the first 2 days and the last 12 days with 14 days of once daily repeat dosing.	• Rate of change in log_{10}CFU per mL direct respiratory sputum samples over the period baseline to Day 2 (EBA CFU_{0-2}).<br>• Rate of change in log_{10}CFU per mL direct respiratory sputum samples over the period Day 2 to Day 14 (EBA CFU_{2-14}).<br>• Rate of change in time to sputum culture positivity (TTP) over the time period baseline to Day 14 (EBA TTP_{0-14}).

Objectives	Endpoints
	• Rate of change in time to sputum culture positivity over the time period baseline to Day 2 (EBA $TTP_{0\text{-}2}$).<br>• Rate of change in time to sputum culture positivity over the time period Day 2 to Day 14 (EBA $TTP_{2\text{-}14}$).
**Exploratory**	
[redacted]	

For the purpose of assessment of bacterial [redacted] endpoints, the pre-treatment baseline will be defined as the mean of Day –2 and Day –1; if data is available at only Day –2 or Day –1 then that value will be used as baseline. For ECG endpoints, baseline is screening. [redacted]

Overall Design:

A single-centre, open-labelled, clinical trial in up to five sequential cohorts of participants with rifampicin-susceptible tuberculosis (DS-TB). Each cohort will involve participants being randomised to one of two possible treatments: either GSK3036656 or Standard-of-care (SoC) regimen for DS-TB (i.e. Rifafour e-275 or equivalent generic alternative). The ratio of participants assigned to GSK3036656 vs Rifafour e-275 (or equivalent generic alternative) will be 3:1.

The starting dose is set at 5 mg once daily (with a 15mg loading dose on day 1) given orally and the study will employ dose escalation, where the dose for each cohort will be decided based on reviews of preliminary safety, tolerability and pharmacokinetic data. This includes the possibility of subsequent doses being less than or the same as a preceding dose, if appropriate.

After the initial dose of GSK3036656, the decision to proceed to each subsequent dose level will be made by the Dose Escalation Committee (DEC) based on safety, tolerability and preliminary pharmacokinetic data from the prior cohort. The DEC may also include efficacy data in their deliberations, if that is available.

Number of Participants: up to approximately 100 participants will be enrolled.

Treatment Groups and Duration: Up to 5 cohorts; 12-20 participants in each cohort (9-15 participants will receive GSK3036656, and 3-5 participants will receive SoC in each cohort).

The approximate duration of the study for an individual participant is anticipated to be 5 weeks to include a maximum of 1 week for screening, 2 days of baseline sputum sample collection, 2 weeks for the treatment period and another 2 weeks for the final follow-up visit.

2. SCHEDULE OF ACTIVITIES (SOA)

**Period**	**Screening**			**Dosing**						**Follow-up**	
**Visit**	**1**	**2**	**3**	**4**	**5 to 10**	**11**	**12 to 16**	**17**	**18**	**Early Withdrawal**	**19**
**Day**	**(-9 to -3)** [A]	**-2**[B]	**-1**	**1**	**2 to 7**[C]	**8**	**9 to 13**[C]	**14**	**15**		**28 (± 7)**
Documentation of Positive GeneXpert and/or TB smear (TB clinic/site of initial diagnosis)	X										
Written Informed Consent	X										
Demography (including smoking history)	X										
Medical & Treatment History	X										
Inclusion/Exclusion/Eligibility Assessment		X									
Chest X-ray		X									
Physical Examination[D]	X			X	X	X	X	X	X	X	X
Vital Signs [E]	X	X	X	X	X	X	X	X	X	X	X
12-Lead ECG	X							X		X	X
Randomisation[M]			X								
Echocardiogram (may be conducted at any of the screening visits)		X								X[S]	X[S]
Blood PK[F]							X[F]	X	X	X	
Spot Sputum (confirm TB & adequate bacterial load) [G]	X										
Rifampicin Resistance Test (rapid)[G] (microscopy and GeneXpert tests may be repeated on an overnight sputum sample if needed)	X										
Haematology, Clinical Chemistry, Urinalysis [H]	X			X	X	X		X		X	X
Cardiac safety biomarkers[T]		X		X	X			X			
Urine Drug Screen[I]	X	X[I]									

Period	Screening			Dosing						Follow-up	
Visit	1	2	3	4	5 to 10	11	12 to 16	17	18	Early Withdrawal	19
Day	(-9 to -3) [A]	-2[B]	-1	1	2 to 7[C]	8	9 to 13[C]	14	15		28 (± 7)
HIV Test (and CD4 Count if HIV confirmed)	X										
[redacted]				X	X			X		X	
[redacted]				X	X			X		X	
[redacted]		X						X			
Hospital Admission	X[B]	X[B]									
Overnight Sputum[L]	X	X	X	X	X	X	X	X			
IP Administration and Compliance Check[N]				X	X	X	X	X			
Mycobacteriology Assessments [O]		X	X					X			
Hospital Discharge									X	X	
Concomitant Medication		X	X	X	X	X	X	X	X	X	X
Adverse Events[P]				X	X	X	X	X	X	X	X
Point of Care Blood Glucose or HbA1c[Q]		X						X			

A. The Visit 1 (day -9 to -3) time period will be up to a maximum of 7 days but will be kept as short as possible.

B. Participants can proceed with the Visit 2 (day -2) assessments as soon as their Visit 1 (day -9 to -3) assessments have been completed i.e. Visits 1 and 2 may occur on the same day as long as the screening results are available in time for randomisation. Participants may be hospitalised during the entire pre-treatment period if the Investigator considers it advisable for reasons of safety or compliance.

C. All events listed as occurring on Visit 5 (day 2) to Visit 10 (day 7) and Visit 12 (day 9) to Visit 16 (day 13) will be conducted each day during these visits.

D. Height (m) will only be collected once at Visit 1 (day -9 to -3). A full physical examination will be performed at Visit 1 (day -9 to -3), with symptom-directed physical examinations at Visit 17 (day 14), Visit 19 (day 28) and Early Withdrawal Visit (EWV). Symptom directed physical examinations may be conducted as required during the study (visits 4 to 16). Requirements for the physical examination are specified in the SRM.

E. Systolic blood pressure (SBP) and diastolic blood pressure (DBP) (mmHg), heart rate (beats per minute [bpm]), body temperature (°C), respiratory rate (breathes per minute) and weight (kg). To be performed within 4 hours prior to the first daily dosing on Visit 4 (day 1) through to Visit 17 (day 14) and within 4 hours before the time dosing would have occurred on Visit 18 (day 15). On days where the following assessments are done the order should be: vital signs before blood draws for PK or safety assessments.

F. PKs will be performed as noted in the PK table. A single pre-dose PK sample will be collected on each of Days 12 & 13, and serial PK blood sampling will occur on Day 14 (see PK table below). At EWV a PK sample will be taken before the time of the planned daily dosing. If this is not possible, a spontaneous PK sample will be taken.

G. Sputum smear microscopy and molecular rapid test for confirmation of Mycobacterium tuberculosis and rifampicin susceptibility (GeneXpert). If the first spot sputum or overnight sputum sample shows an unfavourable result, the tests may be repeated on a freshly collected spot sputum or overnight sputum sample and that result used instead.

H.). Refer to Appendix 3 for details.

I. Urine drug screen: cocaine, amphetamines and opiates. Urine drug screen can be repeated at Visit 2 (day -2) for participants who were not hospitalized at Visit 1 (day -9 to -3).

J. CCI [redacted]

K.

L. Overnight sputum sampling may start at Screening visit (day -9 to -3) or on Visit 2 (day –2) and will continue daily until Visit 7 (day 4). Additional sampling will be conducted at visit 9 (day 6), Visit 11 (day 8), Visit 13 (day 10), Visit 15 (day 12) and Visit 17 (day 14). Sputum sampling will stop on the morning of Visit 18 (day 15). Sputum collection will start in the afternoons at 15:00 hours (±1 hour) and continue for 16 hours overnight. The 16-hour sputum sampling for each of the sampling days must be finished prior to the administration of the next day's IP.

Overnight sputum collection may be collected as many times as required over the screening period until an eligible sample is obtained. Of the pre-treatment period samples collected, only the Visit 2 (day-2) and Visit 3(day -1) overnight sputum samples will be used for the efficacy endpoint tests.

The day sputum collection starts reflects the day to which that sample applies. e.g. a sample whose collection starts on day 1 and ends on day 2, is designated as the day 1 overnight sputum sample (and results).

M. Randomization may occur once all the screening results are available and the Investigator has determined that the participant is eligible for the trial.

N. After the first dose of IP, subsequent doses of IP will be administered 23 to 25 hours after the previous dose.

O. Minimum inhibitory concentration (MIC) and speciation of the infecting organism by polymerase chain reaction (PCR) will be estimated from a culture collected on a pre-treatment day (day-1 or day-2) and from the last available culture on a treatment day (day 14 or earlier, but not earlier than day 8). Drug susceptibility testing of *M. tuberculosis* for sensitivity to rifampicin with a molecular method will be tested at Visit 1. Susceptibility to INH, Rif, EMB and PZA will be tested on a culture grown from a baseline sample (day -2 or day -1). The analysis of any bacterial isolates sent to GSK, Spain for further characterisation in the event of MIC change will not be reported in the clinical study report and will be reported separately.

P. Adverse events (AE) will be collected by the Investigator from the time a participant receives his/her first dose of IP through to the Visit 19 (day 28) Follow up Visit.

Q. CCI [redacted]

R. *[not used]*

S. Only to be performed on participants as determined by the dose escalation committee (DEC) or on participants with changes in clinical status warranting a follow-up echo in the opinion of the investigator. The DEC may decide to perform this assessment in additional cohorts within this study.

T. Samples will be assayed for: Troponin I. The first two samples at least 24 hours apart, one during the screening phase and one prior to the first dose of study treatment. Day 14 sample window is ±24 hours. Third sample to be taken on Day 3.

PK sampling time and events table

STUDY VISIT	Visit 15	Visit 16	Visit 17										Visit 18
STUDY DAY	Day 12	Day 13	Day 14										Day 15
STUDY HOUR[B]			0	0.5	1	1.5	2	3	4	6	8	12	24
Pharmacokinetic sampling[C]	X[A]	X[A]	X[D]	X	X	X	X	X	X	X	X	X	X

A. Sample to be taken within 30 minutes pre-dose.

B. All values are relative to the time of day that the study treatment is administered on Day 14.

C. PK sampling is only to be performed on participants receiving GSK3036656. PK samples will not be taken from participants receiving Rifafour e-275 (or equivalent generic alternative).

D. Sample to be taken within 10 minutes pre-dose

PK Sample timing window allowances

Sample timepoint	Sample collection window allowed
Pre dose	Not applicable (see footnotes above)
0h - 4h post	± 5 min
> 4h – 12h post	± 15 min
> 12h – 24h post	± 1 h

3. INTRODUCTION

3.1. Study Rationale

This trial seeks to establish the antituberculosis effect of GSK3036656 on serial CFU counts of *Mycobacterium tuberculosis* (Mtb) in sputum over 14 days of therapy.

The assessment of the early bactericidal activity (EBA) of antituberculosis agents in sputum over the first 2 weeks of treatment is the established method for the early clinical evaluation of new antituberculosis agents and regimens [Jindani, 1980]. The primary endpoint of EBA studies is the daily rate of change in colony forming units (CFU) of Mtb in sputum (measured as log_{10}CFU/mL sputum/day) or the prolongation of time to positivity (TTP) in liquid culture (measured in hours/day) in participants with smear-positive pulmonary tuberculosis (TB). This approach potentially provides an indication whether a novel treatment has antituberculosis properties in humans and gives an early indication of the dosage required. Pharmacokinetic analyses offer the opportunity to study the relationship between dosages, serum drug levels, and efficacy. Preliminary safety and tolerability data can also be collected for the duration of the treatment [Donald, 2003; Diacon, 2011a].

Additional details related to rationale for the study design appears in Section 5.5.

3.2. Background

TB remains a concerning health problem, with Mtb now causing more deaths than HIV/AIDS. Six countries in Africa and Asia accounted for the majority of the 10.4 million new TB cases reported in 2015 (WHO, 2016a). The current long-standing first-line antituberculosis agents are relatively ineffective in controlling the TB epidemic in high-burden countries. Treatment takes 6-9 months to complete and is associated with side effects resulting in poor compliance which leads to treatment failure and an increased likelihood of developing drug-resistance.

Drug-resistant TB poses a major threat to control of the TB epidemic. The prevalence of multi drug-resistant TB (MDR: resistance to at least isoniazid and rifampicin) and extensively drug-resistant (XDR: MDR plus resistance to at least a fluoroquinolone and an injectable antituberculosis drug) continues to increase. In 2015, an estimated 580 000 new cases qualified for MDR-TB treatment and incident cases of XDR-TB, approximately 10% of patients with MDR-TB, were reported in more countries, including Eastern Europe and India (WHO, 2016a).

The treatment of DR-TB remains sub-optimal due to constraints such as availability of drugs, duration (9−24 months, including a daily injectable in the initial phase), and significant toxicities leading to poor compliance and an increased risk of developing further drug-resistance. The new 9-month regimen for MDR-TB recently proposed by the WHO is optimising the use of currently known substances but only a minority of MDR cases are likely to qualify for it (WHO, 2016b). Despite the lengthy duration, DR-TB treatment is only successful in around half of MDR- and less than a third of XDR-TB cases, further escalating the threat to global health.

The development of new drug combinations that are effective against drug-resistant strains of Mtb, and that have the potential to shorten the duration of TB treatments offers the hope of tackling the TB pandemic. The addition of promising new drugs such as moxifloxacin, bedaquiline [Rustomjee, 2008; Diacon, 2009; Diacon, 2013], delamanid [Diacon, 2011b], PA-824 [Diacon, 2010; Diacon, 2012], new oxazolidinone candidates [Wallis, 2014; Furin, 2016] for M/XDR-TB is an important step in that direction. However, questions remain related to their best possible use in combination regimens.

A new treatment regimen requires at least three different drugs to which no clinical resistance exists. However, potential antagonism in humans between drugs [Mdluli, 2009], for instance PA-824 and bedaquiline, and the additive potential for significant toxicity e.g. QTc prolongation of moxifloxacin [Bloomfield, 2008], bedaquiline [Matteelli, 2010], limit the number of possible combinations. This highlights the need for the development of additional new chemical entities that strike an appropriate balance between antituberculosis activity and safety profiles, a balance that will enable new future drug combinations that make the optimal use of previously-developed entities.

GSK3036656 is a compound with a novel mechanism of action under development for the treatment of TB. It suppresses protein synthesis in Mtb by selectively inhibiting the enzyme Leucyl t-RNA synthetase.

There is no known circulating resistance to GSK3036656, and GSK3036656 is active against both drug-sensitive and drug-resistant strains of Mtb when screened in a variety of i*n vitro* and *in vivo* animal models. The MIC is low (MIC90 of 0.16 μM against a panel of clinical isolates) and the drug shows potent activity in animal models, resulting in a very low predicted efficacious human dose for GSK3036656 (10 mg, Section 5.6). *In vitro* data show that the risk of drug-drug interactions with GSK3036656, either as perpetrator or victim, is low. These factors make GSK3036656 a very attractive candidate for the treatment of tuberculosis.

A description of the chemistry, nonclinical and clinical data generated to date for GSK3036656 is provided in the Investigator's Brochure (IB).

3.3. Benefit/Risk Assessment

Consistent with GlaxoSmithKline guidance for early phase studies, GSK3036656 will be administered in an in-patient setting (with sufficient overnight facilities) with appropriate monitoring.

Summaries of findings from non-clinical and first time in human (FTIH) clinical studies conducted with GSK3036656 can be found in the IB. The following section outlines the risk assessment and mitigation strategy for this protocol.

More detailed information about the known and expected benefits and risks and reasonably expected adverse events of GSK3036656 may be found in the IB.

3.3.1. Risk Assessment

Potential Risk of Clinical Significance	Summary of Data/Rationale for Risk	Mitigation Strategy
**Investigational Product (IP) GSK3036656**		
Reduction in red blood cell count	**Non-Clinical**<br><br>In a 4-week rat study (doses 3, 10 & 30 mg/kg/d) - dose-dependent decline in reticulocytes (by up to 52% in males and 62% in females) at day 7 followed by normal levels by day 14 and increased levels by day 29/30 compared to controls. Hgb levels decreased by 10-13% compared to controls on day 14 and day 29/30.<br><br>In a 4 week dog study (doses 5, 10 & 20 mg/kg/d) - mild dose-dependent decrease in Hgb on day 14 (by about 10% compared to controls) with recovery by week 4. Reticulocyte counts unaffected. Dogs not bled on day 7.<br><br>No histopathological changes in bone marrow or other relevant histopathology in the 4-week studies.<br><br>**Clinical**<br><br>In the 201040 study, a possible trend of decreased haemoglobin from baseline to follow-up was noted in aggregate haematology data. The mean change	Standard safety haematology and clinical chemistry assessments will be performed and both trends and changes outside normal range will be monitored as part of laboratory safety assessments.<br><br>Participants whose haemoglobin drops below pre-specified limits will be withdrawn from the study (Section 8.1.3).

Potential Risk of Clinical Significance	Summary of Data/Rationale for Risk	Mitigation Strategy
	from baseline to follow-up was -4.9 g/L in the 15 mg repeat dose cohort, compared to -1.5 g/L in the placebo group. None of the values hit potential clinical importance (PCI) criteria, and declines were not clinically relevant.<br><br>No clinically significant trends in reticulocyte values were observed, and there were no values of potential clinical importance reported for reticulocytes.	
GSK3036656 is cleared through the kidneys	The main route of clearance for GSK3036656 is renal and there is therefore a risk that participants with low creatinine clearance will accumulate GSK3036656 and exceed predefined exposure limits.	Participants with a creatinine clearance less than 75 mL/min have been excluded.
Reduction in white blood cell count	**Non-Clinical**<br><br>In the 4-week rat study, decrease of up to 37% in total leucocyte count on day 29/30 compared to controls.<br><br>In the 4-week dog study, no reported effects on white blood cells.<br><br>**Clinical**<br><br>No clinically significant trends in leukocyte or neutrophil count values were observed. Two subjects	White blood cell count will be monitored as part of laboratory safety assessments. Any clinically significant changes will be followed up until levels are not clinically significant.<br><br>Participants developing neutrophil counts <500/mm^3 (1.0x10^9/L) will be withdrawn from the study (Section 8.1.3).

Potential Risk of Clinical Significance	Summary of Data/Rationale for Risk	Mitigation Strategy
	showed PCI values. One subject had low neutrophil count (normal range 1.57-6.81 × 10^9/L) from screening (1.38) until follow-up (1.41), and also showed low leukocyte count (normal range 3.3-9.8 × 10^9/L) on Day 4 (2.5), Day 10 (2.6) and Day 14 (2.9) and was within normal range at follow-up. Given the low screening values, this observation was considered likely related to the subject's Black or African American race. A second subject had a single low neutrophil count at follow-up (1.48).	
Heart valvular and vascular pathology	**Non-Clinical**<br><br>In a 7-day dog dose range finding (DRF) study – minimal focal subendocardial hemorrhage on the atrial surface of the left atrioventricular valve observed in both animals at the top dose of 60 mg/kg/d.<br><br>In a 10-day dog investigative study at 65 mg/kg/d – vascular lesions (including minimal focal necrosis and inflammatory infiltrate) in 3 out of 8 dogs.<br><br>In the 4-week dog GLP study - no heart or valve changes were observed up to the top dose of 20	This pathology is not monitorable in humans.<br><br>To avoid risk to study participants, dosing will be limited to doses at which individual exposures do not exceed AUC0-24 and Cmax to 15µg.hr/mL and 1.5µg/mL respectively.<br><br>These exposures are 10-fold below the lowest NOAEL achieved in the 17 week studies (rat)<br>In addition, although the pathology is not monitorable in humans, echocardiograms will be performed for all participants at screening and, for subjects receiving the highest dose of GSK3036656 or whose clinical status warrants

Potential Risk of Clinical Significance	Summary of Data/Rationale for Risk	Mitigation Strategy
	mg/kg/day and there was no increase in inflammatory markers.<br><br>In the 17 week GLP dog study there were no heart or valve changes up to the top dose of 45mg/kg/day.<br><br>In the 17 week rat study there were no changes in cardiac pathology up to the top dose of 30mg/kg/day.<br><br>Valvular or vascular pathology were not observed in rats. This pathological change is not commonly seen in animals, and its mechanism and significance to humans are not clear. A correlation between the presence of hemodynamic changes and the presence of this pathology is suspected, but currently unproven.<br><br>**Clinical**<br><br>Echocardiograms were performed at screening and follow up in the 201040 FTIH study in order to exclude participants with pre-existing valve or other cardiac abnormalities from the study, and to detect the presence of abnormalities after completion of the study. No abnormalities were detected.<br><br>No SAEs, or AEs of cardiovascular nature were reported. There was no change from baseline in	it, also at follow-up (or early withdrawal). Cardiac troponin will be measured.

Potential Risk of Clinical Significance	Summary of Data/Rationale for Risk	Mitigation Strategy
	heart rate observed at any dose. No trends in blood pressure change were observed. Cardiac telemetry and echocardiogram-related interpretations were within normal range. There were no clinically significant ECG observations. There were no troponin I abnormalities in participants after receiving study drug.	
Hemodynamic changes	**Non-Clinical**<br><br>Hemodynamic changes in several studies at ≥100 mg/kg in rats and ≥20 mg/kg in dogs - increased heart rate (rats and dogs), increased (rats) or decreased (dogs) blood pressure.<br><br>**Clinical**<br><br>Data from the 201040 FTIH study showed no significant changes in vital signs assessments during the study, and no clinically significant changes in ECG or telemetry assessments including QTc and PR intervals.	Vital signs monitoring will be implemented in this study.

Potential Risk of Clinical Significance	Summary of Data/Rationale for Risk	Mitigation Strategy
CCI [redacted]		

CCI [redacted]

Development of bacterial resistance to GSK3036656.	Administration of GSK3036656 as a monotherapy could potentially lead to the development of resistance to the compound in *M. tuberculosis* during the course of the study.	The risk of development of resistance in the EBA monotherapy study is considered to be low based on in-vitro data with GSK3036656 and historical experience with EBA studies in general. Susceptibility testing on samples of *M. tuberculosis* from participants will be conducted and assessed as part of the study to monitor for the development of resistance to GSK3036656. In-vitro data indicates that resistance to GSK3036656 does not confer any clinically significant cross resistance to SoC antitubercular treatment
Skin depigmentation	**Non-Clinical**<br><br>In a 17 week dog toxicology study (doses 0, 6, 20 & 45 mg/kg /day): loss of pigmentation on the eyelids,	 <br>The duration of dosing in this study (14 days) is shorter than that used in the dog toxicology

Potential Risk of Clinical Significance	Summary of Data/Rationale for Risk	Mitigation Strategy
	nose or muzzle of all animals at 45 mg/kg/day group (week 17 $AUC_{0–t}$ mean 405 µg.h/mL) and most animals in the 20 mg/kg/day group (week 17 $AUC_{0–t}$ 176 µg.h/mL). PNL-2 (a melanocyte marker) immunohistochemistry staining demonstrated moderate cytoplasmic positivity in a population of cells in the basal region of the epidermis (interpreted to be melanocytes). The decreased pigmentation was therefore not associated with a loss of melanocytes in affected animals on immunohistochemistry. Depigmentation was not observed in the 0 or 6 mg/kg/day group (59.4 ug/h/mL). Depigmentation was not observed in the two previous 4 week dog toxicology studies up to 45 mg/kg/day (week 4 $AUC_{0-t.}$ 388 ug.h/mL)<br><br>**Clinical**<br><br>No adverse events were reported in the 201040 FTIH study that would be considered consistent with depigmentation.	study and the maximum exposure (AUC_{0-24}) has been capped at 15 ug.h/mL (4-fold margin to the mg/kg/day dose).<br><br>The physical examination of study participants will include visual examination of the skin.<br><br>Participants with vitiligo will be excluded from the study to avoid confounding skin assessment at baseline and follow-up physical examinations.
Teratogenicity of GSK3036656	**Non-clinical safety**<br><br>Preliminary results from the definitive rat embryo-fetal toxicology are as follows:<br><br>**60mg/kg/day:** Reductions in fetal viability and reduced fetal weights, with minimal reductions in	Women will be excluded from recruitment to this study.

Potential Risk of Clinical Significance	Summary of Data/Rationale for Risk	Mitigation Strategy
	body weight gains and malformations including ventricular septal defects (13 animals across 8 litters) and cleft palates (10 animals/6 litters).<br><br>**20mg/kg/day:** Reduced fetal weights and incidences of ventricular septal defects (2 animals across 2 litters) that were outside the historical control data in the absence of any maternal effects.	

3.3.2. Benefit Assessment

Mouse models predict efficacy at human equivalent exposures consistent with the upper doses being tested. Nevertheless, efficacy of GSK3036656 has not been demonstrated in humans and no benefit can be expected from receiving the compound over the 2-week course of the study, and as monotherapy. A significant proportion of people with TB fail treatment (~15% with drug-sensitive up to ~80% with XDR). New effective compounds with novel mechanisms of action are urgently needed for TB, and it is recognised as a priority area by the WHO. The results of this study will establish whether GSK3036656 has activity, tolerability and safety adequate to take into subsequent studies of longer duration with the intention of developing a medicine for TB with a novel mechanism of action that could form part of a new combination treatment regimen for TB.

A delay of treatment with the full standard regimen of 14 days is not expected to have an adverse impact on the ultimate cure of TB in these participants, and this approach has been used as part of recent successful TB drug development programs (Diacon, 2011b, Diacon, 2013). Participants in this study will have their TB infection carefully characterized and drug sensitivity profiles established.

Participants will remain under constant medical attention and will be housed and monitored in hospital from admission through the duration of the treatment period; this will allow the continuous monitoring of the health conditions of each participant, any of whom can be withdrawn at any stage of the trial and removed from study treatment should his/her condition suggest to the Investigator that this would be in his/her best interest. Multiple blood samples will be taken for safety laboratory studies. Upon discharge, the participants will be given the initial doses of standard TB treatment and immediately referred to the national TB treatment program local TB clinic where they will receive a full course of standard treatment. At the follow-up visit it will be confirmed that this treatment has been initiated and is being continued. The Investigators' primary responsibility is to ensure participant safety.

3.3.3. Overall Benefit:Risk Conclusion

The overall benefit-risk balance is considered to be favourable in light of the safety data of GSK3036656 from the FTiH study, the mitigation strategies used and the potential benefit to study participants.

4. OBJECTIVES AND ENDPOINTS

Objectives	Endpoints
**Primary**<br>• To determine the early bactericidal activity of GSK3036656 over 14 days of once daily repeat dosing.	• Rate of change in log_{10}CFU per mL direct respiratory sputum samples over the period baseline to Day 14 (EBA CFU_{0-14}).
**Secondary**<br>• To assess the safety & tolerability of GSK3036656 administered once daily over 14 days to participants with tuberculosis.	• Adverse events (AE), clinical laboratory values, vital signs, electrocardiogram (ECG) parameters.
• To characterise the pharmacokinetics of 14-day once daily dosing of GSK3036656 in tuberculosis participants.	• Derived pharmacokinetic parameters for GSK3036656 including area under the plasma drug concentration versus time curve (AUC_{0-t}, AUC_{0-24}), maximum observed plasma drug concentration (Cmax), and time to maximum observed plasma drug concentration (tmax), as appropriate.
• To determine the early bactericidal activity of GSK3036656 over the first 2 days and the last 12 days with 14 days of once daily repeat dosing.	• Rate of change in log_{10}CFU per mL direct respiratory sputum samples over the period baseline to Day 2 (EBA CFU_{0-2}).<br>• Rate of change in log_{10}CFU per mL direct respiratory sputum samples over the period Day 2 to Day 14 (EBA CFU_{2-14}).<br>• Rate of change in time to sputum culture positivity (EBA TTP) over the time period baseline to Day 14 (EBA TTP_{0-14}).<br>• Rate of change in time to sputum culture positivity over the time period baseline to Day 2 (EBA TTP_{0-2}).<br>• Rate of change in time to sputum culture positivity over the time period Day 2 to Day 14 (EBA TTP_{2-14}).

Objectives	Endpoints
**Exploratory**	
[redacted]	

For the purpose of assessment of bacterial [redacted] endpoints, the pre-treatment baseline will be defined as the mean of Day –2 and Day –1; if data is available at only Day –2 or Day –1, then that value will be used as baseline. For ECG endpoints, baseline is screening. [redacted]

5. STUDY DESIGN

5.1. Overall Design

The study will comprise up to 5 cohorts of participants. It is intended that 9-15 participants in each cohort (dependent on the cohort) will receive GSK3036656 and 3-5 will receive standard-of-care regimen for DS-TB (i.e. Rifafour e-275 or equivalent generic alternative), with a randomisation ratio of 3:1. There will be no placebo in this study. The study will be randomized, but not blinded (i.e., it will be 'open-label') with neither participant nor Investigator blinded to treatment. Study participants will revert to the standard treatment for TB (i.e. Rifafour e-275 or equivalent generic alternative) once the study treatment (Day 1 to Day 14) has been completed. All laboratory staff involved in analysing and reporting the primary and secondary change in log_{10}CFU counts and TTP endpoints results will be blinded to treatment allocation. Cohorts will proceed sequentially starting A through E, with preliminary safety, tolerability and pharmacokinetic data from the previous cohort reviewed prior to selecting the dose for the next cohort.

It is anticipated that it may be necessary to administer a dose of GSK3036656 the same or lower than in a previous cohort, dependent on preliminary safety, tolerability, pharmacokinetic or efficacy data.

[redacted]

CCI
[redacted]

5.2. Study Schematic

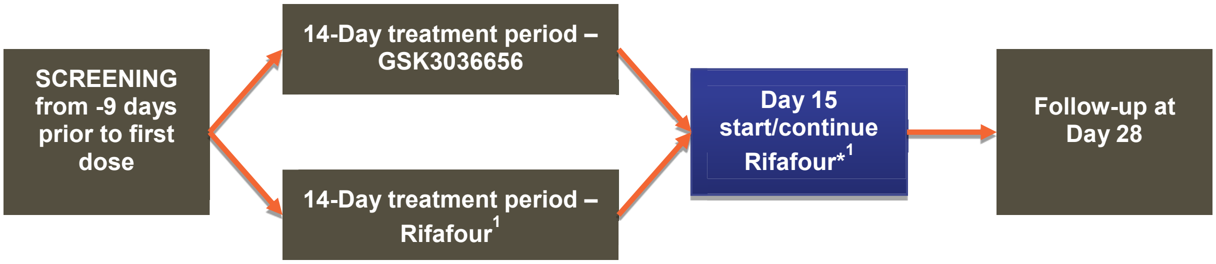

*This is not part of the study but is the post-study definitive treatment of TB.
[1]Rifafour e-275 or equivalent generic alternative.

5.3. Number of Participants

It is intended that 12- 20 participants will be enrolled into each cohort, dependent on the cohort.

If participants prematurely discontinue the study, or if their respiratory sputum cultures are contaminated, additional replacement participants may be recruited at the discretion of the Sponsor in consultation with the Investigator.

5.4. Participant and Study Completion

A participant is considered to have completed the study if he/she has completed all phases of the study including the last visit (the 'follow-up' visit). A participant is considered to have completed the study treatment phase once they have received the final dose of study treatment on Day 14.

The end of the study is defined as the date of the last visit of the last participant in the study.

Following completion of the study treatment phase and after discharge from hospital the participants will be provided with their first dose/s of standard TB therapy (i.e. Rifafour

e-275 or equivalent generic alternative) with appropriate counselling. All participants will be referred to the local community TB clinics to receive the remainder of a full course of standard antituberculosis chemotherapy according to national guidelines. The participants will be provided with a referral letter to take with them to the TB clinic. Within a week of the participant's discharge from the study clinic, a follow-up call will be made by the study site staff to the TB clinic to determine if the participant attended the clinic.

5.5. Scientific Rationale for Study Design

EBA studies are currently the only available way of assessing drug activity against TB early in drug development. EBA is a surrogate efficacy endpoint (decline of sputum bacterial counts) which allows the estimation of early drug effects in studies that are significantly shorter than studies using definitive clinical endpoints (e.g., relapse-free cure). [redacted]

EBA has a number of well-recognised limitations [Burman, 2003], and alternative surrogate endpoints are urgently needed. Studies evaluating the Mtb response to therapy in DS-TB subjects by [redacted] have already been reported, though at later timepoints, and a further study reporting the day 14 response has recently completed recruitment. [redacted]

Dose ranging is necessary to investigate the relationship between dose/exposure and efficacy, and to select an optimal dose for further progression into the clinic. Due to findings of cardiovascular toxicity in dogs, it is recommended that systemic exposures to GSK3036656 in humans do not exceed certain predefined limits (see Section 5.6). The PK of GSK3036656 has only been studied in healthy volunteers and its PK in TB participants is unknown. Therefore, to minimize the risk of exceeding the predefined exposure limits, a dose escalation approach will be used where the starting dose in the study is lower than doses previously administered safely to healthy volunteers with subsequent selection of doses based on PK and safety results.

The Rifafour e-275 (or equivalent generic alternative) treatment group is included as a benchmark for the EBA quantitative mycobacteriology and to evaluate whether HRZE in this population gives similar EBA results to that demonstrated in prior studies with this combination. A negative control arm, where subjects are left untreated, would be unethical due to the availability of effective treatment and the consequences of not treating TB.

5.6. Dose Justification

A population pharmacokinetic model was developed based on preliminary concentration-time data obtained in the FTIH study. The model was used to simulate loading and maintenance doses for GSK3036656 based on the EBA study design and accounting for

the effect of renal impairment on exposure (preliminary data suggest GSK303656 is > 70% renally excreted). A target exposure for efficacy of 1.74 ug.h/mL was set based on the exposure associated with efficacy in a mouse infection model and the target attainment rate was defined as the percentage of participants with an AUC_{0-24} greater than this target exposure. Exposure limits of AUC_{0-24} of 15 µg.h/mL and Cmax of 1.5 ug/mL were set, based on information in Section 3.3.

A loading dose of GSK3036656 may be administered on the first day (or over multiple days where required in order to not exceed AUC and Cmax exposure limits) of study treatment administration in each cohort. The long half-life of GSK3036656 (45 hours) means that subjects will take up to 10 days to reach steady-state concentrations, which is not optimal for a 14-day treatment study. A loading dose will allow participants to reach steady-state concentrations in 2 to 3 days for a given maintenance dose regimen.

The probability rates for individual participants to exceed GSK3036656 exposure limits and achieve the efficacy target for proposed loading and maintenance dose regimens are shown in Table 1.

Table 1 Probability rates of individual participants for proposed loading and maintenance doses

Loading Dose (mg)[a]	Probability Day 1 [c]	Maintenance Dose (mg)[b]	probability Day 14[d]	Target Attainment Rate[e]
15	<1%	5	<1%	<1%
25	<1%	10	<1%	100%
50	<1%	20	<1%	100%
75	6.90%	30	5.80%	100%
[a] Day 1				
[b] Day 2-14				
[c]per trial probability of an individual participant exceeding the exposure limits [AUC_{0-24} = 15 ug.h/mL or Cmax = 1.5 ug/mL] on Day 1 (simulations based on a lower limit of 75 mL/min for creatinine clearance)				
[d] per trial probability of an individual participant exceeding the exposure limits [AUC_{0-24} = 15 ug.h/mL or Cmax = 1.5 ug/mL] on Day 14 (simulations based on a lower limit of 75 mL/min for creatinine clearance)				
[e] per trial probability of >90% of participants exceeding the target exposure for efficacy				

The starting dose regimen for this study will be 15 mg loading dose on Day 1 and 5 mg maintenance dose on Day 2-14, based on the above information, with the option to

investigate a lower dose in subsequent cohorts should the preliminary data indicate this is appropriate.

The loading dose (if applicable) and maintenance dose for each cohort will be chosen on the basis that it is not predicted to exceed the pre-specified safety cap, and the dose used will be decided by the Dose Escalation Committee (Section 11).

6. STUDY POPULATION

Prospective approval of protocol deviations to recruitment and enrolment criteria, also known as protocol waivers or exemptions, is not permitted.

6.1. Inclusion Criteria

Participants are eligible to be included in the study only if all the following criteria apply:

Age

1. Participant must be 18 to 65 years of age inclusive, at the time of signing the informed consent.
2. CCI [redacted]

Type of Participant and Disease Characteristics

3. New episode of untreated, rifampicin-susceptible pulmonary TB.
4. A chest X-ray picture which in the opinion of the Investigator is consistent with TB.
5. At least one sputum sample positive on direct microscopy for acid-fast bacilli (at least 1+ on the IUATLD/WHO scale) or molecular test (Xpert MTB/Rif) with result of either medium or high positive for MTB
6. Ability to produce an adequate volume of sputum as estimated from an overnight sputum collection sample (estimated 10 mL or more). (If less than 10 mL is collected overnight this may be repeated once).
7. Normal echocardiogram or echocardiogram with normal left ventricular function with at most trace to mild valvular regurgitation is allowed and no valvular stenosis.
8. Within the normal range for the assay for troponin at screening.
9. A creatinine clearance ≥75 mL/min (Cockroft-Gault formula).

Weight

10. Body weight (in light clothing and with no shoes) between 40 and 90 kg, inclusive, at screening.

Sex

11. Male
    a. **Male participants:**

A male participant with female partners of child-bearing potential must agree to use contraception as detailed in Section 13.6 (Appendix 6).

Informed Consent

12. Capable of giving signed informed consent as described in Appendix 3 which includes compliance with the requirements and restrictions listed in the informed consent form (ICF) and in this protocol.

6.2. Exclusion Criteria

Participants are excluded from the study if any of the following criteria apply:

1. Evidence of a clinically significant (as judged by the Investigator) condition or abnormality (other than the indication being studied) that might compromise safety or the interpretation of trial efficacy or safety endpoints.
2. Poor general condition where any delay in treatment cannot be tolerated per discretion of the Investigator.
3. Clinically significant evidence of extrathoracic TB (miliary TB, abdominal TB, urogenital TB, osteoarthritic TB, TB meningitis), as judged by the Investigator.
4. QTcF > 450 msec.
5. History of allergy to any of the trial IP/s or related substances as confirmed by the clinical judgement of the Investigator.
6. Participants with vitiligo.
7. Current alcohol or drug abuse, that is, in the opinion of the Investigator, sufficient to compromise the safety or cooperation of the participant.
8. HIV infected participants:
    a. having a CD4+ count <350 cells/µL;
    b. or having received antiretroviral therapy medication within the last 30 days;
    c. or having received oral or intravenous antifungal medication within the last 30 days;
    d. or with an AIDS-defining opportunistic infection or malignancies [CDC, 1993] in the last 12 months (except pulmonary TB).
9. Participated in other clinical studies with investigational agents within 8 weeks prior to the first dosing day in the current study.
10. Participants with diabetes (Type 1 or 2), point of care HbA1c above 6.5 %, or random glucose over 11.1 mmol/L will be excluded from cohorts undergoing [redacted]

11. Treatment received with any drug active against Mtb (including but not limited to isoniazid, ethambutol, fluoroquinolones, rifampicin), or with immunosuppressive medications such as TNF-alpha inhibitors or systemic or inhaled corticosteroids (used for > 5 consecutive days), within 2 weeks prior to screening. Refer to the SRM for details.

12. Unavoidable treatment with prohibited concomitant medications (see SRM)

13. Participants with the following abnormal laboratory values at screening as graded by the enhanced Common Terminology Criteria for Adverse Events (CTCAE v 5 2017):

    a. haemoglobin <10.0 g/dL; this criterion may be adjusted following DEC review (see Section 11).

    b. thrombocytopenia grade 2 or greater (under 50×10^9 cells/L);

    c. serum potassium grade 2 or greater (<3.0 mEq/L);

    d. aspartate aminotransferase (AST) grade 3 (≥3.0 × ULN);

    e. alanine aminotransferase (ALT) grade 3 (≥3.0 × ULN);

    f. APTT grade 3 (≥2.5 × ULN)

    g. INR grade 3 (≥2.5 × ULN)

    h. Total white cell count grade 3 (<2.0×10^9 cells/L)

14. CCI [redacted]

6.3. Lifestyle Restrictions

6.3.1. Meals and Dietary Restrictions

- Refrain from consumption of red wine, Seville oranges, grapefruit or grapefruit juice, from the day of start of study treatment until after the final dose.
- Water is allowed ad libitum.

6.3.2. Caffeine, Alcohol, and Tobacco

- During each dosing session, participants will abstain from ingesting caffeine- or xanthine-containing products (e.g., coffee, tea, cola drinks, and chocolate) for 24 hours before the start of dosing until after collection of the final pharmacokinetic (PK) and/or pharmacodynamic sample.

- During each dosing session, participants will abstain from alcohol for 24 hours before the start of dosing until after collection of the final PK and/or pharmacodynamic sample.
- Use of tobacco products will be strongly discouraged from screening until after the final follow-up visit.

6.3.3. Activity

- Participants will abstain from strenuous exercise for 48 hours before each blood collection for clinical laboratory tests. Participants may participate in light recreational activities during the study (e.g., watching television, reading).

6.4. Screen Failures

Screen failures are defined as participants who consent to participate in the clinical study but are not subsequently randomised. A minimal set of screen failure information is required to ensure transparent reporting of screen failure participants to meet the Consolidated Standards of Reporting Trials (CONSORT) publishing requirements and to respond to queries from regulatory authorities. Minimal information includes demography, screen failure details, eligibility criteria, and any serious adverse events (SAEs).

7. TREATMENTS

Study treatment is defined as any investigational treatment(s), marketed product(s), placebo, or medical device(s) intended to be administered to a study participant according to the study protocol.

7.1. Treatments Administered

**Study Treatment Name:**	GSK3036656	Rifafour e-275 (or equivalent generic alternative)
**Dosage formulation:**	Capsule	Tablet
**Unit dose strength(s)/Dosage level(s):**	1 mg or 5 mg or 25 mg GSK3036656 capsules<br>(as free base equivalent)	Standard dose: 150/75/400/275 mg of Rifampicin, Isoniazid, Pyrazinamide and Ethambutol respectively per tablet
**Route of Administration**	Oral	Oral
**Dosing instructions:**	Refer to study reference manual	Refer to South African National TB Treatment Guidelines (including pyridoxine as required)
**Packaging and Labelling**	Study Treatment will be provided in HDPE bottles. Each HDPE bottle will be labelled as required per country requirement.	Will be provided by the manufacturer.
**Manufacturer**	WuXi AppTec (1 and 5 mg capsules)<br>Aptuit (25 mg capsule)	**Rifafour e-275:** Sanofi-Aventis or appropriate alternative<br>**Equivalent Generic alternative to Rifafour e-275:** Appropriate suppliers

Rifafour e-275 (or equivalent generic alternative).

Some participants will be treated with standard, intensive phase pulmonary TB treatment as recommended in the SA National TB Treatment Guidelines to verify that the laboratory assays used measure the expected magnitude of activity. This is HRZE (Rifafour e-275 or equivalent generic alternative) (HRZE: H=isoniazid: R=rifampicin: Z=pyrazinamide: E=ethambutol). The Rifafour e-275 (or equivalent generic alternative) treatment group is included as a benchmark for the EBA quantitative mycobacteriology and to evaluate whether HRZE in this population gives similar EBA results to that demonstrated in prior studies using this combination.

Rifafour e-275 (or equivalent generic alternative) Package Insert contains its known and potential risks and benefits.

7.2. Method of Treatment Assignment

On or prior to day 1, eligible participants will be assigned a unique number (randomization number) in ascending numerical order at the study site. The randomization number encodes the participant's assignment to one of the two arms of the study (GSK3036656 or standard-of-care), according to the randomization schedule generated prior to the study by the Statistics Department at GSK, using validated internal software. Once a participant number has been assigned to a subject, it cannot be re-assigned to another subject.

7.3. Preparation/Handling/Storage/Accountability

1. The Investigator or designee must confirm appropriate temperature conditions have been maintained during transit for all study treatment received and any discrepancies are reported and resolved before use of the study treatment.
2. Only participants enrolled in the study may receive study treatment and only authorized site staff may supply or administer study treatment. All study treatments must be stored in a secure, environmentally controlled, and monitored (manual or automated) area in accordance with the labeled storage conditions with access limited to the Investigator and authorized site staff.
3. The Investigator, institution, or the head of the medical institution (where applicable) is responsible for study treatment accountability, reconciliation, and record maintenance (i.e., receipt, reconciliation, and final disposition records).
4. Further guidance and information for the final disposition of unused study treatment are provided in the Study Reference Manual.

- Under normal conditions of handling and administration, study treatment is not expected to pose significant safety risks to site staff.

Precaution will be taken to avoid direct contact with the study treatment. A Material Safety Data Sheet (MSDS) describing occupational hazards and recommended handling precautions will be provided to the Investigator. In the case of unintentional occupational exposure notify the monitor, Medical Monitor and/or GSK study contact.

7.4. Treatment Compliance

- Individual doses for a participant will be prepared from a bulk supply, the preparation of the dose will be confirmed by a second member of the study site staff.
- Participants are dosed at the site. They will receive study treatment directly from the Investigator or designee, under medical supervision. The date and time of each dose administered in the clinic will be recorded in the source documents. The dose of study treatment and study participant identification will be confirmed at the time of dosing by a member of the study site staff other than the person administering the study treatment. Study site personnel will examine each participant's mouth to ensure that the study treatment was ingested.

7.5. Concomitant Therapy

Any medication or vaccine (including over-the-counter or prescription medicines, vitamins, and/or herbal supplements) that the participant is receiving at the time of enrolment or receives during the study must be recorded along with:

- reason for use
- dates of administration including start and end dates
- dosage information including dose and frequency

The Medical Monitor should be contacted if there are any questions regarding concomitant or prior therapy.

Participants must abstain from taking prescription or non-prescription drugs (including vitamins and dietary or herbal supplements) within 7 days (or 14 days if the drug is a potential enzyme inducer) or 5 half-lives (whichever is longer) before the start of study treatment until completion of the follow-up visit, unless, in the opinion of the Investigator and Sponsor, the medication will not interfere with the study. A list of prohibited and permitted concomitant medication is provided in the SRM.

8. DISCONTINUATION CRITERIA

If study treatment/investigational product is interrupted or stopped this does not automatically result in the participant being withdrawn from the study. The investigator will make all effort to retain the patient in the study, provided the participant continues to provide their consent, and to complete and record all scheduled safety assessments required by the protocol. For the avoidance of all doubt: this does not prejudice the rights of the investigator and study participants under ICH GCP and under applicable laws. The study participant retains the right to withdraw consent at any time for any reason. The investigator also retains the right to withdraw the patients from the study if in the investigator's judgement, withdrawal is judged clinically necessary to protect a participant's safety.

If this results in the patient being placed on standard of care (i.e. Rifafour e-275 or equivalent generic alternative), then all assessments relevant to Rifafour e-275 (or equivalent generic alternative) will be performed as directed by the protocol (for

example, GSK3036656 pharmacokinetic assessments are not required, since the participant is not being dosed with GSK3036656).

8.1. Discontinuation of Study Treatment

8.1.1. Liver Chemistry Stopping Criteria

Liver chemistry stopping and increased monitoring criteria have been designed to assure participant safety and evaluate liver event etiology. An algorithm for liver chemistry stopping and increased monitoring criteria is provided below. In addition, further information is contained in Section 13.7.

Discontinuation of study treatment for abnormal liver tests is also required when:

- in the presence of abnormal liver chemistries not meeting protocol-specified stopping rules, the Investigator believes study treatment discontinuation is in the best interest of the participant.

Phase II Liver Chemistry Stopping and Increased Monitoring Algorithm

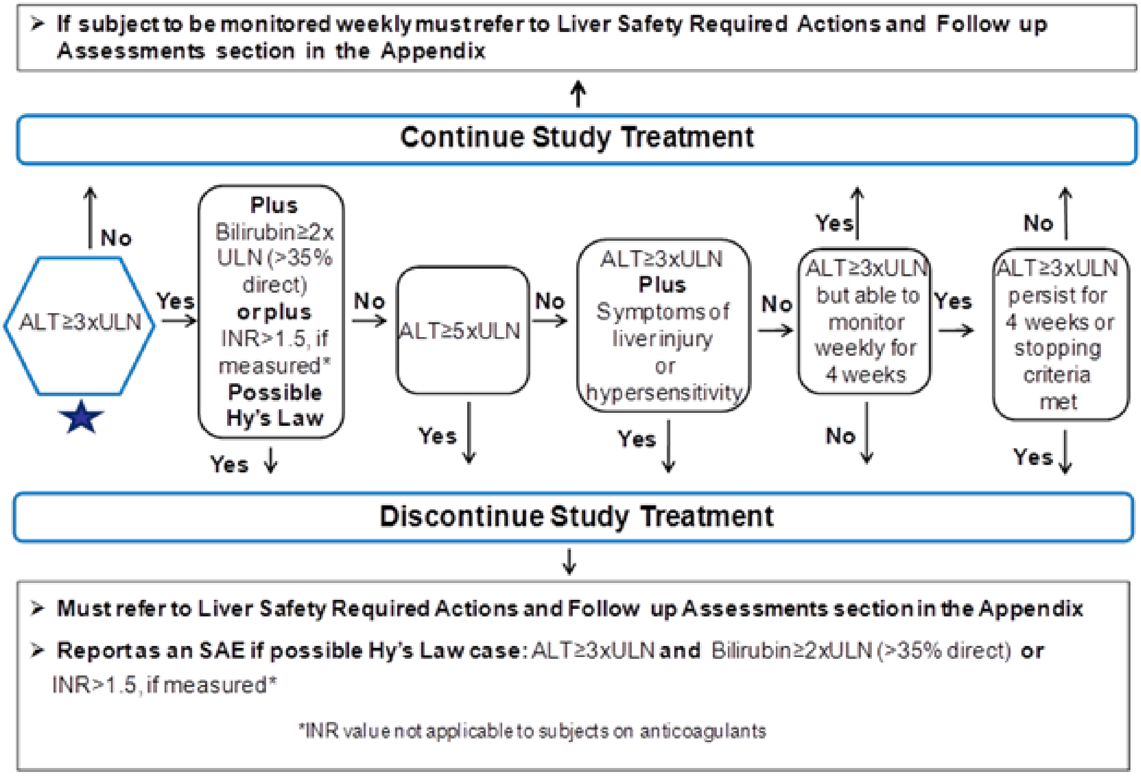

8.1.2. QTc Stopping Criteria

A subject that meets either bulleted criterion below will be withdrawn from the study.

- QTcF >500 msec,
- Change from baseline: increase in QTcF >60 msec

Withdrawal of participants is to be based on an average QTcF value of triplicate ECGs. If an ECG demonstrates a prolonged QT interval, obtain two more ECGs over a brief period of time (less than 10 minutes) and then use the averaged QTcF values of the three ECGs to determine whether the subject should be discontinued from the study.

8.1.3. Haematology Stopping Criteria

IP will be discontinued for a subject if the following haematology stopping criteria are met (confirmed on two samples or at the discretion of the investigator in consultation with the medical monitor) and participants should be followed up until values are not clinically significant.

- A reduction in haemoglobin to below 8.0 g/dL; this criterion may be adjusted following DEC review (see Section 11).
- A reduction in neutrophil count to below 500/mm^3 (0.5 × 10^9/L)

8.2. Individual/Cohort Stopping Criteria

For an individual study participant, stopping criteria include, but are not limited to:

- Severe signs or symptoms, or significant changes in any of the safety assessments that put the safety of the individual at risk (e.g. ECG, vital signs, laboratory tests etc.), as judged by the Investigator, in consultation with the Medical Monitor if necessary.
- Any unacceptable adverse event that is thought to be related to the investigational product and any general safety finding that, in the opinion of the Investigator, gives cause for concern may result in the withdrawal of subject(s) and/or the study being paused or terminated and/or a dose adjustment.

Dose escalation will be stopped if any of the following occurs in a cohort:

1. There is substantially increased incidence and/or severity of clinically significant adverse events or withdrawals possibly related to GSK3036656 in the GSK3036656 treatment subgroup at the last dose level administered as compared to the SoC subgroup. The Dose Escalation Committee in conjunction with the study Investigator will determine the significance of the adverse events or withdrawals and make a decision about whether stopping further dose escalation is necessary.
2. At least one subject has a plasma $AUC_{0–24}$ or Cmax higher than the exposure limits set in Section 5.6.

If dose escalation is stopped due to reasons 1 or 2 above occurring at the last dose, dosing at a lower level could be allowed by the Dose Escalation Committee. The Dose

Escalation Committee will decide whether dosing at such level could be done and will select the dose to be administered.

Further details regarding dose escalation and the Dose Escalation Committee composition and remit are in Section 11.

8.3. Trial Stopping Criteria

The trial will be stopped if either of the following occurs:

- A serious adverse event that is considered to be at least possibly related to GSK3036656 and is not commonly seen in participants with TB in one or more participants on active treatment.
- Severe, clinically significant non-serious adverse events that are considered by the investigator and the GSK Medical Monitor to be at least possibly related to GSK3036656 and are not commonly seen in participants with TB in 2 or more participants on active treatment in the same cohort.

If, following an internal safety review, it is appropriate to restart the trial with the protocol unchanged, the South African Health Products Regulatory Authority (SAHPRA) and Independent Ethics Committee (IEC) will be informed. If an amendment to the protocol is required, the trial will not restart until the new amendment has been approved by the regulatory agency and Independent Ethics Committee (IEC).

The Dose Escalation Committee (DEC) is empowered to stop the study within the remit specified in Section 11.

8.4. Withdrawal from the Study

- A participant may withdraw from the study at any time at his/her own request, or may be withdrawn at any time at the discretion of the Investigator for safety, behavioural, compliance or administrative reasons.
- If the participant withdraws consent for disclosure of future information, the Sponsor may retain and continue to use any data collected before the withdrawal of consent.
- If a participant withdraws from the study, he/she may request destruction of any samples taken and not tested, and the Investigator must document this in the site study records.
- Refer to the SoA for data to be collected at the time of follow-up and for any further evaluations that need to be completed.

8.5. Lost to Follow Up

A participant will be considered lost to follow-up if he or she repeatedly fails to return for scheduled visits and is unable to be contacted by the study site.

The following actions must be taken if a participant fails to return to the clinic for a required study visit:

- The site must attempt to contact the participant and reschedule the missed visit as soon as possible and counsel the participant on the importance of maintaining the assigned visit schedule and ascertain whether or not the participant wishes to and/or should continue in the study.
- Before a participant is deemed lost to follow up, the Investigator or designee must make every effort to regain contact with the participant (where possible, 3 telephone calls or local equivalent methods). These contact attempts should be documented in the participant's medical record.
- Should the participant continue to be unreachable, he/she will be considered to have withdrawn from the study with a primary reason of lost to follow-up.

9. STUDY ASSESSMENTS AND PROCEDURES

- Study procedures and their timing are summarized in the SoA.
- The timing and number of planned study assessments, including safety, pharmacokinetic, pharmacodynamic/biomarker assessments may be altered during the course of the study based on newly available data (e.g., to obtain data closer to the time of peak plasma concentrations) to ensure appropriate monitoring.
- Any changes in the timing or addition of time points for any planned study assessments must be documented and approved by the relevant study team member and then archived in the Sponsor and site study files, but will not constitute a protocol amendment. The IRB/IEC will be informed of any safety issues that require alteration of the safety monitoring scheme or amendment of the ICF.
- Protocol waivers or exemptions are not allowed.
- Immediate safety concerns must be discussed with the Sponsor immediately upon occurrence or awareness to determine if the participant should continue or discontinue study treatment.
- Adherence to the study design requirements, including those specified in the SoA, is essential and required for study conduct.
- All screening evaluations must be completed and reviewed to confirm that potential participants meet all eligibility criteria. The Investigator will maintain a screening log to record details of all participants screened and to confirm eligibility or record reasons for screening failure, as applicable.
- Procedures conducted as part of the participant's routine clinical management (e.g., blood count) and obtained before signing of ICF may be utilized for screening or baseline purposes provided the procedure met the protocol-specified criteria and was performed within the time frame defined in the SoA.

9.1. Microbiological Efficacy/Activity Assessments

The EBA CFU_{0-14} as determined by the rate of change in $\log_{10}$CFU per mL sputum over the period baseline to Day 14 which will be summarised and described with a statistical model as an estimated average decrease per day for participants in each group.

The EBA TTP_{0-14} as determined by the percentage rate of change in TTP per mL sputum over the period baseline to Day 14, which will be summarised and described with a statistical model as an estimated average increase per day for participants in each group.

Drug activity with CFU or TTP over different treatment periods such as baseline - Day2, and Day2-14, for cross-comparison with other published data. Data will be displayed graphically, together with modelled estimates.

For the purposes of the microbiological endpoints assessments, the pre-treatment baseline will be defined as the mean of Day -2 and Day -1; if data is available at only one of these timepoints then that value will be used as baseline, and data prior to Day −2 will not be used to calculate the mean at baseline.

Mycobacterial Characterisation:

The identifying organism will be confirmed as Mtb at least once by polymerase chain reaction (PCR) at screening (GeneXpert or Line Probe) and a sample collected before treatment will be submitted for culture confirmation.

Drug susceptibility testing of Mtb for sensitivity to rifampicin with a molecular method will be tested at the screening visit (GeneXpert) to determine eligibility. Susceptibility to RIF, INH, PZA and EMB will be confirmed on a culture grown from a sample collected before treatment either by phenotypical testing or line probe.

Cultures from baseline and from day 14 sputum will be kept for determination of the minimum inhibitory concentration (MIC) of the investigational agents that the subject was treated with. If a day 14 culture is not available, the last available culture after day 8 will be kept. In the event of significant changes in the MIC, bacterial isolates will be sent to the GSK Spain laboratory for further characterisation. Technical details are provided in the SRM. MIC data reporting will likely occur after the clinical database has been authorised for the primary and secondary efficacy and safety endpoints at the end of the study (i.e. after Database Freeze [DBF]). The report detailing the data will remain as a separate report and will not be included in the GSK clinical study report.

9.2. Adverse Events

The definitions of an AE or SAE can be found in Appendix 5. The Common Terminology Criteria for Adverse Events (CTCAE) v5 2017 will be used to define AE grade and severity.

The Investigator and any designees are responsible for detecting, documenting, and reporting events that meet the definition of an AE or SAE and remain responsible for following up AEs that are serious, considered related to the study treatment or the study, or that caused the participant to discontinue GSK3036656 (see Section 8).

9.2.1. Time Period and Frequency for Collecting AE and SAE Information

- All SAEs will be collected from the start of treatment until the follow-up visit at the time points specified in the SoA (Section 2). However, any SAEs assessed as related to study participation (e.g., study treatment, protocol-mandated procedures, invasive tests, or change in existing therapy) or related to a GSK product will be recorded from the time a participant consents to participate in the study.
- All AEs will be collected from the start of treatment until the follow-up visit at the time points specified in the SoA (Section 2).
- Medical occurrences that begin before the start of study treatment but after obtaining informed consent will be recorded on the Medical History/Current Medical Conditions section of the case report form (CRF) not the AE section.
- All SAEs will be recorded and reported to the Sponsor or designee immediately and under no circumstance must this exceed 24 hours, as indicated in Appendix 5. The Investigator will submit any updated SAE data to the Sponsor within 24 hours of it being available.
- Investigators are not obligated to actively seek AEs or SAEs in former study participants. However, if the Investigator learns of any SAE, including a death, at any time after a participant has been discharged from the study, and he/she considers the event to be reasonably related to the study treatment or study participation, the Investigator must promptly notify the Sponsor.
- The method of recording, evaluating, and assessing causality of AEs and SAEs and the procedures for completing and transmitting SAE reports are provided in Appendix 5.

9.2.2. Method of Detecting AEs and SAEs

Care will be taken not to introduce bias when detecting an AE and/or a SAE. Open-ended and non-leading verbal questioning of the participant is the preferred method to inquire about AE occurrence.

9.2.3. Follow-up of AEs and SAEs

After the initial AE/SAE report, the Investigator is required to proactively follow each participant at subsequent visits/contacts. All SAEs, will be followed until the event is resolved, stabilized, otherwise explained, or the participant is lost to follow-up (as defined in Section 8.5). Further information on follow-up procedures is given in Appendix 5.

9.2.4. Regulatory Reporting Requirements for SAEs

- Prompt notification by the Investigator to the Sponsor of a SAE is essential so that legal obligations and ethical responsibilities towards the safety of

participants and the safety of a study treatment under clinical investigation are met.

- The Sponsor has a legal responsibility to notify both the local regulatory authority and other regulatory agencies about the safety of a study treatment under clinical investigation. The Sponsor will comply with country-specific regulatory requirements relating to safety reporting to the regulatory authority, Institutional Review Boards (IRB)/ IEC, and investigators.
- Investigator safety reports must be prepared for suspected unexpected serious adverse reactions (SUSAR) according to local regulatory requirements and Sponsor policy and forwarded to investigators as necessary.
- An Investigator who receives an Investigator safety report describing a SAE or other specific safety information (e.g., summary or listing of SAE) from the Sponsor will review and then file it along with the IB and will notify the IRB/IEC, if appropriate according to local requirements.

9.2.5. Cardiovascular and Death Events

For any cardiovascular events detailed in Section 13.5 and all deaths, whether or not they are considered SAEs, specific Cardiovascular (CV) and Death sections of the CRF are required to be completed. These sections include questions regarding cardiovascular (including sudden cardiac death) and non-cardiovascular death.

The CV CRFs are presented as queries in response to reporting of certain CV MedDRA terms. The CV information should be recorded in the specific cardiovascular section of the CRF within one week of receipt of a CV Event data query prompting its completion.

The Death CRF is provided immediately after the occurrence or outcome of death is reported. Initial and follow-up reports regarding death must be completed within one week of when the death is reported.

9.2.6. Pregnancy

- Details of all pregnancies in female partners of male participants will be collected after the start of study treatment and up to 7 days after the final dose of study medication.
- If a pregnancy is reported, the Investigator must inform GSK within 24 hours of learning of the pregnancy and should follow the procedures outlined in Appendix 6.

9.3. Treatment of Overdose

For this study, any total dose of GSK3036656 greater than the dose selected for that cohort (taken within a 23-hour period) will be considered an overdose.

The sponsor does not recommend specific treatment for an overdose.

In the event of an overdose, the Investigator/treating physician should:

1. Contact the Medical Monitor immediately.
2. Closely monitor the participant for AE/SAE and laboratory abnormalities until study treatment can no longer be detected systemically (at least 14 days).
3. Obtain a plasma sample for PK analysis if requested by the Medical Monitor (determined on a case-by-case basis).
4. Document the quantity of the excess dose as well as the duration of the overdosing in the CRF.

Decisions regarding dose interruptions or modifications will be made by the Investigator in consultation with the Medical Monitor based on the clinical evaluation of the participant.

9.4. Safety Assessments

Planned time points for all safety assessments are provided in the SoA.

9.4.1. Physical Examinations

- A complete physical examination will include, at a minimum, assessments of the Skin, Cardiovascular, Respiratory, Gastrointestinal and Neurological systems. Height and weight will also be measured and recorded.
- Investigators must pay special attention to clinical signs related to previous serious illnesses.

9.4.2. Vital Signs

- Temperature, pulse rate, respiratory rate, and blood pressure will be assessed.
- Blood pressure and pulse measurements will be assessed with a completely automated device. Position of the participant should be consistent across readings (e.g. semi-supine or supine). Manual techniques will be used only if an automated device is not available.
- Blood pressure and pulse measurements must be preceded by at least 5 minutes of rest for the participant in a quiet setting without distractions (e.g., television, cell phones).
- Vital signs (to be taken before blood collection for laboratory tests) will consist of at least 1 pulse and blood pressure measurement. Where a measurement is abnormal or significantly different from previous measurements, a further 2 readings will be taken. When vital signs are measured in triplicate, values should be taken at least 1 minute but not more than 5 minutes apart and recorded in the CRF.

9.4.3. Electrocardiograms

- Single 12-lead ECG will be obtained as outlined in the SoA using an ECG machine that automatically calculates the heart rate and measures PR, QRS,

uncorrected QT intervals. QTcF will be calculated using heart rate and QT by Friderica's formula (see SRM). Also refer to ECG stopping criteria listed in Section 8.1.2.

- In the event where triplicate ECG readings are required, 3 individual ECG tracings should be obtained as closely as possible in succession, but no more than 2 minutes apart. The full set of triplicates should be completed in less than 10 minutes. Stopping criteria are in Section 8.1.2

9.4.4. Echocardiograms

- Echocardiography will be performed at screening on all participants who have undergone all other screening assessments, have been found eligible for the study, and are willing to enrol into the study. It will also be done at follow-up on the participants in the cohort that is anticipated to receive the highest dose of GSK3036656 as well as on any participant who has received a lower dose of GSK3036656 if, in the opinion of the Investigator, their clinical status warrants it. The dose escalation committee may decide to perform this assessment in additional cohorts in this study.
- If feasible, the echocardiograms will be interpreted at a central core echocardiography lab and the screening and follow up echocardiograms for a particular participant will be obtained by the same technician.

9.4.5. Clinical Safety Laboratory Assessments

- Refer to Appendix 3 for the list of clinical laboratory tests to be performed and to the SoA for the timing and frequency.
- The Investigator must review the laboratory report, document this review, and record any clinically significant changes occurring during the study in the AE section of the CRF. The laboratory reports must be filed with the source documents. Clinically significant abnormal laboratory findings are those which are not associated with the underlying disease, unless judged by the Investigator to be more severe than expected for the participant's condition.
- All laboratory tests with values considered clinically significantly abnormal during participation in the study or within 24 hours after the last dose of study treatment should be repeated until the values return to normal or baseline or are no longer considered significantly abnormal by the Investigator or medical monitor.
- If such values do not return to normal/baseline within a period of time judged reasonable by the Investigator, the aetiology should be identified and the Sponsor notified.
- All protocol-required laboratory assessments, as defined in Appendix 3, must be conducted in accordance with the SRM and the SoA.

9.5. Pharmacokinetics

- Blood samples of approximately 2 mL will be collected for measurement of plasma concentrations of GSK3036656 as specified in the SoA. Instructions for the collection and handling of biological samples will be provided by the Sponsor. The actual date and time (24-hour clock time) of each sample will be recorded.
- Samples will be used to evaluate the PK of GSK3036656. Samples collected for analyses of GSK3036656 plasma concentration may also be used to evaluate safety or efficacy aspects related to concerns arising during or after the study.
- Once the plasma has been analyzed for GSK3036656 any remaining plasma may be analyzed for other compound-related material and the results reported under a separate protocol.

9.6. Potential Surrogate Disease/Biomarkers

CCI

[redacted]

CCI

[redacted]

CCI [redacted]

9.7. Incidental findings

Incidental findings (IFs) in clinical research are findings with potential health or reproductive importance that are discovered in the course of conducting the research, but are beyond the aims of the study. IFs in this study may arise from the collecting and analysing of research images i.e. CXR or CCI [redacted] or in the tests or information collected to determine eligibility criteria i.e. abnormal vital signs, safety bloods, or ECG.

The principal investigator (PI) is responsible for handling an IF responsibly and promptly and members of the research team will promptly report a suspected IF. If an IF is discovered the PI will inform the participant and exercise his/her judgment on a case by case basis to determine the way forward that is in the best interest of the participant. The PI will make sure that all the necessary documentation for the referral of the participant is put together free of cost to the participant.

10. STATISTICAL CONSIDERATIONS

10.1. Sample Size Determination

10.1.1. Original Sample Size Calculation

The planned sample size for this study is up to 100 participants randomised depending on the number of cohorts (15 participants in each GSK3036656 treatment group); sufficient participants will be screened to achieve this sample size.

No formal hypothesis testing is to be tested in this study, hence no formal sample size calculation has been performed, however, simulations were conducted to determine the impact of the size of the study on the ability to detect a treatment effect based on the mean daily $\log_{10}$ decline in CFU/mL in participants treated with GSK3036656. Of note, whilst approximately 25 subjects in the study will be randomised to SoC, formal comparisons of GSK3036656 to SoC will not be made. The simulations performed therefore simply assess the change from baseline within each GSK3036656 treatment group.

For the purposes of this analysis, untreated subjects were assumed to have a mean daily change from baseline in $\log_{10}$CFU of zero, any decrease from baseline in $\log_{10}$CFU was therefore considered to suggest a treatment effect. Previous EBA studies indicate that the standard deviation for the change from baseline in $\log_{10}$CFU is approximately 0.2 in treated subjects, therefore this estimate was used as the standard deviation for the simulations. Potential changes from baseline of −0.08 to 0.02, in increments of 0.01 were investigated.

Data were simulated from a normal distribution, for each potential treatment effect.

Simulations were repeated 1000 times for each potential treatment effect and the number of times the mean change from baseline met the following criteria was calculated:

> $\geq$ 0.0 $\log_{10}$CFU (no response or negative response)
>
> $<$ 0.0 $\log_{10}$CFU (positive response)
>
> $\leq$ -0.06 $\log_{10}$CFU (good positive response, effect comparable with new individual drugs in recent EBA studies).

Simulations were repeated for sample sizes of 15, 20 and 25 per arm, the results for the percentage of studies showing a mean change from baseline below zero is shown in the following figure.

Percentage of Studies Showing a Mean Change < 0.0
Vs True Underlying Change from Baseline

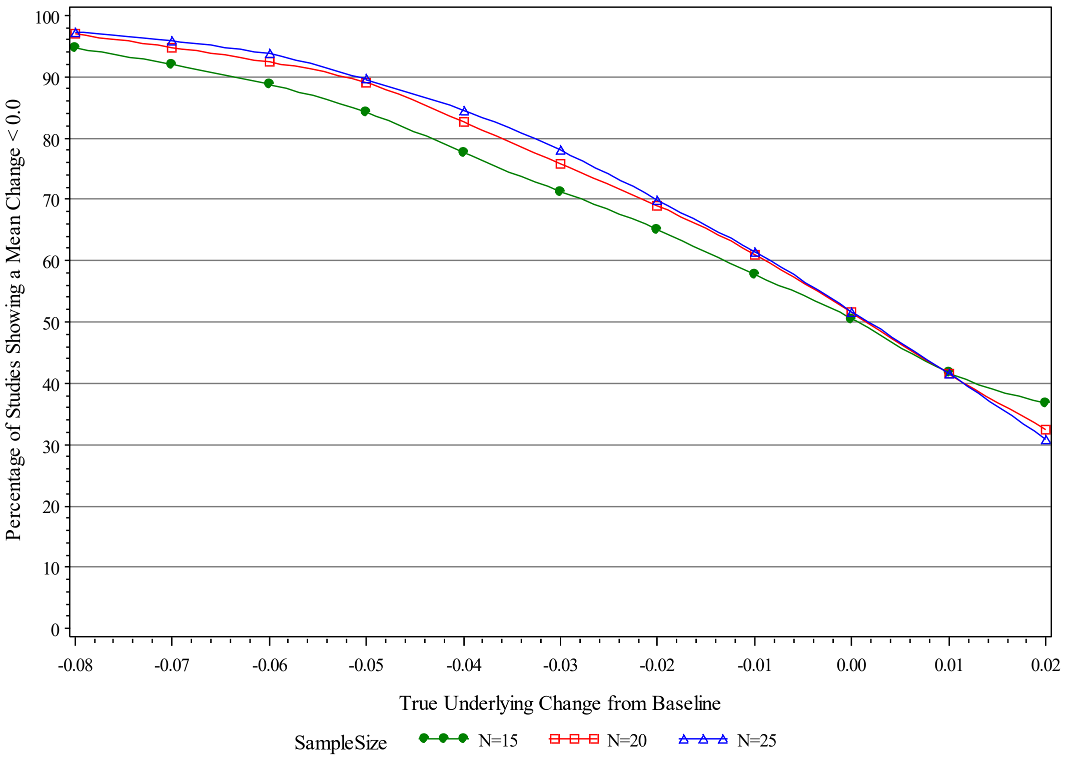

Assuming a true underlying change from baseline of −0.06, and a sample size of 15 participants per treatment arm, there is approximately 90% chance of the observed mean change being a negative value (i.e., a positive response will be observed). Increasing the sample size does not greatly improve the probability of observing a positive result based on this true underlying treatment difference of -0.06.

10.1.2. Sample Size Re-estimation

Due to the slower than anticipated rate of study recruitment, during recruitment to cohort 3 of the study, a review of the standard deviation of daily change from baseline in log10(CFU) was performed to assess if the initial estimate of the standard deviation (0.2) was comparable to the observed study data available at that time. The review included CFU data from participants in the first 2 cohorts and included only participants who completed assessments from baseline through to Day 14. The average daily change from baseline was calculated as change between baseline and Day 14 divided by 14, then the standard deviation summarised by treatment group. The results are displayed in the following table.

**Treatment Group**	**N**	**Daily Change from Baseline in Log10(CFU) Standard Deviation**
Standard-of-care	9	0.049
GSK3036656 5mg	15	0.067
GSK3036656 15mg	13	0.053

The observed standard deviations were smaller than used in the original sample size simulation therefore the sample size simulations described in Section 10.1 were repeated using standard deviations of 0.1 and 0.15 and including additional sample sizes of 9 and 12 participants per treatment arm. The results for the percentage of studies showing a mean change from baseline below zero, assuming a standard deviation of 0.15, is shown in the following figure.

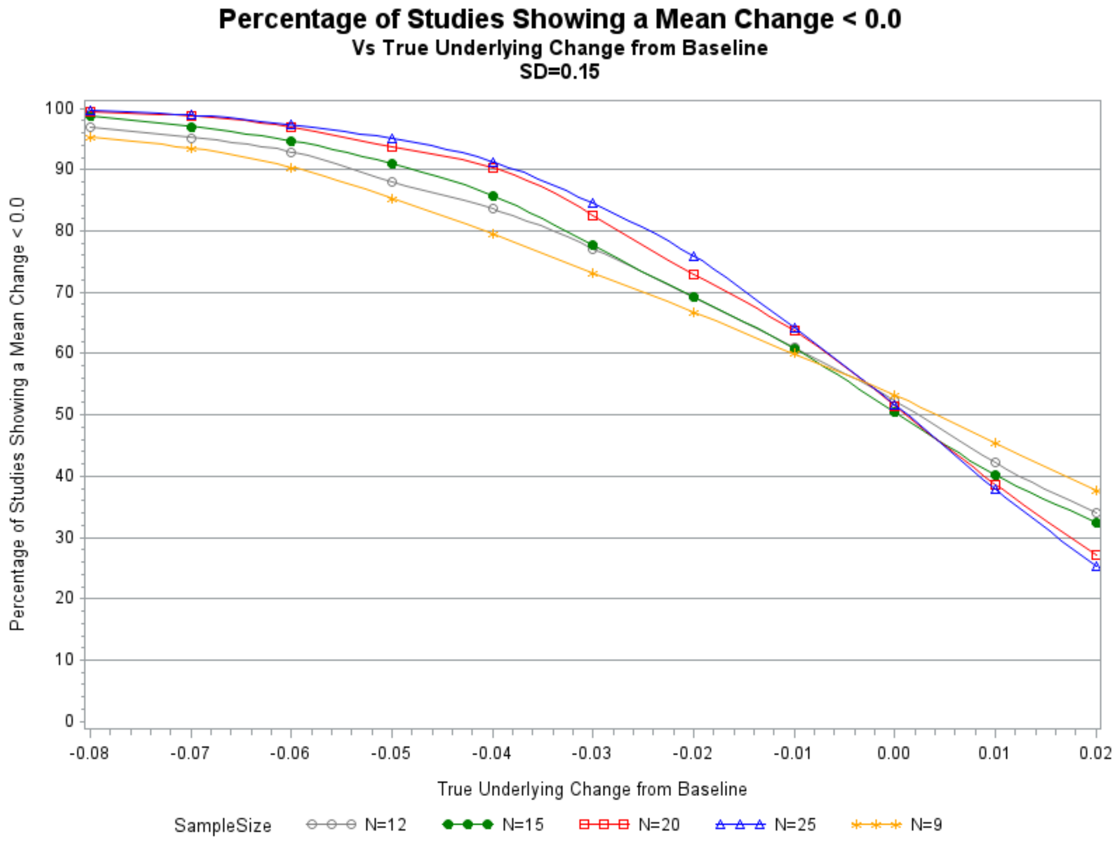

Assuming a standard deviation of 0.15, a sample size of 9 participants per treatment arm would provide approximately the same level of confidence as originally planned assuming a standard deviation of 0.2 and a sample size of 15 participants per treatment arm i.e. Assuming a true underlying change from baseline of -0.06 and a sample size of 9 participants per treatment arm, there is approximately 90% chance of the observed mean change being a negative value (i.e. a positive response). Therefore, this supports reducing the sample size in ongoing and future cohorts to 9 participants per GSK3036656 treatment arm.

10.2. Populations for Analyses

For purposes of analysis, the following populations are defined:

Population	Description
Screened	All participants who were screened for eligibility.
Enrolled	All participants who passed screening and entered the study.
Randomised	All participants who were randomly assigned to treatment in the study.
Pharmacokinetic (PK)	Participants in the safety population who received at least one dose of GSK3036656 and have at least one evaluable PK sample. Participants will be analysed according to the treatment actually received.
Efficacy	Participants in the safety population who provided at least two evaluable overnight sputum sample.
Safety	All randomized participants who received at least 1 dose of study treatment. Participants will be analysed according to the treatment they actually received.

Data from the standard of care arm will be pooled for the final analysis.

10.3. Statistical Analyses

10.3.1. Activity/Pharmacokinetic Analyses

Endpoint	Statistical Analysis Methods
Primary	Rate of change in log_{10}CFU per mL direct respiratory sputum samples over the period baseline to Day 14 (EBA CFU_{0-14}). This will be analysed using a mixed effects model including treatment, day, BMI and treatment-by-day as covariates.<br>This endpoint will be analysed using the efficacy population.
Secondary	**PK Endpoints**<br>Pharmacokinetic analysis will be the responsibility of the Clinical Pharmacology Modeling and Simulation Department, (CPMS), GlaxoSmithKline. Plasma GSK3036656 concentration-time data will be analyzed by non-compartmental methods with WinNonlin 5.2 or higher.

Endpoint	Statistical Analysis Methods
	Calculations will be based on the actual sampling times recorded during the study.<br><br>From the plasma concentration-time data, the following pharmacokinetic parameters will be determined, as data permit:<br><br>• maximum observed blood concentration (Cmax)<br>• time to Cmax (tmax)<br>• area under the plasma concentration-time curve [AUC_{0-t} and AUC_{0-24}]<br>• trough concentration (C_T).<br><br>AUC_{0-t} or AUC_{0-24} and Cmax may be used for assessment of dose proportionality. Trough concentration (C_T) samples collected on the specified days will be used to assess attainment of steady state.<br><br>Pharmacokinetic data will be presented in graphical and/or tabular form and will be summarized descriptively.<br><br>Statistical analyses of the pharmacokinetic parameter data will be the responsibility of Statistics, Programming and Data Strategy (SPDS), GlaxoSmithKline. Distributional assumptions underlying the statistical analyses will be assessed by visual inspection of residual plots. Normality will be examined by normal probability plots, while homogeneity of variance will be assessed by plotting the residuals against the predicted values for the model. Alternative analyses of the data will be performed if any of the model assumptions appear to be violated. Full details on the statistical aspects will be detailed in the Reporting and Analysis Plan (RAP).<br><br>**Dose Proportionality**<br><br>Dose proportionality will be assessed via analyses of AUC_{0-t} or AUC_{0-24} and Cmax. A statistical analysis will be performed using the power model. The analysis will be performed on log_e-transformed data. For each of these parameters a mixed effects model will be fitted with loge (dose) as a fixed effect and individual subject fitted as random effects. Estimates of the mean slopes of loge (dose) will be reported along with corresponding 90% confidence intervals (slope≈1 implies dose proportionality).<br><br>**Achievement of Steady State**<br><br>Trough concentration levels, C_T, collected pre-morning dose will be plotted by collection day and dose. To evaluate whether steady state was achieved, statistical analysis of C_T will be performed after a log-transformation of the dose data. A mixed effect model will be fitted with dose, day and dose-by-day interaction as fixed effects (with dose as a factor and day as a continuous covariate) and subject as a random effect. The coefficients of the

Endpoint	Statistical Analysis Methods
	slopes for the day effect for each dose, along with corresponding 90% confidence intervals, will be used to determine whether steady state was achieved.<br><br>**Efficacy/Activity Endpoints**<br><br>EBA CFU_{0-2}, EBA CFU_{2-14}, EBA TTP_{0-14}, EBA TTP_{0-2} and EBA TTP_{2-14} will be analysed similarly to the primary endpoint. These endpoints will be analysed using the efficacy population.
Exploratory	Will be analysed and reported separately.

10.3.2. Safety Analyses

All safety analyses will be performed on the Safety Population.

Endpoint	Statistical Analysis Methods
Secondary	Safety data will be presented in tabular and/or graphical format and summarised descriptively according to GSK's Integrated Data Standards Library (IDSL) standards.<br><br>**Adverse Events**<br><br>Adverse event reporting will be performed using the MedDRA (Medical Dictionaries for Regulatory Activities) coding system. Counting of adverse events will be based on the number of participants, not the number of AEs.<br><br>In addition to all adverse events, there will be separate summaries for:<br>i. Serious adverse events<br>ii. Drug-related adverse events<br>iii. Adverse events leading to treatment / study withdrawal<br><br>In addition, AEs will be displayed by CTCAE grade and severity.<br><br>**Clinical Laboratory Values / Vital Signs**<br><br>Change from baseline in clinical laboratory data (haematology, clinical chemistry and urinalysis) and vital signs data (SBP, DBP, heart rate and body temperature) will be summarised descriptively by dose group and timepoint.<br><br>Laboratory and vital signs data will also be evaluated by summarising the worst-case results as number and percentage of participants in each dose group.<br><br>**ECG**

Endpoint	Statistical Analysis Methods
	Change from baseline in ECG will be summarised descriptively by dose group and timepoint for all participants. A summary of ECG findings and QTc values will also be produced.

10.3.3. Other Analyses

CCI [redacted]

10.3.4. Efficacy Analyses

No formal interim analyses are planned for this study. However, safety, tolerability and pharmacokinetic data will be reviewed before each dose escalation. In addition, the primary efficacy endpoint may also be analysed as specified in Section 11.

11. DOSE ESCALATION

After the initial dose of GSK3036656, the decision to proceed to each subsequent dose level will be made by the Dose Escalation Committee (DEC) based on safety, tolerability and preliminary GSK3036656 pharmacokinetic data from the prior cohort. The DEC may also include in their deliberations, efficacy data, if that is available. Minimum requirements for data are as follows: PK data in at least 7 subjects receiving GSK3036656 and selected safety data from all subjects in a cohort. A dose escalation committee charter will be produced that will contain the following details:

- Committee members
- Data review requirements
- Process for informing the ethics committee of any safety concerns if appropriate
- Facility for requesting additional pertinent information form the investigator

Dose escalation will be based on population PK modelling (if feasible). Dose escalations will not be higher than 3-fold.

The population PK model will be a fit for purpose model. Candidate model selection will be primarily based on a significant reduction in the objective function value ($\geq$ 3.32, $\chi 2$ <0.05) and improvement in the fits of diagnostic scatter plots. Inter-individual variability will be modelled using an exponential error model and residual error will be described by a proportional, proportional plus additive, or additive error model, depending on the data. Model performance will be evaluated using a visual predictive check. Simulations for dose recommendations will be conducted to determine the probability of an individual subject exceeding the defined exposure margins for AUC and Cmax.

Alternatively, if a population PK model is not feasible, dose escalation will be based on a dose-exposure model of the form:

log(AUC) = a + b*log(DOSE) + c*log(BWT/70)

log(CMAX) = d + e*log(DOSE) + f*log(BWT/70)

where a and d are AUC and Cmax values, respectively; c = -0.75 and f = -1.00 are set values for allometric scaling; and b and e are scaling factors for dose proportionality.

A proposed dose will be acceptable if the predicted probability of an individual exceeding pre-defined exposure limits (Section 5.6) on any day of the dosing period, as estimated by PK modeling, is no more than 10%.

The primary endpoint may be analysed once data is available for 12 evaluable subjects, as per the sample size re-estimation calculations, on the second consecutive cohort that reaches or exceeds the target PK plasma exposure for efficacy (based on the murine infection model), and subsequent cohorts. The purpose of this efficacy analysis is to optimise dosing for subsequent cohorts (e.g. to help estimate the dose response curve) as well as to aid internal decision making.

The DEC is empowered to take the following decisions for subsequent cohorts:

- To select a higher dose (including a loading dose that may be given over 1 or more days) within the limits defined in Section 5.6.
- To select a dose lower than, or the same as, a preceding dose.
- To stop dosing.
- To include additional pharmacokinetic assessments.
- To include additional timepoints for an existing safety assessment.
- To adjust the haemoglobin exclusion and stopping criteria if no haematological abnormalities are observed in the review of safety data from the highest dose cohort (Cohort 3). For the subsequent cohort(s) receiving a lower dose of GSK3036656, the haemoglobin exclusion criterion will be lowered to <8.0 g/dL, and the haemoglobin stopping criterion will be lowered to <6.0 g/dL. Patients with a confirmed haemoglobin <8.0g/dL after randomisation will require enhanced observations, with a repeat haemoglobin level and discussion of patient status with the GSK medical monitor.

12. REFERENCES

Bloomfield DM, Kost JT, Ghosh K, Hreniuk D, Hickey LA, Guitierrez MJ, et al. The effect of moxifloxacin on QTc and implications for the design of thorough QT studies. Clin Pharmacol Ther 2008 Oct;84(4):475-80.

Burman E. The hunt for the elusive surrogate marker of sterilizing activity in tuberculosis treatment. Am J Resp Crit Care Med 2003;167:1299-1301.

CDC. 1993 Revised Classification System for HIV Infection and Expanded Surveillance Case Definition for AIDS Among Adolescents and Adults MMWR 1993 [https://www.cdc.gov/mmwr/preview/mmwrhtml/00018871.htm]

Diacon A, Maritz JS, Donald P. Early bactericidal activity of antituberculosis agents. In: Donald PR, Van Helden PD, eds. Antituberculosis Chemotherapy. Basel: Karger 2011a:213-19.

Diacon AH, Dawson R, Hanekom M, Narunsky K, Maritz SJ, Venter A, et al. Early bactericidal activity and pharmacokinetics of PA-824 in smear-positive tuberculosis patients. Antimicrob Agents Chemother. 2010;54:3402-7.

Diacon AH, Dawson R, Hanekom M, Narunsky K, Venter A, Hittel N, et al. Early bactericidal activity of delamanid (OPC-67683) in smear-positive pulmonary tuberculosis patients. Int J Tuberc Lung Dis. 2011b; 15:949-54.

Diacon AH, Dawson R, von Groote-Bidlingmaier F, Symons G, Venter A, Donald PR, et al. 14-day bactericidal activity of PA-824, bedaquiline, pyrazinamide, and moxifloxacin combinations: a randomised trial. The Lancet. 2012 Sep 21;380(9846):986-93.

Diacon AH, Dawson R, Von Groote-Bidlingmaier F, Symons G, Venter A, Donald PR, et al. Randomized dose-ranging study of the 14-day early bactericidal activity of bedaquiline (TMC207) in patients with sputum microscopy smear-positive pulmonary tuberculosis. Antimicrob Agents Chemother. 2013 May 1;57(5):2199-203.

Diacon AH, Patientia RF, Venter A, Van Helden PD, Smith PJ, McIlleron H, et al. Early bactericidal activity of high-dose rifampin in patients with pulmonary tuberculosis evidenced by positive sputum smears. Antimicrob Agents Chemother. 2007; 51(8): 2994-2996

Diacon AH, Pym A, Grobusch M, Patientia R, Rustomjee R, Page-Shipp L, et al. The diarylquinoline TMC207 for multidrug-resistant tuberculosis. N Engl J Med. 2009; 360:2397-405.

Diacon AH, van der Merwe L, Barnard M, von Groote-Bidlingmaier F, Lange C, García-Basteiro AL, et al. β-lactams against tuberculosis—new trick for an old dog? N Eng J Med. 2016 Jul 28;375(4):393-4.

Donald PR, Sirgel FA, Venter A, Parkin DP, Seifart HI, van de Wal BW, et al. Early bactericidal activity of antituberculosis agents. Expert Rev Anti Infect Ther. 2003; 1:141-55.

Donald PR, Sirgel FA, Venter A, Parkin DP, Van De Wal BW, Barendse A, et al. Early bactericidal activity of amoxicillin in combination with clavulanic acid in patients with sputum smear-positive pulmonary tuberculosis. Scandinavian journal of infectious diseases. 2001;33(6):466-9.

Furin JJ, Du Bois J, van Brakel E, Chheng P, Venter A, Peloquin CA, et al. Early Bactericidal Activity of AZD5847 in Patients with Pulmonary Tuberculosis. Antimicrob Agents Chemother. 2016;60(11):6591-9.

Jindani A, Aber VR, Edwards EA, Mitchison. The early bactericidal activity of drugs in patients with pulmonary tuberculosis. Am Rev Resp Dis 1980; 121(6): 939-949.

Matteelli A, Carvalho AC, Dooley KE, Kritski A. TMC207: the first compound of a new class of potent anti-tuberculosis drugs. Future Microbiol 2010 Jun; 5(6):849-58.

Mdluli, K and Nuermberger, E. Pre-Clinical Drug Combination Studies to Identify Novel Regimens for TB. In: Recent Advances in TB Drug Development symposium, 40th Union World Conference, International Union Against TB and Lung Disease, Cancun, 2009.

Rustomjee R, Diacon AH, Allen J, Venter A, Reddy C, Patientia RF, et al. Early bactericidal activity and pharmacokinetics of the diarylquinoline TMC207 in treatment of pulmonary tuberculosis. Antimicrob Agents Chemother. 2008; 52:2831-5

Wallis RS, Dawson R, Friedrich SO, Venter A, Paige D, Zhu T, et al. Mycobactericidal activity of sutezolid (PNU-100480) in sputum (EBA) and blood (WBA) of patients with pulmonary tuberculosis. PLoS One. 2014;9(4):e94462.

World Health Organization [WHO]. Global tuberculosis report 2016a. http://www.who.int/tb

World Health Organization [WHO]. The Short MDR-TB regimen 2016b. http://www.who.int/tb/Short_MDR_regimen_factsheet.pdf

13. APPENDICES

13.1. Appendix 1: Abbreviations and Trademarks

AE	Adverse Event
AIDS	Acquired Immune Deficiency Syndrome
ALT	Alanine Aminotransferase
AMX	Amoxicillin
AST	Aspartate Aminotransferase
AUC	Area Under the Plasma Concentration Time Curve
AUC_{0-24}	Area Under the Plasma Concentration Time Curve from Zero to End of Dosing Interval
BD	Twice Daily
CFU	Colony Forming Units
C_{max}	Maximum Observed Plasma Concentration
C_{min}	Minimum Observed Plasma Concentration at the End of the Dosing Interval
CRF	Case Report Form
CSR	Clinical Study Report
CTCAE	Common Terminology Criteria for Adverse Events
DBP	Diastolic Blood Pressure
DS	Drug Sensitive
EBA	Early Bactericidal Activity
EC	Ethics Committee
ECG	Electrocardiogram
EMB	Ethambutol
EWV	Early Withdrawal Visit
GCP	Good Clinical Practice
GGT	Gamma-Glutamyltransferase
HIV	Human Immunodeficiency Virus
HRZE	Isoniazid/Rifampicin/Pyrazinamide/Ethambutol
IB	Investigator's Brochure
ICF	Informed Consent Form
IV	Intravenous
IP	Investigational Product
IUATLD	International Union Against Tuberculosis and Lung Disease
INH	Isoniazid
LDH	Lactate Dehydrogenase
MDR	Multi Drug Resistant
MGIT	Mycobacterial Growth Indicator Tube
MIC	Minimum Inhibitory Concentration
MOXI	Moxifloxacin
Mtb	*Mycobacterium tuberculosis*
PCR	Polymerase Chain Reaction

PD	Pharmacodynamic
PK	Pharmacokinetic
PR	Electrocardiographic PR Interval
PZA	Pyrazinamide
QT	Electrocardiographic QT Interval
QTc	Corrected QT Interval
RAP	Reporting and Analysis Plan
RIF/RF	Rifampicin
SAE	Serious Adverse Event
SBP	Systolic Blood Pressure
SoC	Standard of care
SRM	Study Reference Manual
t	Time
t½	Apparent Terminal Elimination Phase Half-Life
TAR	Target Attainment Rate
TB	Tuberculosis
TEAEs	Treatment-Emergent Adverse Events
T_{max}	Time at Which C_{max} is Observed
TMIC	Time over Minimum Inhibitory Concentrations
TTP	Time to Sputum Culture Positivity
ULN	Upper Limit of Normal
WHO	World Health Organization
XDR	Extremely Drug Resistant

Trademark Information

Trademarks of the GlaxoSmithKline group of companies
NONE

Trademarks not owned by the GlaxoSmithKline group of companies
Rifafour e-275
WinNonlin

13.2. Appendix 2: EBA definitions

EBA	An agent's ability to kill mycobacteria originating within pulmonary cavities during the first weeks of treatment.
EBA $_{CFU}$	Determination of EBA by quantification of solid medium of viable Colony Forming Units (CFU) of *M. tuberculosis* from an overnight sputum collection.
EBA $_{TTP}$	Determination of EBA by measurement in an automated liquid culture system of time to positivity (TTP) of *M. tuberculosis* from an overnight sputum collection.

13.3. Appendix 3: Clinical Laboratory Tests

- The tests detailed in Table 2 will be performed by the local laboratory.
- Protocol-specific requirements for inclusion or exclusion of participants are detailed in Section 6 of the protocol.
- Additional tests may be performed at any time during the study as determined necessary by the Investigator or required by local regulations.

Table 2 Protocol-Required Safety Laboratory Assessments

Laboratory Assessments	Parameters			
Haematology	Platelet Count<br>RBC Count<br>Haemoglobin<br>Hematocrit<br>Coagulation test (INR, PT, APTT)	RBC Indices:<br>MCV<br>MCH<br>%Reticulocytes	WBC count with Differential:<br>Neutrophils<br>Lymphocytes<br>Monocytes<br>Eosinophils<br>Basophils	
Clinical Chemistry[1]	BUN	Potassium	Aspartate Aminotransferase (AST)	Total, indirect, and direct bilirubin
	Creatinine	Sodium	Alanine Aminotransferase (ALT) gamma-glutamyl transferase (GGT), lactic dehydrogenase (LDH), sodium, potassium, calcium (corrected for albumin), chloride	Total Protein
	Glucose (random/fasting)	Calcium (corrected for albumin)	Alkaline phosphatase	
Routine Urinalysis	• Specific gravity<br>• pH, glucose, protein, blood, micro-albumin ketones, bilirubin, urobilinogen, nitrite, sodium, leukocytes by dipstick<br>• Microscopic examination (if blood or protein is abnormal)			

Laboratory Assessments	Parameters
Other Screening Tests	• Urine drug screen (to include at minimum: amphetamines, cocaine, opiates.<br>• Serology (HIV antibody, hepatitis B surface antigen [HBsAg], and hepatitis C virus antibody)<br>• Cardiac safety screening: Troponin.

NOTES:

1. Details of liver chemistry stopping criteria and required actions and follow-up assessments after liver stopping or monitoring event are given in Section 8.1 and Appendix 7 All events of ALT ≥3 × upper limit of normal (ULN) and bilirubin ≥2 × ULN (>35% direct bilirubin) or ALT ≥3 × ULN and international normalized ratio (INR) >1.5, if INR measured, which may indicate severe liver injury (possible Hy's Law), must be reported as an SAE (excluding studies of hepatic impairment or cirrhosis).

13.4. Appendix 4: Study Governance Considerations

Regulatory and Ethical Considerations

- This study will be conducted in accordance with the protocol and with:
  - Consensus ethical principles derived from international guidelines including the Declaration of Helsinki and Council for International Organizations of Medical Sciences (CIOMS) International Ethical Guidelines
  - Applicable ICH Good Clinical Practice (GCP) Guidelines
  - Applicable laws and regulations
- The protocol, protocol amendments, ICF, Investigator Brochure, and other relevant documents (eg, advertisements) must be submitted to an IRB/IEC by the Investigator and reviewed and approved by the IRB/IEC before the study is initiated.
- Any amendments to the protocol will require IEC/IRB approval before implementation of changes made to the study design, except for changes necessary to eliminate an immediate hazard to study participants.
- The Investigator will be responsible for the following:
  - Providing written summaries of the status of the study to the IRB/IEC annually or more frequently in accordance with the requirements, policies, and procedures established by the IRB/EC
  - Notifying the IRB/IEC of SAE or other significant safety findings as required by IRB/IEC procedures
  - Providing oversight of the conduct of the study at the site and adherence to requirements of 21 CFR, ICH guidelines, the IRB/IEC, European regulation 536/2014 for clinical studies (if applicable), and all other applicable local regulations

Financial Disclosure

Investigators and sub-investigators will provide the sponsor with sufficient, accurate financial information as requested to allow the sponsor to submit complete and accurate financial certification or disclosure statements to the appropriate regulatory authorities. Investigators are responsible for providing information on financial interests during the course of the study and for 1 year after completion of the study.

Informed Consent Process

- The Investigator or his/her representative will explain the nature of the study to the participant or his/her legally authorized representative and answer all questions regarding the study.
- Participants must be informed that their participation is voluntary. Participants or their legally authorized representative will be required to sign a statement of

informed consent that meets the requirements of 21 CFR 50, local regulations, ICH guidelines, Health Insurance Portability and Accountability Act (HIPAA) requirements, where applicable, and the IRB/IEC or study center.

- The medical record must include a statement that written informed consent was obtained before the participant was enrolled in the study and the date the written consent was obtained. The authorized person obtaining the informed consent must also sign the ICF.
- Participants must be re-consented to the most current version of the ICF(s) during their participation in the study.
- A copy of the ICF(s) must be provided to the participant or the participant's legally authorized representative.

The ICF may contain a separate section that addresses the use of remaining mandatory samples for optional exploratory research in accordance with SOP-GSKF-410. The investigator or authorized designee will explain to each participant the objectives of the exploratory research. Participants will be told that they are free to refuse to participate and may withdraw their consent at any time and for any reason during the storage period. A separate signature will be required to document a participant's agreement to allow any remaining specimens to be used for exploratory research. Participants who decline to participate will not provide this separate signature.

Data Protection

- Participants will be assigned a unique identifier by the Sponsor. Any participant records or datasets that are transferred to the Sponsor will contain the identifier only; participant names or any information which would make the participant identifiable will not be transferred.
- The participant must be informed that his/her personal study-related data will be used by the Sponsor in accordance with local data protection law. The level of disclosure must also be explained to the participant.
- The participant must be informed that his/her medical records may be examined by Clinical Quality Assurance auditors or other authorized personnel appointed by the Sponsor and anTBiotic consortium members, by appropriate IRB/IEC members, and by inspectors from regulatory authorities.

Publication Policy

- The results of this study may be published or presented at scientific meetings. If this is foreseen, the Investigator agrees to submit all manuscripts or abstracts to the Sponsor before submission. This allows the sponsor to protect proprietary information and to provide comments.
- The Sponsor will comply with the requirements for publication of study results. In accordance with standard editorial and ethical practice, the Sponsor will generally support publication of multicenter studies only in their entirety and not as individual site data. In this case, a coordinating Investigator will be designated by mutual agreement.

- Authorship will be determined by mutual agreement and in line with International Committee of Medical Journal Editors authorship requirements.

Dissemination of Clinical Study Data

- Where required by applicable regulatory requirements, an Investigator signatory will be identified for the approval of the clinical study report. The Investigator will be provided reasonable access to statistical tables, figures, and relevant reports and will have the opportunity to review the complete study results at a GSK site or other mutually-agreeable location.
- GSK will also provide the Investigator with the full summary of the study results. The Investigator is encouraged to share the summary results with the study participants, as appropriate.
- The procedures and timing for public disclosure of the results summary and for development of a manuscript for publication will be in accordance with GSK Policy.
- A manuscript will be progressed for publication in the scientific literature if the results provide important scientific or medical knowledge.
- Anonymised analysable datasets from this study may be provided, through a secure system, following an independent assessment of the scientific merit of a rigorously defined research question from a third party.

Data Quality Assurance

- All participant data relating to the study will be recorded on printed or electronic CRF unless transmitted to the Sponsor or designee electronically (eg, laboratory data). The Investigator is responsible for verifying that data entries are accurate and correct by physically or electronically signing the CRF.
- The Investigator must maintain accurate documentation (source data) that supports the information entered in the CRF.
- The Investigator must permit study-related monitoring, audits, IRB/IEC review, and regulatory agency inspections and provide direct access to source data documents.
- The Sponsor or designee is responsible for the data management of this study including quality checking of the data.
- Study monitors will perform ongoing source data verification to confirm that data entered into the CRF by authorized site personnel are accurate, complete, and verifiable from source documents; that the safety and rights of participants are being protected; and that the study is being conducted in accordance with the currently approved protocol and any other study agreements, ICH GCP, and all applicable regulatory requirements.
- Records and documents, including signed ICF, pertaining to the conduct of this study must be retained by the Investigator for 25 years from the issue of the final Clinical Study Report (CSR)/ equivalent summary unless local regulations or institutional policies require a longer retention period. No records may be

destroyed during the retention period without the written approval of the Sponsor. No records may be transferred to another location or party without written notification to the Sponsor.

Source Documents

- Source documents provide evidence for the existence of the participant and substantiate the integrity of the data collected. Source documents are filed at the Investigator's site.
- Data reported on the CRF or entered in the eCRF that are transcribed from source documents must be consistent with the source documents or the discrepancies must be explained. The Investigator may need to request previous medical records or transfer records, depending on the study. Also, current medical records must be available.
- Definition of what constitutes source data can be found in the Source Document Agreement.

Study and Site Closure

GSK or its designee reserves the right to close the study site or terminate the study at any time for any reason at the sole discretion of GSK. Study sites will be closed upon study completion. A study site is considered closed when all required documents and study supplies have been collected and a study-site closure visit has been performed.

The Investigator may initiate study-site closure at any time, provided there is reasonable cause and sufficient notice is given in advance of the intended termination.

Reasons for the early closure of a study site by the Sponsor or Investigator may include but are not limited to:

- Failure of the Investigator to comply with the protocol, the requirements of the IRB/IEC or local health authorities, the Sponsor's procedures, or GCP guidelines
- Inadequate recruitment of participants by the Investigator
- Discontinuation of further study treatment development

13.5. Appendix 5: Adverse Events: Definitions and Procedures for Recording, Evaluating, Follow-up, and Reporting

Definition of AE

AE Definition
• An AE is any untoward medical occurrence in a clinical study participant, temporally associated with the use of a study treatment, whether or not considered related to the study treatment.<br>• NOTE: An AE can therefore be any unfavorable and unintended sign (including an abnormal laboratory finding), symptom, or disease (new or exacerbated) temporally associated with the use of a study treatment.

Events Meeting the AE Definition
• Any abnormal laboratory test results (hematology, clinical chemistry, or urinalysis) or other safety assessments (e.g., ECG, radiological scans, vital signs measurements), including those that worsen from baseline, considered clinically significant in the medical and scientific judgment of the Investigator (ie, not related to progression of underlying disease).<br>• Exacerbation of a chronic or intermittent pre-existing condition including either an increase in frequency and/or intensity of the condition.<br>• New conditions detected or diagnosed after study treatment administration even though it may have been present before the start of the study.<br>• Signs, symptoms, or the clinical sequelae of a suspected drug-drug interaction.<br>• Signs, symptoms, or the clinical sequelae of a suspected overdose of either study treatment or a concomitant medication. Overdose per se will not be reported as an AE/SAE unless it is an intentional overdose taken with possible suicidal/self-harming intent. Such overdoses should be reported regardless of sequelae.<br>• "Lack of efficacy" or "failure of expected pharmacological action" per se will not be reported as an AE or SAE. Such instances will be captured in the efficacy/activity assessments. However, the signs, symptoms, and/or clinical sequelae resulting from lack of efficacy/activity will be reported as AE or SAE if they fulfil the definition of an AE or SAE.<br>• The signs, symptoms, and/or clinical sequelae resulting from lack of efficacy/activity will be reported as AE or SAE if they fulfil the definition of an AE or SAE. Also, "lack of efficacy" or "failure of expected pharmacological action" constitutes an AE or SAE.

**Events NOT Meeting the AE Definition**
• Any clinically significant abnormal laboratory findings or other abnormal safety assessments which are associated with the underlying disease, unless judged by the Investigator to be more severe than expected for the participant's condition.<br>• The disease/disorder being studied or expected progression, signs, or symptoms of the disease/disorder being studied, unless more severe than expected for the participant's condition.<br>• Medical or surgical procedure (e.g., endoscopy, appendectomy): the condition that leads to the procedure is the AE.<br>• Situations in which an untoward medical occurrence did not occur (social and/or convenience admission to a hospital).<br>• Anticipated day-to-day fluctuations of pre-existing disease(s) or condition(s) present or detected at the start of the study that do not worsen.

Definition of SAE

If an event is not an AE per definition above, then it cannot be an SAE even if serious conditions are met (eg, hospitalization for signs/symptoms of the disease under study, death due to progression of disease).

**A SAE is defined as any untoward medical occurrence that, at any dose:**
**a. Results in death**
**b. Is life-threatening**<br>The term 'life-threatening' in the definition of 'serious' refers to an event in which the participant was at risk of death at the time of the event. It does not refer to an event, which hypothetically might have caused death, if it were more severe.
**c. Requires inpatient hospitalization or prolongation of existing hospitalization**<br>In general, hospitalization signifies that the participant has been detained (usually involving at least an overnight stay) at the hospital or emergency ward for observation and/or treatment that would not have been appropriate in the physician's office or outpatient setting. Complications that occur during hospitalization are AE. If a complication prolongs hospitalization or fulfils any other serious criteria, the event is serious. When in doubt as to whether "hospitalization" occurred or was necessary, the AE should be considered serious.<br><br>Hospitalization for elective treatment of a pre-existing condition that did not worsen from baseline is not considered an AE.

**d.**	**Results in persistent disability/incapacity**
•	The term disability means a substantial disruption of a person's ability to conduct normal life functions.
•	This definition is not intended to include experiences of relatively minor medical significance such as uncomplicated headache, nausea, vomiting, diarrhoea, influenza, and accidental trauma (eg, sprained ankle) which may interfere with or prevent everyday life functions but do not constitute a substantial disruption.
**e.**	**Is a congenital anomaly/birth defect**
**f.**	**Other situations:**
•	Medical or scientific judgment should be exercised in deciding whether SAE reporting is appropriate in other situations such as important medical events that may not be immediately life-threatening or result in death or hospitalization but may jeopardize the participant or may require medical or surgical intervention to prevent one of the other outcomes listed in the above definition. These events should usually be considered serious.
	Examples of such events include invasive or malignant cancers, intensive treatment in an emergency room or at home for allergic bronchospasm, blood dyscrasias or convulsions that do not result in hospitalization, or development of drug dependency or drug abuse.

Definition of Cardiovascular Events

**Cardiovascular Events (CV) Definition:**
Investigators will be required to fill out the specific CV event page of the CRF for the following AEs and SAEs:<br>• Myocardial infarction/unstable angina<br>• Congestive heart failure<br>• Arrhythmias<br>• Valvulopathy<br>• Pulmonary hypertension<br>• Cerebrovascular events/stroke and transient ischemic attack<br>• Peripheral arterial thromboembolism<br>• Deep venous thrombosis/pulmonary embolism<br>• Revascularization

Recording AE and SAE

AE and SAE Recording
• When an AE/SAE occurs, it is the responsibility of the Investigator to review all documentation (eg, hospital progress notes, laboratory, and diagnostics reports) related to the event.<br>• The Investigator will then record all relevant AE/SAE information in the CRF.<br>• It is **not** acceptable for the investigator to send photocopies of the participant's medical records to GSK in lieu of completion of the GSK /AE/SAE CRF page.<br>• There may be instances when copies of medical records for certain cases are requested by GSK. In this case, all participant identifiers, with the exception of the participant number, will be redacted on the copies of the medical records before submission to GSK.<br>• The Investigator will attempt to establish a diagnosis of the event based on signs, symptoms, and/or other clinical information. Whenever possible, the diagnosis (not the individual signs/symptoms) will be documented as the AE/SAE.
**Assessment of Intensity**
The investigator will make an assessment of intensity for each AE and SAE reported during the study and assign it to categories according to the definitions in CTCAE v5.0.<br><br>An event is defined as 'serious' when it meets at least 1 of the predefined outcomes as described in the definition of an SAE.

Assessment of Causality
• The Investigator is obligated to assess the relationship between study treatment and each occurrence of each AE/SAE.<br>• A "reasonable possibility" of a relationship conveys that there are facts, evidence, and/or arguments to suggest a causal relationship, rather than a relationship cannot be ruled out.<br>• The Investigator will use clinical judgment to determine the relationship.<br>• Alternative causes, such as underlying disease(s), concomitant therapy, and other risk factors, as well as the temporal relationship of the event to study treatment administration will be considered and investigated.<br>• The Investigator will also consult the Investigator's Brochure (IB) and/or Product Information, for marketed products, in his/her assessment.<br>• For each AE/SAE, the Investigator **must** document in the medical notes that he/she has reviewed the AE/SAE and has provided an assessment of causality.<br>• There may be situations in which an SAE has occurred and the investigator has minimal information to include in the initial report to GSK. However, **it is very**

**important that the investigator always make an assessment of causality for every event before the initial transmission of the SAE data to GSK.**<br>• The Investigator may change his/her opinion of causality in light of follow-up information and send an SAE follow-up report with the updated causality assessment.<br>• The causality assessment is one of the criteria used when determining regulatory reporting requirements.

Follow-up of AE and SAE
• The Investigator is obligated to perform or arrange for the conduct of supplemental measurements and/or evaluations as medically indicated or as requested by GSK to elucidate the nature and/or causality of the AE or SAE as fully as possible. This may include additional laboratory tests or investigations, histopathological examinations, or consultation with other health care professionals.<br>• If a participant dies during participation in the study or during a recognized follow-up period, the investigator will provide GSK with a copy of any post-mortem findings including histopathology.<br>• New or updated information will be recorded in the originally completed CRF.<br>• The Investigator will submit any updated SAE data to GSK within 24 hours of receipt of the information.

Reporting of SAE to GSK

SAE Reporting to GSK via Electronic Data Collection Tool
• The primary mechanism for reporting SAE to GSK will be the electronic data collection tool.<br>• If the electronic system is unavailable, then the site will use the paper SAE data collection tool (see next section) in order to report the event within 24 hours.<br>• The site will enter the SAE data into the electronic system as soon as it becomes available.<br>• The Investigator or medically-qualified sub-investigator must show evidence within the eCRF (e.g., check review box, signature, etc.) of review and verification of the relationship of each SAE to IP/study participation (causality) within 72 hours of SAE entry into the eCRF.<br>• After the study is completed at a given site, the electronic data collection tool will be taken off-line to prevent the entry of new data or changes to existing data.<br>• If a site receives a report of a new SAE from a study participant or receives updated data on a previously reported SAE after the electronic data collection tool has been taken off-line, then the site can report this information on a paper SAE form (see next section) or to the medical monitor by telephone.

• Contacts for SAE reporting can be found in the SRM.

SAE Reporting to GSK via Paper CRF
• Email facsimile transmission of the SAE paper CRF is the preferred method to transmit this information to the medical monitor.<br>• In rare circumstances and in the absence of facsimile/email capability, notification by telephone is acceptable with a copy of the SAE data collection tool sent by overnight mail or courier service.<br>• Initial notification via telephone does not replace the need for the Investigator to complete and sign the SAE CRF pages within the designated reporting time frames.<br>• Contacts for SAE reporting can be found in the SRM.

13.6. Appendix 6: Contraceptive Guidance and Collection of Pregnancy Information

Contraception Guidance

Male participants

- Male participants with female partners of child-bearing potential are eligible to participate if they agree to ONE of the following:
  - Are abstinent from penile-vaginal intercourse as their usual and preferred lifestyle (abstinent on a long term and persistent basis) and agree to remain abstinent for a period of 90 days after the last dose of study medication (one spermatogenesis cycle).
  - Agree to use a male condom plus an additional method of contraception with a failure rate of <1% per year as described in Table 3 when having penile-vaginal intercourse with a woman of childbearing potential, for a period of 90 days after the last dose of study medication.
- Men with a pregnant or breastfeeding partner must agree to remain abstinent from penile-vaginal intercourse or use a male condom during each episode of penile penetration during the treatment period and for 6 days after (five terminal half-lives).
- In addition, male participants must refrain from donating sperm during the treatment period and for 90 days after (one spermatogenesis cycle).

Table 3 Highly Effective Contraceptive Methods

**Highly Effective Contraceptive Methods That Are User Dependent** [a]<br>*Failure rate of <1% per year when used consistently and correctly.*
Combined (estrogen- and progestogen-containing) hormonal contraception associated with inhibition of ovulation<br>• oral<br>• intravaginal<br>• transdermal
Progestogen-only hormonal contraception associated with inhibition of ovulation<br>• injectable
**Highly Effective Methods That Are User Independent**
• Implantable progestogen-only hormonal contraception associated with inhibition of ovulation<br>• Intrauterine device (IUD)<br>• Intrauterine hormone-releasing system (IUS)<br>• bilateral tubal occlusion
Vasectomized partner<br>*(A vasectomized partner is a highly effective contraception method provided that the partner is the sole male sexual partner of the WOCBP and the absence of sperm has been confirmed. If not, an additional highly effective method of contraception should be used.)*
Sexual abstinence<br>(*Sexual abstinence is considered a highly effective method only if defined as refraining from heterosexual intercourse during the entire period of risk associated with the study drug. The reliability of sexual abstinence needs to be evaluated in relation to the duration of the study and the preferred and usual lifestyle of the participant.)*

NOTES:
a.Typical use failure rates may differ from those when used consistently and correctly. Use should be consistent with local regulations regarding the use of contraceptive methods for participants in clinical studies.

Collection of Pregnancy Information

Male participants with partners who become pregnant

- Investigator will attempt to collect pregnancy information on any male participant's female partner of a male study participant who becomes pregnant while participating in this study. This applies only to participants who receive study treatment.
- After obtaining the necessary signed informed consent from the pregnant female partner directly, the Investigator will record pregnancy information on the

appropriate form and submit it to GSK within 24 hours of learning of the partner's pregnancy.

- Partner will also be followed to determine the outcome of the pregnancy. Information on the status of the mother and child will be forwarded to GSK
- Generally, follow-up will be no longer than 6 to 8 weeks following the estimated delivery date. Any termination of the pregnancy will be reported regardless of fetal status (presence or absence of anomalies) or indication for procedure.

13.7. Appendix 7: Liver Safety: Required Actions and Follow-up Assessments

Liver chemistry stopping criteria and required follow up assessments

Elevated liver enzymes, transient liver dysfunction, hepatotoxicity and hepatitis are listed as side-effects in the package inserts for Rifafour e-275 (Rifafour, 2002) or equivalent generic alternative, associated with multiple drug components. Therefore, participants allocated to Rifafour e-275 (or equivalent generic alternative) treatment who experience Liver Chemistry Stopping criteria are not mandated in the first instance to undergo those follow-up assessments (which are intended to investigate aetiology). The investigations may still be performed, if required in the judgement of the investigator.

Liver Chemistry Stopping Criteria	
**ALT-absolute**	ALT ≥ 5xULN
**ALT Increase**	ALT ≥ 3xULN persists for ≥4 weeks
**Bilirubin[1, 2]**	ALT ≥ 3xULN **and** bilirubin ≥ 2xULN (>35% direct bilirubin)
**INR[2]**	ALT ≥ 3xULN **and** INR>1.5, if INR measured
**Cannot Monitor**	ALT ≥ 3xULN and cannot be monitored weekly for 4 weeks
**Symptomatic[3]**	ALT ≥ 3xULN associated with symptoms (new or worsening) believed to be related to liver injury or hypersensitivity

Required Actions and Follow up Assessments	
**Actions**	**Follow Up Assessments**
• **Immediately** discontinue study treatment<br>• Report the event to GSK **within 24 hours**<br>• Complete the liver event CRF and complete an SAE data collection tool if the event also meets the criteria for an SAE[2]<br>• Perform liver chemistry event follow up assessments<br>• Monitor the participant until liver chemistries resolve, stabilize, or return to within baseline (see **MONITORING** below)<br>• **Do not restart/rechallenge** participant with study treatment unless allowed per protocol	<u>*For all study participants:*</u><br>• Obtain INR and recheck with each liver chemistry assessment until the transaminases values show downward trend<br>• Record the appearance or worsening of clinical symptoms of liver injury, or hypersensitivity, on the AE report form<br>• Record use of concomitant medications on the concomitant medications report form including acetaminophen, herbal remedies, other over the counter medications.

and GSK Medical Governance approval **is** granted (see below)<br>• If restart/rechallenge **not allowed per protocol or not granted**, permanently discontinue study treatment and continue participant in the study for any protocol specified follow up assessments<br><br>**MONITORING:**<br>**<u>For bilirubin or INR criteria:</u>**<br>• Repeat liver chemistries (include ALT, AST, alkaline phosphatase, bilirubin) and perform liver event follow up assessments within **24 hrs**<br>• Monitor participants twice weekly until liver chemistries resolve, stabilize or return to within baseline<br>• A specialist or hepatology consultation is recommended<br>**<u>For All other criteria:</u>**<br>• Repeat liver chemistries (include ALT, AST, alkaline phosphatase, bilirubin) and perform liver event follow up assessments within **24-72 hrs**<br>• Monitor participants weekly until liver chemistries resolve, stabilize or return to within baseline	• Record alcohol use on the liver event alcohol intake case report form (CRF) page<br><br>*<u>For participants on GSK303656 arm</u>*<br>• Viral hepatitis serology[4]<br>• Obtain blood sample for pharmacokinetic (PK) analysis as soon as possible, and certainly within 14 days after last dose [5]<br>• Serum creatine phosphokinase (CPK) and lactate dehydrogenase (LDH).<br>• Fractionate bilirubin, if total bilirubin≥2xULN<br>• Obtain complete blood count with differential to assess eosinophilia<br><br>**<u>For bilirubin or INR criteria:</u>**<br>*<u>For participants on GSK303656 arm only</u>*<br>• Auto-immune screen: Anti-nuclear antibody, anti-smooth muscle antibody, Type 1 anti-liver kidney microsomal antibodies, and quantitative total immunoglobulin G (IgG) or gamma globulins.<br>• Serum paracetamol/acetaminophen adduct high performance liquid chromatography (HPLC) assay (quantifies potential acetaminophen contribution to liver injury in participants with definite or likely acetaminophen use in the preceding week [James, 2009]).<br>• Liver imaging (ultrasound, magnetic resonance, or computerised tomography) and /or liver biopsy to evaluate liver

	disease; complete Liver Imaging and/or Liver Biopsy CRF pages.

1. Serum bilirubin fractionation should be performed if testing is available. If serum bilirubin fractionation is not immediately available, discontinue study treatment for that subject if ALT ≥ 3xULN **and** bilirubin ≥ 2xULN.. Additionally, if serum bilirubin fractionation testing is unavailable, **record presence of detectable urinary bilirubin on dipstick**, indicating direct bilirubin elevations and suggesting liver injury.
2. All events of ALT ≥ 3xULN **and** bilirubin ≥ 2xULN (>35% direct bilirubin) or ALT ≥ 3xULN **and** INR>1.5, if INR measured which may indicate severe liver injury (possible 'Hy's Law'), **must be reported as an SAE (excluding studies of hepatic impairment or cirrhosis)**; INR measurement is not required and the threshold value stated will not apply to participants receiving anticoagulants
3. New or worsening symptoms believed to be related to liver injury (such as fatigue, nausea, vomiting, right upper quadrant pain or tenderness, or jaundice) or believed to be related to hypersensitivity (such as fever, rash or eosinophilia)
4. Includes: Hepatitis A IgM antibody; Hepatitis B surface antigen (HbsAg) and Hepatitis B Core Antibody (IgM); Hepatitis C RNA; Cytomegalovirus IgM antibody; Epstein-Barr viral capsid antigen IgM antibody (or if unavailable, obtain heterophile antibody or monospot testing); Hepatitis E IgM antibody

For PK sampling; record the date/time of the PK blood sample draw and the date/time of the last dose of study treatment prior to PK blood sample draw on the CRF. If the date or time of the last dose is unclear, provide the participant's best approximation. If the date/time of the last dose cannot be approximated OR a PK sample cannot be collected in the time period indicated above, do not obtain a PK sample. Instructions for sample handling and shipping are in the SRM.

Liver chemistry criteria for a Liver Monitoring Event and follow-up actions:

These Increased Monitoring Criteria apply to all participants who experience a Liver Event, regardless of which treatment the participant has been randomized to.

**Liver Chemistry Increased Monitoring Criteria – Liver Monitoring Event**	
**Criteria**	**Actions**
ALT ≥3×ULN and <5×ULN **and** bilirubin <2×ULN, **without** symptoms believed to be related to liver injury or hypersensitivity, **and** who can be monitored weekly for 4 weeks.	• Notify the GSK medical monitor **within 24 hours** of learning of the abnormality by email or phone to discuss participant safety.<br>• Participant can continue study intervention.<br>• Participant must return weekly for repeat liver chemistries (ALT, AST, ALP, bilirubin) until they resolve, stabilise or return to within baseline.<br>• If at any time participant meets the liver chemistry stopping criteria, proceed as described above<br>• If, after 4 weeks of monitoring, ALT <3×ULN and bilirubin <2×ULN, monitor participant

	twice monthly until liver chemistries normalize or return to within baseline

The site will complete the liver event eCRF (Monitoring Event) in InForm and should also consider whether the AE reporting guidelines apply.

References

James LP, Letzig L, Simpson PM, Capparelli E, Roberts DW, Hinson JA, Davern TJ, Lee WM. Pharmacokinetics of Acetaminophen-Adduct in Adults with Acetaminophen Overdose and Acute Liver Failure. Drug Metab Dispos 2009; 37:1779-1784.

Rifafour e-275 tablet electronic package inserts. 2002. Accessed 20 Feb 2019.: http://home.intekom.com/pharm/hmr/rifaf275.html

CCI
[redacted]

13.9. Appendix 9: Protocol Amendment History

The Protocol Amendment Summary of Changes Table for the current amendment is located directly before the Table of Contents (TOC).

Amendment 4 (17-FEB-2021)

Overall Rationale for the Amendment:

- To allow for Standard of Care medication administered in the study to be either Rifafour e-275 or an equivalent generic alternative.
- Addition of ‘e-275’ to all references to Rifafour, for consistency.
- To allow for analysis of the primary endpoint data to be performed on additional cohorts.

CHANGES

Synopsis / Overall Design:

A single-centre, open-labelled, clinical trial in up to five sequential cohorts of participants with rifampicin-susceptible tuberculosis (DS-TB). Each cohort will involve participants being randomised to one of two possible treatments: either GSK3036656 or Standard-of-care (SoC) regimen for DS-TB (**i.e.** Rifafour e-275 **or equivalent generic alternative**). The ratio of participants assigned to GSK3036656 vs Rifafour **e-275** (**or equivalent generic alternative**) will be 3:1.

Schedule of Activities / PK sampling time and events table:

C. PK sampling is only to be performed on participants receiving GSK3036656. PK samples will not be taken from participants receiving Rifafour **e-275 (or equivalent generic alternative)**.

5. Study Design / Overall Design

The study will comprise up to 5 cohorts of participants. It is intended that 15 participants in each cohort will receive GSK3036656 and 5 will receive standard-of-care regimen for DS-TB (**i.e.** Rifafour e-275 **or equivalent generic alternative**). There will be no placebo in this study. The study will be randomized, but not blinded (i.e., it will be ‘open-label’) with neither participant nor Investigator blinded to treatment. Study participants will revert to the standard treatment for TB (**i.e.** Rifafour **e-275 or equivalent generic alternative**) once the study treatment (Day 1 to Day 14) has been completed. All laboratory staff involved in analysing and reporting the primary and secondary change in log_{10}CFU counts and TTP endpoints results will be blinded to treatment allocation. Cohorts will proceed sequentially starting A through D, with preliminary safety, tolerability and pharmacokinetic data from the previous cohort reviewed prior to selecting the dose for the next cohort

5.2 Study Schematic

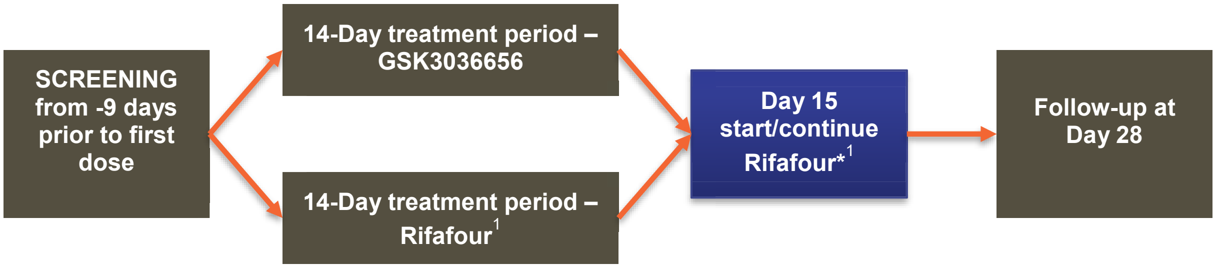

*This is not part of the study but is the post-study definitive treatment of TB.
[1]**Rifafour e-275 or equivalent generic alternative.**

5.4 Participant and Study Completion

Following completion of the study treatment phase and after discharge from hospital the participants will be provided with their first dose/s of standard TB therapy (**i.e.** Rifafour e-275 **or equivalent generic alternative**) with appropriate counselling. All participants will be referred to the local community TB clinics to receive the remainder of a full course of standard antituberculosis chemotherapy according to national guidelines. The participants will be provided with a referral letter to take with them to the TB clinic. Within a week of the participant's discharge from the study clinic, a follow-up call will be made by the study site staff to the TB clinic to determine if the participant attended the clinic.

5.5 Scientific Rationale for Study Design

The Rifafour **e-275 (or equivalent generic alternative)** treatment group is included as a benchmark for the EBA quantitative mycobacteriology and to evaluate whether HRZE in this population gives similar EBA results to that demonstrated in prior studies with this combination. A negative control arm, where subjects are left untreated, would be unethical due to the availability of effective treatment and the consequences of not treating TB.

7.1 Treatments Administered

**Study Treatment Name:**	GSK3036656	Rifafour e-275 **(or equivalent generic equivalent)**
**Dosage formulation:**	Capsule	Tablet
**Unit dose strength(s)/Dosage level(s):**	1 mg or 5 mg or 25 mg GSK3036656 capsules<br>(as free base equivalent)	Standard dose: 150/75/400/275 mg of Rifampicin, Isoniazid, Pyrazinamide and Ethambutol respectively per tablet
**Route of Administration**	Oral	Oral
**Dosing instructions:**	Refer to study reference manual	Refer to South African National TB Treatment Guidelines (including pyridoxine as required)
**Packaging and Labelling**	Study Treatment will be provided in HDPE bottles. Each HDPE bottle will be labelled as required per country requirement.	Will be provided by the manufacturer.
**Manufacturer**	WuXi AppTec (1 and 5 mg capsules)<br>Aptuit (25 mg capsule)	**Rifafour e-275:** Sanofi-Aventis or appropriate alternative<br>**Equivalent Generic alternative to Rifafour e-275:** Appropriate suppliers

Rifafour e-275 (or equivalent generic alternative).

Some participants will be treated with standard, intensive phase pulmonary TB treatment as recommended in the SA National TB Treatment Guidelines to verify that the laboratory assays used measure the expected magnitude of activity. This is HRZE (Rifafour e-275 or **equivalent generic alternative)** (HRZE: H=isoniazid: R=rifampicin: Z=pyrazinamide: E=ethambutol). The Rifafour **e-275 (or equivalent generic alternative)** treatment group is included as a benchmark for the EBA quantitative mycobacteriology and to evaluate whether HRZE in this population gives similar EBA results to that demonstrated in prior studies using this combination.

Rifafour e-275 **(or equivalent generic alternative)** Package Insert contains its known and potential risks and benefits.

8. Discontinuation Criteria

If this results in the patient being placed on standard of care (**i.e.** Rifafour **e-275 or equivalent generic alternative**), then all assessments relevant to Rifafour **e-275 (or equivalent generic alternative)** will be performed as directed by the protocol (for example, GSK3036656 pharmacokinetic assessments are not required, since the participant is not being dosed with GSK3036656).

11. Dose Escalation

The primary endpoint may be analysed for all available cohorts after the final visit of the last subject on the second consecutive cohort that reaches or exceeds the target PK plasma exposure for efficacy (based on the murine infection model)**, and subsequent cohorts**. The purpose of this efficacy analysis is to optimise dosing for subsequent cohorts (e.g. to help estimate the dose response curve) as well as to aid internal decision making.

13.7 Appendix 7: Liver Safety: Required Actions and Follow-up Assessments

Elevated liver enzymes, transient liver dysfunction, hepatotoxicity and hepatitis are listed as side-effects in the package inserts for Rifafour e-275 (see References) **or equivalent generic alternative**, associated with multiple drug components. Therefore, participants allocated to Rifafour e-275 **(or equivalent generic alternative)** treatment who experience Liver Chemistry Stopping criteria are not mandated in the first instance to undergo those follow-up assessments (which are intended to investigate aetiology). The investigations may still be performed, if required in the judgement of the investigator.

Amendment 3 24-JUL-2019

Overall Rationale for the Amendment:

To document the increase in exposure limits and change in eligibility criteria following the availability of new non-clinical data.

Further changes made to the protocol are detailed in the table below.

Section #	Description of Change	Brief Rationale
Schedule of Assessments table	CCI [redacted]	Duplication of tests unnecessary.
Schedule of Assessments table; footnote E	Removal of "oral" from footnote	Site primarily use tympanic or axillary temperature assessment.
Schedule of Assessments table; footnote F	Minor re-wording.	Clarity of understanding.
Schedule of Assessments table; footnote H	Remove footnote reference to Day 15	Not required, discrepancy in the protocol found by Investigator.
Schedule of Assessments table; footnote K	CCI [redacted]	Minimisation of risk to participants.
Schedule of Assessments table; footnote O	Added text that MIC samples sent to GSK, Spain will not be reported in CSR.	To clarify that the MIC testing of the bacterial isolates sent for further characterization to GSK Spain will be reported and analysed separately.
SoA	Addition of an ECG at the Early Withdrawal Visit (EWV)	Mistakenly omitted in previous versions
SoA PK table	Addition of 10-minute window for the pre-dose ('0') timepoint on Day 14.<br><br>Clarification that timing of PK samples from Day 14 is relative to dosing on Day 14<br><br>Addition of PK sample window allowances table	Window necessary to ensure all assessments and procedures can be completed in a timely manner.<br><br>Ensure clarity of understanding.

Section #	Description of Change	Brief Rationale
9.6.1	CCI [redacted]	[redacted]
Appendix 3	Removal of text "The results of each test must be entered into the CRF"<br><br>Removal of 'urine alcohol'<br><br>Removal of urine hCG, FSH and oestradiol in females	Data will be transferred electronically<br><br>Urine alcohol test erroneously listed in Appendix 3 but not in table of events. This test will not be done. This is standard GSK template text that should not have been part of this protocol.<br><br>Females will be excluded
3.3	New AUC & Cmax exposure limits and supporting non-clinical information	New non-clinical data supports increasing the AUC & Cmax limits dictating dose level choices in the study
5.1	Increase in maximum number of cohorts	To provide flexibility for 3 potential scenarios: a lower dose is included to help describing the exposure-response relationship, a dose needs to be repeated or a higher dose than currently projected is feasible due to observed PK being overpredicted.
5.6	Updated probability rates table based on increased AUC & Cmax exposure limits	New non-clinical data supports increasing the AUC & Cmax limits dictating dose level choices in the study
6.1	Removal of eligibility of females<br><br>Other criteria re-wording	New non-clinical data indicates females must not be enrolled.
6.2	Addition of 'Female' to exclusion criteria<br><br>Other criteria re-wording	New non-clinical data indicates females must not be enrolled.
6.3.1	Removal of UV exposure restriction	New information from non-clinical studies now supports removal of the restriction.

Section #	Description of Change	Brief Rationale
6.3.2	Removal of a requirement to abstain from water for a period of time after dosing	Study treatment can be administered without regard to food/water.
8	Addition of text clarifying discontinuation of study treatment	A subject can be discontinued from receiving study treatment but remain in the study.
9.1	Clarification of technical details for MIC provided in other documentation	Per standard practice, technical details are included in a different study document.
9.2.6	Updated text to reflect exclusion of females	New non-clinical data indicates females must not be enrolled.
9.4.1	Removal of the description of a brief physical exam	Not required in this protocol and was template text that should have been removed originally.
9.6.1	CCI [redacted]	Text required for internal GSK purposes
9.6.2	Replacing text to clarify genetic testing.<br>Addition of text clarifying process for analysis	Human DNA testing will not be conducted.<br>Text required for internal GSK purposes
10.1	Updated number of subjects	Consistency with updated design intent.
10.3.1	Updated clarification of reporting of exploratory endpoints	Clarity of understanding of intent.
10.3.4	Updated text concerning data review triggers	Clarity of understanding of intent.
11	Updated text concerning data review triggers	Clarity of understanding of intent.
Appendix 3	Updated screening tests	Consistency with design intent.
Appendix 4	Addition of text clarifying entities that may receive data	Consistency with design intent.
Appendix 6	Removal of text related to pregnancy testing.	Females will not be included, updates made reflecting this design intent.

Section #	Description of Change	Brief Rationale
Appendix 7	Incorporation of PK monitoring algorithm table previously contained within a separate study document.<br><br>Clarification of requirements for procedures relative to which treatment a participant is receiving.	Ensure clarity of understanding and remove duplication<br><br>Remove requirement for unnecessary procedures in participants receiving Rifafour.

CHANGES

Synopsis

Overall Design:

A single-centre, open-labelled, clinical trial in up to **five** ~~four~~ sequential cohorts of participants with rifampicin-susceptible tuberculosis (DS-TB). Each cohort will involve participants being randomised to one of two possible treatments: either GSK3036656 or Standard-of-care (SoC) regimen for DS-TB (Rifafour e-275). The ratio of participants assigned to GSK3036656 vs Rifafour will be 3:1.

Number of Participants: up to approximately **100** ~~80~~ participants will be enrolled.

Treatment Groups and Duration: Up to **5** 4 cohorts; 20 participants in each cohort (15 participants will receive GSK3036656, and 5 participants will receive SoC in each cohort).

Section 2 Schedule of Activities

**Period**	**Screening**			**Dosing**						**Follow-up**	
**Visit**	**1**	**2**	**3**	**4**	**5 to 10**	**11**	**12 to 16**	**17**	**18**	**Early Withdrawal**	**19**
**Day**	**(-9 to -3)** [A]	**-2**[B]	**-1**	**1**	**2 to 7**[C]	**8**	**9 to 13**[C]	**14**	**15**		**28 (± 7)**
Documentation of Positive GeneXpert and/or TB smear (TB clinic/site of initial diagnosis)	X										
Written Informed Consent	X										
Demography (including smoking history)	X										
Medical & Treatment History	X										
Inclusion/Exclusion/Eligibility Assessment		X									
Chest X-ray	X										
Physical Examination[D]	X			X	X	X	X	X	X	X	X
Vital Signs [E]	X	X	X	X	X	X	X	X	X	X	X
12-Lead ECG	X							X		**X**	X
Randomisation[M]		X									
Echocardiogram (may be conducted at any of the screening visits)		X								X[S]	X[S]
Blood PK[F]							X[F]	X	X	X	
Spot Sputum (confirm TB & adequate bacterial load) [G]	X										
Rifampicin Resistance Test (rapid)[G] **(microscopy and GeneXpert tests may be repeated on an overnight sputum sample if needed)**	X										
Haematology, Clinical Chemistry, Urinalysis [H]	X			X	X	X		X		X	X
Cardiac safety biomarkers[T]		X		X	X			X			
Urine Drug Screen[I]	X	X[I]									

Period	Screening			Dosing						Follow-up	
Visit	1	2	3	4	5 to 10	11	12 to 16	17	18	Early Withdrawal	19
Day	(-9 to -3) [A]	-2[B]	-1	1	2 to 7[C]	8	9 to 13[C]	14	15		28 (± 7)
~~Urine Pregnancy Test[R]~~		~~X~~						~~X~~	~~X~~		
HIV Test (and CD4 Count if HIV confirmed)	X										
CCI [redacted]				X	X			X		X	
[redacted]				X	X			X		X	
[redacted]		X						X			
Hospital Admission	X[B]	X[B]									
Overnight Sputum[L]	X	X	X	X	X	X	X	X			
IP Administration and Compliance Check[N]				X	X	X	X	X			
Mycobacteriology Assessments [O]		X	X					X			
Hospital Discharge									X	X	
Concomitant Medication		X	X	X	X	X	X	X	X	X	X
Adverse Events[P]				X	X	X	X	X	X	X	X
Point of Care Blood Glucose or HbA1c[Q]		X						X			

A. The Visit 1 (day -9 to -3) time period will be up to a maximum of 7 days but will be kept as short as possible.

B. Participants can proceed with the Visit 2 (day -2) assessments as soon as their Visit 1 (day -9 to -3) assessments have been completed i.e. Visits 1 and 2 may occur on the same day as long as the screening results are available in time for randomisation. Participants may be hospitalised during the entire pre-treatment period if the Investigator considers it advisable for reasons of safety or compliance.

C. All events listed as occurring on Visit 5 (day 2) to Visit 10 (day 7) and Visit 12 (day 9) to Visit 16 (day 13) will be conducted each day during these visits.

D. Height (m) will only be collected once at Visit 1 (day -9 to -3). A full physical examination will be performed at Visit 1 (day -9 to -3), with symptom-directed physical examinations at Visit 17 (day 14), Visit 19 (day 28) and Early Withdrawal Visit (EWV). Symptom directed physical examinations may be conducted as required during the study (visits 4 to 16). Requirements for the physical examination are specified in the SRM.

E. Systolic blood pressure (SBP) and diastolic blood pressure (DBP) (mmHg), heart rate (beats per minute [bpm]), body temperature (°C) and weight (kg). To be performed within 4 hours prior to the first daily dosing on Visit 4 (day 1) through to Visit 17 (day 14) and within 2 hours before the time dosing would have occurred on Visit 18 (day 15). On days where the following assessments are done the order should be: vital signs before blood draws for PK or safety assessments.

F. PKs will be performed as noted in the PK table. A single pre-dose PK sample will be collected on each of Days 12 & 13, and serial PK blood sampling will occur on Day 14 (see PK table below). At EWV a PK sample will be **before** ~~taken pre-dose of~~ the time of the planned ~~first~~ daily dosing. If this is not possible, a spontaneous PK sample will be taken.

G. Sputum smear microscopy and molecular rapid test for confirmation of Mycobacterium tuberculosis and rifampicin susceptibility (GeneXpert). **If** the first spot sputum **sample shows an unfavourable** result, the tests may be repeated on a freshly collected spot sputum or overnight sputum sample and that result used instead.

~~H. Laboratory assessments: haematology (haemoglobin, haematocrit, red blood cell count, white blood cell count with differential, platelet count). Clinical Chemistry (albumin, urea, creatinine, direct, indirect and total bilirubin, total protein, alkaline phosphatase, aspartate aminotransferase (AST), alanine aminotransferase (ALT), gamma glutamyl transferase (GGT), lactic dehydrogenase (LDH), sodium, potassium, calcium (corrected for albumin), chloride, random/fasting glucose). Urinalysis (pH, specific gravity, protein, glucose, micro albumin, ketones, bilirubin, creatinine, nitrite, sodium, urobilinogen, blood, leukocytes, microscopy). Coagulation test (INR, PT, APTT). To be performed on Visit 4 (day 1), Visit 6 or 7 (day 3 or 4), Visit 11 (day 8).~~. **Refer to Appendix 3 for details.**

I. Urine drug screen: cocaine, amphetamines and opiates. Urine drug screen can be repeated at Visit 2 (day -2) for participants who were not hospitalized at Visit 1 (day -9 to -3).

J. CCI [redacted]

K.

L. Overnight sputum sampling may start at Screening visit (day -9 to -3) or on Visit 2 (day –2) and will continue daily until Visit 7 (day 4). Additional sampling will be conducted at visit 9 (day 6), Visit 11 (day 8), Visit 13 (day 10), Visit 15 (day 12) and Visit 17 (day 14). Sputum sampling will stop on the morning of Visit 18 (day 15). Sputum collection will start in the afternoons at 15:00 hours (±1 hour) and continue for 16 hours overnight. The 16-hour sputum sampling for each of the sampling days must be finished prior to the administration of the next day's IP.

Overnight sputum collection may be collected as many times as required over the screening period until an eligible sample is obtained. Of the pre-treatment period samples collected, only the Visit 2 (day-2) and Visit 3(day -1) overnight sputum samples will be used for the efficacy endpoint tests.

The day sputum collection starts reflects the day to which that sample applies. e.g. a sample whose collection starts on day 1 and ends on day 2, is designated as the day 1 overnight sputum sample (and results).

M. Randomization may occur once all the screening results are available and the Investigator has determined that the participant is eligible for the trial.

N. After the first dose of IP, subsequent doses of IP will be administered 23 to 25 hours after the previous dose.

O. Minimum inhibitory concentration (MIC) and speciation of the infecting organism by polymerase chain reaction (PCR) will be estimated from a culture collected on a pre-treatment day (day-1 or day-2) and from the last available culture on a treatment day (day 14 or earlier, but not earlier than day 8). Drug susceptibility testing of *M. tuberculosis* for sensitivity to rifampicin with a molecular method will be tested at Visit 1. Susceptibility to INH, Rif, EMB and PZA will be tested on a culture grown from a baseline sample (day -2 or day -1). The analysis of any bacterial isolates sent to GSK, Spain for further characterisation in the event of MIC change will not be reported in the clinical study report and will be reported separately.

P. Adverse events (AE) will be collected by the Investigator from the time a participant receives his/her first dose of IP through to the Visit 19 (day 28) Follow up Visit.

Q. CCI [redacted]

~~R.~~ CCI [redacted]

S. Only to be performed on participants receiving the anticipated highest dose or on participants with changes in clinical status warranting a follow-up echo in the opinion of the investigator. The dose escalation committee may decide to perform this assessment in additional cohorts within this study.

T. Samples will be assayed for: Troponin I. The first two samples at least 24 hours apart, one during the screening phase and one prior to the first dose of study treatment. Day 14 sample window is ±24 hours. Third sample to be taken on Day 3.

**STUDY VISIT**	**Visit ~~12 to~~ 15**	**Visit 16**	**Visit 17**										**Visit 18**
**STUDY DAY**	**Day 12**	**Day 13**	**Day 14**										**Day 15**
**STUDY HOUR[B]**			**0**	**0.5**	**1**	**1.5**	**2**	**3**	**4**	**6**	**8**	**12**	**24**
Pharmacokinetic sampling[C]	X[A]	X[A]	X**[D]**	X	X	X	X	X	X	X	X	X	X

A. Sample to be taken within 30 minutes pre-dose.

B. All values are relative to the time of day that the study treatment is administered **on Day 14**.

C. PK sampling is only to be performed on participants receiving GSK3036656. PK samples will not be taken from participants receiving Rifafour.

D. Sample to be taken within 10 minutes pre-dose

PK Sample timing window allowances

Sample timepoint	Sample collection window allowed
Pre dose	Not applicable (see footnotes above)
0h - 4h post	± 5 min
> 4h – 12h post	± 15 min
> 12h – 24h post	± 1 h

Section 3.3.1 Risk Assessment

Potential Risk of Clinical Significance	Summary of Data/Rationale for Risk	Mitigation Strategy
**Investigational Product (IP) GSK3036656**		
Reduction in red blood cell count	**Non-Clinical**<br><br>In a 4-week rat study (doses 3, 10 & 30 mg/kg/d) - dose-dependent decline in reticulocytes (by up to 52% in males and 62% in females) at day 7 followed by normal levels by day 14 and increased levels by day 29/30 compared to controls. Hgb levels decreased by 10-13% compared to controls on day 14 and day 29/30.<br><br>In a 4 week dog study (doses 5, 10 & 20 mg/kg/d) - mild dose-dependent decrease in Hgb on day 14 (by about 10% compared to controls) with recovery by week 4. Reticulocyte counts unaffected. Dogs not bled on day 7.<br><br>No histopathological changes in bone marrow or other relevant histopathology in the 4-week studies.<br><br>**Clinical**<br><br>In the 201040 study, a possible trend of decreased haemoglobin from baseline to follow-up was noted in aggregate haematology data. The mean change from baseline to follow-up was -4.9 g/L in the 15 mg	Standard safety haematology and clinical chemistry assessments will be performed and both trends and changes outside normal range will be monitored as part of laboratory safety assessments.<br><br>Participants whose haemoglobin drops below pre-specified limits (<8.0 g/dL) will be withdrawn from the study (Section 8.1.3).<br><br>~~While women of non-childbearing potential are permitted in this study, women who are susceptible to heavy periods or heavy vaginal bleeding or spotting will be excluded in order to minimize blood loss and avoid confounding effects on the interpretation of haematology parameters.~~

Potential Risk of Clinical Significance	Summary of Data/Rationale for Risk	Mitigation Strategy
	repeat dose cohort, compared to -1.5 g/L in the placebo group. None of the values hit potential clinical importance (PCI) criteria, and declines were not clinically relevant.<br><br>No clinically significant trends in reticulocyte values were observed, and there were no values of potential clinical importance reported for reticulocytes.	
GSK3036656 is cleared through the kidneys	The main route of clearance for GSK3036656 is renal and there is therefore a risk that participants with low creatinine clearance will accumulate GSK3036656 and exceed predefined exposure limits.	Participants with a creatinine clearance less than 75 mL/min have been excluded.
Reduction in white blood cell count	**Non-Clinical**<br><br>In the 4-week rat study, decrease of up to 37% in total leucocyte count on day 29/30 compared to controls.<br><br>In the 4-week dog study, no reported effects on white blood cells.<br><br>**Clinical**<br><br>No clinically significant trends in leukocyte or neutrophil count values were observed. Two subjects showed PCI values. One subject had low neutrophil	White blood cell count will be monitored as part of laboratory safety assessments. Any clinically significant changes will be followed up until levels are not clinically significant.<br><br>Participants developing neutrophil counts <500/mm^3 (1.0x10^9/L) will be withdrawn from the study (Section 8.1.3).

Potential Risk of Clinical Significance	Summary of Data/Rationale for Risk	Mitigation Strategy
	count (normal range 1.57-6.81 × 10^9/L) from screening (1.38) until follow-up (1.41), and also showed low leukocyte count (normal range 3.3-9.8 × 10^9/L) on Day 4 (2.5), Day 10 (2.6) and Day 14 (2.9) and was within normal range at follow-up. Given the low screening values, this observation was considered likely related to the subject's Black or African American race. A second subject had a single low neutrophil count at follow-up (1.48).	
Heart valvular and vascular pathology	**Non-Clinical**<br><br>In a 7-day dog dose range finding (DRF) study – minimal focal subendocardial hemorrhage on the atrial surface of the left atrioventricular valve observed in both animals at the top dose of 60 mg/kg/d.<br><br>In a 10-day dog investigative study at 65 mg/kg/d – vascular lesions (including minimal focal necrosis and inflammatory infiltrate) in 3 out of 8 dogs.<br><br>In the 4-week dog GLP study - no heart or valve changes were observed up to the top dose of 20 mg/kg/day and there was no increase in inflammatory markers.	This pathology is not monitorable in humans.<br><br>~~To avoid risk to humans, dosing will be limited to doses at which individual exposures [AUC (0-24)] do not exceed 4.9 µg.h/ml – 1/30 of the dog NOAEL exposure (from the 4-week GLP study) at which no heart pathology was seen.~~<br><br>**To avoid risk to study participants, dosing will be limited to doses at which individual exposures do not exceed AUC0-24 and Cmax to 15µg.hr/ml and 1.5µg/ml respectively.**

Potential Risk of Clinical Significance	Summary of Data/Rationale for Risk	Mitigation Strategy
	**In the 17 week GLP dog study there were no heart or valve changes up to the top dose of 45mg/kg/day.**<br><br>**In the 17 week rat study there were no changes in cardiac pathology up to the top dose of 30mg/kg/day.**<br>Valvular or vascular pathology were not observed in rats. This pathological change is not commonly seen in animals, and its mechanism and significance to humans are not clear. A correlation between the presence of hemodynamic changes and the presence of this pathology is suspected, but currently unproven.<br><br>**Clinical**<br><br>Echocardiograms were performed at screening and follow up in the 201040 FTIH study in order to exclude participants with pre-existing valve or other cardiac abnormalities from the study, and to detect the presence of abnormalities after completion of the study. No abnormalities were detected.<br><br>**No SAEs, or AEs of cardiovascular nature were reported. There was no change from baseline in heart rate observed at any dose. No trends in blood pressure change were observed. Cardiac telemetry and echocardiogram-related**	**These exposures are 10-fold below the lowest NOAEL achieved in the 17 week studies (rat)**<br>In addition, although the pathology is not monitorable in humans, echocardiograms will be performed for all participants at screening and, for subjects receiving the highest dose of GSK3036656 or whose clinical status warrants it, also at follow-up (or early withdrawal). Cardiac troponin will be measured.

Potential Risk of Clinical Significance	Summary of Data/Rationale for Risk	Mitigation Strategy
	interpretations were within normal range. There were no clinically significant ECG observations. There were no troponin I abnormalities in participants after receiving study drug	

Section 5.1

The study will comprise up to 5 cohorts of participants. It is intended that 15 participants in each cohort will receive GSK3036656 and 5 will receive standard-of-care regimen for DS-TB (Rifafour e-275).

CCI [redacted]

…

Section 5.6

Exposure limits of $AUC_{0\text{–}24}$ of 15 µg.h/mL and Cmax of 1.5 ug/mL were set, based on information in Section 3.3.

A loading dose of GSK3036656 may be administered on the first day **(or over multiple days where required in order to not exceed AUC and Cmax exposure limits)** of study treatment administration in each cohort.

Table 1

Loading Dose (mg)[a]	Probability Day 1 [c]	Maintenance Dose (mg)[b]	probability Day 14[d]	Target Attainment Rate[e]
**15**	**<1%**	**5**	**<1%**	**<1%**
**25**	**<1%**	**10**	**<1%**	**100%**
**50**	**<1%**	**20**	**<1%**	**100%**
**75**	**6.90%**	**30**	**5.80%**	**100%**
**[a] Day 1**				
**[b] Day 2-14**				
**[c]per trial probability of an individual participant exceeding the exposure limits [$AUC_{0\text{–}24}$ = 15 ug.h/mL or Cmax = 1.5 ug/mL] on Day 1 (simulations based on a lower limit of 75 mL/min for creatinine clearance)**				
**[d] per trial probability of an individual participant exceeding the exposure limits [$AUC_{0\text{–}24}$ = 15 ug.h/mL or Cmax = 1.5 ug/mL] on Day 14 (simulations based on a lower limit of 75 mL/min for creatinine clearance)**				
**[e] per trial probability of >90% of participants exceeding the target exposure for efficacy**				

Section 6.1

Sex

10. Male ~~or female of non-childbearing potential~~

a. Male participants:

A male participant with female partners of child-bearing potential must agree to use contraception as detailed in Appendix 5.

~~**Female participants:**~~

~~A female participant is eligible to participate if she is not pregnant (see Appendix 5), not breastfeeding, and at least one of the following conditions applies:~~

~~Pre-menopausal females with one of the following:~~

~~Tubal ligation~~

~~Hysteroscopic tubal occlusion procedure with follow-up confirmation of bilateral tubal occlusion or documented bilateral salpingectomy~~

~~Hysterectomy~~

~~Bilateral Oophorectomy~~

~~Postmenopausal females: postmenopausal will be defined as 12 months of spontaneous amenorrhea without an alternative medical cause. Post-menopausal status will be confirmed by a simultaneous FSH and estradiol levels test.~~

11. Capable of giving signed informed consent as described in **Appendix 3** which includes compliance with the requirements and restrictions listed in the informed consent form (ICF) and in this protocol.

Section 6.2

~~6. History of photosensitivity~~

8. ~~Known or suspected, current or history of within the past 2 years,~~ **Current** alcohol or drug abuse, that is, in the opinion of the Investigator, sufficient to compromise the safety or cooperation of the participant.

12. Treatment received with any drug active against Mtb (including but not limited to isoniazid, ethambutol, fluoroquinolones, rifampicin), or with immunosuppressive medications such as TNF-alpha inhibitors or systemic or inhaled corticosteroids **(used for > 5 consecutive days)**, within 2 weeks prior to screening. Refer to the SRM for details.

14. Participants with the following abnormal laboratory values at screening as ~~defined by~~ **graded by** the enhanced Common Terminology Criteria for Adverse Events (**CTCAE v 5 2017**):

~~16 Women who are susceptible to heavy periods or heavy vaginal bleeding, in the investigator's judgement or spotting will be excluded in order to minimize blood loss and avoid confounding effects on the interpretation of haematology parameters.~~

Section 6.3.1

~~Participants should minimise any exposure to sources of ultra-violet radiation, e.g. refrain from sunbathing; use of tanning salons/sunbeds, starting on the day of first dosing until five half-lives (6 days) after the last dose on day 14 (as per Section 3.3).~~

Section 6.3.2

- ~~Water is allowed at dosing in order to administer study medication. No water is allowed from dosing until 2 hours afterwards~~; **Water is allowed ad libitum at all other times.**

Section 8

If study treatment/investigational product is interrupted or stopped this does not automatically result in the participant being withdrawn from the study. The investigator will make all effort to retain the patient in the study, provided the participant continues to provide their consent, and to complete and record all scheduled safety assessments required by the protocol. For the avoidance of all doubt: this does not prejudice the rights of the investigator and study participants under ICH GCP and under applicable laws. The study participant retains the right to withdraw consent at any time for any reason. The investigator also retains the right to withdraw the patients from the study if in the investigator's judgement, withdrawal is judged clinically necessary to protect a participant's safety.

If this results in the patient being placed on standard of care (RifaFour), then all assessments relevant to RifaFour will be performed as directed by the protocol (for example, GSK3036656 pharmacokinetic assessments are not required, since the participant is not being dosed with GSK3036656).

Section 7.2

On **or prior to day 1,** eligible participants will be assigned a unique number (randomization number) in ascending numerical order at the study site.

Section 8.1.1

Discontinuation of study treatment for abnormal liver tests is **also** required when:

Section 9.1

Mycobacterial Characterisation:

Cultures from baseline and from day 1**4** ~~5~~ sputum will be kept for determination of the minimum inhibitory concentration (MIC) of the investigational agents that the subject was treated with. If a day 1**4** ~~5~~ culture is not available, the last available culture after day

8 will be kept. **In the event of significant changes in the MIC, bacterial isolates will be sent to the GSK Spain laboratory for further characterisation. Technical details are provided in the SRM.**

Section 9.2.6

Details of all pregnancies in ~~pregnancies in female participants and, if indicated,~~ female partners of male participants will be collected after the start of study treatment and up to 7 days after the final dose of study medication.

- ~~Abnormal pregnancy outcomes (e.g., spontaneous abortion, fetal death, stillbirth, congenital anomalies, ectopic pregnancy) are considered SAE.~~

Section 9.4.1

- ~~A brief physical examination will include, at a minimum, assessments of the skin, lungs, cardiovascular system, and abdomen (liver and spleen).~~

Section 9.4.2

- Vital signs (to be taken before blood collection for laboratory tests) will consist of at least 1 pulse and blood pressure measurement. Where a measurement is abnormal or significantly different from previous measurements, this will be repeated. Where BP and pulse is repeated above, a further 2 readings recorded at least 1 min apart will be obtained for confirmation and ~~the average of the 2~~ recorded in the CRF.

Section 9.4.3

~~At each time point at which~~ **In the event where** triplicate ECG **readings are** required, 3 individual ECG tracings should be obtained as closely as possible in succession, but no more than 2 minutes apart.

Section 9.6.1

CCI [redacted]

CCI
[redacted]

Section 10.1

The planned sample size for this study is ~~approximately 60-80~~ **up to 100** participants randomised depending on the number of cohorts (15 participants in each GSK3036656 treatment group); sufficient participants will be screened to achieve this sample size.

Of note, whilst approximately ~~20~~ **25** subjects in the study will be randomised to SOC, formal comparisons of GSK3036656 to SOC will not be made.

Section 10.3.1

Exploratory	Will be ~~described in the RAP~~ **analysed and reported separately.**

Section 10.3.4

No formal interim analyses are planned for this study. However, safety, tolerability and pharmacokinetic data will be reviewed before each dose escalation. In addition, the primary efficacy endpoint may also be analysed after the final visit of the last subject for the cohort ~~which receives the anticipated highest dose~~ **as specified in section 11**. ~~for further details.~~

Section 11

The primary endpoint may be analysed for all available cohorts after the final visit of the last subject on ~~for~~ **the second consecutive cohort that reaches or exceeds the target PK plasma exposure for efficacy (based on the murine infection model).** ~~which receives the anticipated highest dose (as determined by the DEC).~~ The purpose of this efficacy analysis is to optimise dosing for subsequent cohorts (e.g. to help estimate the dose response curve) as well as to aid internal decision making.

The DEC is empowered to take the following decisions for subsequent cohorts:

To select a higher dose **(including a loading dose that may be given over 1 or more days)** within the limits defined in Section 5.6.

Section 13.3

Other Screening Tests	• ~~Folicle-stimulating hormone and estradiol (as to confirm post-menopausal status)~~<br>• ~~Alcohol and~~ Urine drug screen (to include at minimum: amphetamines, cocaine, opiates.<br>• ~~Urine human chorionic gonadotropin (hCG) pregnancy test (as needed for women of childbearing potential)[2]~~<br>• Serology (HIV antibody, hepatitis B surface antigen [HBsAg], and hepatitis C virus antibody)<br>• Cardiac safety screening: Troponin.<br>• ~~The results of each test must be entered into the CRF.~~

1. Details of liver chemistry stopping criteria and required actions and follow-up assessments after liver stopping or monitoring event are given in Section 8.1 and Appendix 7 All events of ALT ≥3 × upper limit of normal (ULN) and bilirubin ≥2 × ULN (>35% direct bilirubin) or ALT ≥3 × ULN and international normalized ratio (INR) >1.5, if INR measured, if INR measured, which may indicate severe liver injury (possible Hy's Law), must be reported as an SAE (excluding studies of hepatic impairment or cirrhosis).

~~D. Local urine testing will be standard for the protocol unless serum testing is required by local regulation or IRB/IEC.~~

Section 13.4

The participant must be informed that his/her medical records may be examined by Clinical Quality Assurance auditors or other authorized personnel appointed by the Sponsor **and anTBiotic consortium members**, by appropriate IRB/IEC members, and by inspectors from regulatory authorities.

Section 13.6

~~**Pregnancy Testing**~~

~~Pregnancy testing will be performed whenever a menstrual cycle is missed or when pregnancy is otherwise suspected until the follow-up visit.~~

~~**Female Participants who become pregnant**~~

- ~~Investigator will collect pregnancy information on any female participant, who becomes pregnant while participating in this study.~~

- ~~Information will be recorded on the appropriate form and submitted to GSK within 24 hours of learning of a participant's pregnancy.~~
- ~~Participant will be followed to determine the outcome of the pregnancy. The Investigator will collect follow up information on participant and neonate, which will be forwarded to GSK Generally, follow-up will not be required for longer than 6 to 8 weeks beyond the estimated delivery date.~~
- ~~Any termination of pregnancy will be reported, regardless of fetal status (presence or absence of anomalies) or indication for procedure.~~
- ~~While pregnancy itself is not considered to be an AE or SAE, any pregnancy complication or elective termination of a pregnancy will be reported as an AE or SAE.~~
- ~~A spontaneous abortion is always considered to be an SAE and will be reported as such.~~
- ~~Any SAE occurring as a result of a post-study pregnancy which is considered reasonably related to the study treatment by the Investigator, will be reported to GSK as described in Section 13.5. While the Investigator is not obligated to actively seek this information in former study participants, he or she may learn of an SAE through spontaneous reporting.~~

~~Any female participant who becomes pregnant while participating will be withdrawn from the study.~~

- Agree to use a male condom plus an additional method of contraception with a failure rate of <1% per year as described in Table 3 when having penile-vaginal intercourse with a woman of childbearing potential, **for a period of 90 days after the last dose of study medication.**

Section 13.7

Elevated liver enzymes, transient liver dysfunction, hepatotoxicity and hepatitis are listed as side-effects in the package inserts for Rifafour e-275 (see References), associated with multiple drug components. Therefore, participants allocated to Rifafour e-275 treatment who experience Liver Chemistry Stopping criteria are not mandated in the first instance to undergo those follow-up assessments (which are intended to investigate aetiology). The investigations may still be performed, if required in the judgement of the investigator.

Required Actions and Follow up Assessments	
**Actions**	**Follow Up Assessments**
• **Immediately** discontinue study treatment<br>• Report the event to GSK **within 24 hours**<br>• Complete the liver event CRF and complete an SAE data collection tool if the event also meets the criteria for an SAE[2]<br>• Perform liver chemistry event follow up assessments<br>• Monitor the participant until liver chemistries resolve, stabilize, or return to within baseline (see **MONITORING** below)<br>• **Do not restart/rechallenge** participant with study treatment unless allowed per protocol and GSK Medical Governance approval **is** granted (see below)<br>• If restart/rechallenge **not allowed per protocol or not granted**, permanently discontinue study treatment and continue participant in the study for any protocol specified follow up assessments<br><br>**MONITORING:**<br>**<u>For bilirubin or INR criteria:</u>**<br>• Repeat liver chemistries (include ALT, AST, alkaline phosphatase, bilirubin) and perform liver event follow up assessments within **24 hrs**<br>• Monitor participants twice weekly until liver chemistries resolve, stabilize or return to within baseline<br>• A specialist or hepatology consultation is recommended<br>**<u>For All other criteria:</u>**<br>• Repeat liver chemistries (include ALT, AST, alkaline phosphatase, bilirubin) and perform	*<u>For all study participants:</u>*<br>• Obtain INR and recheck with each liver chemistry assessment until the transaminases values show downward trend<br>• **Record the appearance or worsening of clinical symptoms of liver injury, or hypersensitivity, on the AE report form**<br>• **Record use of concomitant medications on the concomitant medications report form including acetaminophen, herbal remedies, other over the counter medications.**<br>• **Record alcohol use on the liver event alcohol intake case report form (CRF) page**<br><br>***<u>For participants on GSK303656 arm</u>***<br>• **Viral hepatitis serology**[4]<br>• **Obtain** blood sample for pharmacokinetic (PK) analysis as soon as possible, and certainly within 14 days after last dose [5]<br>• Serum creatine phosphokinase (CPK) and lactate dehydrogenase (LDH).<br>• Fractionate bilirubin, if total bilirubin≥2xULN<br>• Obtain complete blood count with differential to assess eosinophilia<br><br>**<u>For bilirubin or INR criteria:</u>**<br>***<u>For participants on GSK303656 arm only</u>***

liver event follow up assessments within **24-72 hrs**<br>• Monitor participants weekly until liver chemistries resolve, stabilize or return to within baseline	• **Auto-immune** screen: Anti-nuclear antibody, anti-smooth muscle antibody, Type 1 anti-liver kidney microsomal antibodies, and quantitative total immunoglobulin G (IgG) or gamma globulins.<br>• Serum **paracetamol**/acetaminophen adduct high performance liquid chromatography (HPLC) assay (quantifies potential acetaminophen contribution to liver injury in participants with definite or likely acetaminophen use in the preceding week [James 2009]). **NOTE: not required in China**<br>• Liver imaging (ultrasound, magnetic resonance, or computerised tomography) and /or liver biopsy to evaluate liver disease; complete Liver Imaging and/or Liver Biopsy CRF pages.

Liver chemistry criteria for a Liver Monitoring Event and follow-up actions:

These Increased Monitoring Criteria apply to all participants who experience a Liver Event, regardless of which treatment the participant has been randomized to.

Liver Chemistry Increased Monitoring Criteria – Liver Monitoring Event	
**Criteria**	**Actions**
**ALT ≥3×ULN and <5×ULN and bilirubin <2×ULN, without symptoms believed to be related to liver injury or hypersensitivity, and who can be monitored weekly for 4 weeks.**	• **Notify the GSK medical monitor within 24 hours of learning of the abnormality by email or phone to discuss participant safety.**<br>• **Participant can continue study intervention.**<br>• **Participant must return weekly for repeat liver chemistries (ALT, AST, ALP, bilirubin) until they resolve, stabilise or return to within baseline.**

	• **If at any time participant meets the liver chemistry stopping criteria, proceed as described above**<br>• **If, after 4 weeks of monitoring, ALT <3×ULN and bilirubin <2×ULN, monitor participant twice monthly until liver chemistries normalize or return to within baseline**

The site will complete the liver event eCRF (Monitoring Event) in InForm and should also consider whether the AE reporting guidelines apply.

References

James LP, Letzig L, Simpson PM, Capparelli E, Roberts DW, Hinson JA, Davern TJ, Lee WM. Pharmacokinetics of Acetaminophen-Adduct in Adults with Acetaminophen Overdose and Acute Liver Failure. Drug Metab Dispos 2009; 37:1779-1784.

Rifafour e-275 tablet electronic package inserts. Accessed 20 Feb 2019.: http://home.intekom.com/pharm/hmr/rifaf275.html

END OF CHANGES

Amendment 2 24-SEP-2018

The primary motivation for this amendment was to update the eligibility criteria to impose a minimum renal function (as defined by creatinine clearance) in light of the first-in-human data that showed the main route of clearance for GSK3036656 is through the kidneys. As a result of the long half-life of GSK3036656, updated dosing strategy has also been implemented incorporating the use of loading doses.

Further changes made to the protocol are detailed in the table below.

Section #	Description of Change	Brief Rationale
Whole document	Minor updates including typographical corrections	Inconsistencies between different parts of the document have been resolved and clarifications inserted where required.
2	Removal of QTc endpoint	The intent of including Holter monitoring assessments in the EBA study was to collect data similar to that obtained in a thorough QT (TQT) study. These data would be used for an exposure-response analysis. However, since a positive control (moxifloxacin) will not be used in the EBA study (as required in a TQT study) and the EBA study is unlikely to achieve maximum concentrations of GSK3036656 that are in the range of 4- to 10-times the therapeutic dose (as required for 1 arm in a TQT study), the decision was made to remove Holter monitoring assessments from the EBA study.
3.3.1	Depigmentation	Depigmentation was seen in some animals in a 4-month GLP toxicology study. The risk-benefit assessment has been updated. Patients with vitiligo will be excluded from the study to avoid confounding in baseline and follow-up skin assessments.
3.3.1	Benefit-risk table - Clinical subsections updated with data from finalised 201040 CSR	Finalised data from the First in Human clinical trial are now available and have been incorporated.
2 & 3.3.1	Cardiac BNP assessment removed	Cardiac BNP is an acute phase reactant and has been reported to be elevated in a wide range of inflammatory conditions. Cardiac BNP monitoring is therefore considered to be of limited value in the context of patients with tuberculosis.

Section #	Description of Change	Brief Rationale
5.6	Loading doses	The long half-life of GSK3036656 means that 6 days are needed to achieve steady state concentrations in blood. The possibility of using loading doses has been included so that steady state may be reached more quickly. A rationale for how doses will be chosen has been included.
6.2	Removal of exclusion for patients with previous TB.	This criterion is obsolete. Until recently it was useful to exclude these patients based on the probability of rifampicin resistance TB in these subjects. Sputum smears have recently been replaced with GeneXpert MTB/RIF in the public health system and now provides reliable information about rifampicin resistance.
6.1.8	Renal clearance threshold	The main route of clearance for GSK3036656 is renal. There is therefore a risk that participants with low creatinine clearance will accumulate GSK3036656 and these patients have therefore been excluded.
8.1.3	Haematology stopping criteria	Black Africans have a wider normal range for neutrophil counts. The threshold for stopping IP is therefore revised from 1000/mm^3 to 500/mm^3.
9.3	Overdose window	The IP dosing has been permitted within a 2-hour window (24 hours±1 hour). The definition of an overdose has been updated to reflect this change.
10.3.4 & 11	Efficacy data analysis	There is uncertainty in how the efficacy of GSK3036656 will translate from animal models to the clinic. An efficacy data analysis following the cohort which receives the anticipated highest dose, will permit the sponsor to optimise dosing for subsequent cohorts as well as to aid internal decision making.<br>The dose escalation committee (DEC) has been empowered to assess and act on this data.

Section #	Description of Change	Brief Rationale
Appendix 6	Contraception guidance	The previous guidance was unclear and included durations in both weeks and days. This has now been clarified

CHANGES

**Period**	**Screening**			**Dosing**						**Follow-up**	
**Visit**	**1**	**2**	**3**	**4**	**5 to 10**	**11**	**12 to 16**	**17**	**18**	**Early Withdrawal**	**19**
**Day**	**(-9 to -3)** [A]	**-2**[B]	**-1**	**1**	**2 to 7**[C]	**8**	**9 to 13**[C]	**14**	**15**		**28 (± 7)**
Documentation of Positive GeneXpert and/or TB smear (TB clinic/site of initial diagnosis)	X										
Written Informed Consent	X										
Demography (including smoking history)	X										
Medical & Treatment History	X										
Inclusion/Exclusion/Eligibility Assessment	**X**										
Chest X-ray	**X**										
Physical Examination[D]	X			X	X	X	X	X	X	X	X
Vital Signs [E]	X	X	X	X	X	X	X	X	X	X	X
12-Lead ECG	X							**X**			X
Randomisation[M]		**X**									
Echocardiogram (may be conducted at any of the screening visits)	X									X[S]	X[S]
Blood PK[F]								X	X	X	
Spot Sputum (confirm TB & adequate bacterial load) [G]	X										
Rifampicin Resistance Test (rapid)[G] **(microscopy and GeneXpert tests may be repeated on an overnight sputum sample if needed)**	X										
Haematology, Clinical Chemistry, Urinalysis [H]	X			X	X	X		X		X	X
Cardiac safety biomarkers[T]	X			X	X			X			
Urine Drug Screen[I]	X	X[I]									
Urine Pregnancy Test[R]		X						X	X		
HIV Test (and CD4 Count if HIV confirmed)	X										

Period	Screening			Dosing						Follow-up	
Visit	1	2	3	4	5 to 10	11	12 to 16	17	18	Early Withdrawal	19
Day	(-9 to -3) [A]	-2[B]	-1	1	2 to 7[C]	8	9 to 13[C]	14	15		28 (± 7)
CCI [redacted]				X	X			X		X	
[redacted]				X	X			X		X	
[redacted]		X						X			
Hospital Admission	X[B]	X[B]									
Overnight Sputum[L]	X	X	X	X	X	X	X	X			
IP Administration and Compliance Check[N]				X	X	X	X	X			
Mycobacteriology Assessments [O]		X	X					X			
Hospital Discharge									X	X	
Concomitant Medication		X	X	X	X	X	X	X	X	X	X
Adverse Events[P]				X	X	X	X	X	X	X	X
Point of Care Blood Glucose or HbA1c[Q]		X						X	X		

A. The Visit 1 (day -9 to -3) time period will be up to a maximum of 7 days but will be kept as short as possible.

B. Participants can proceed with the Visit 2 (day -2) assessments as soon as their Visit 1 (day -9 to -3) assessments have been completed i.e. Visits 1 and 2 may occur on the same day as long as the screening results are available in time for randomisation. Participants may be hospitalised during the entire pre-treatment period if the Investigator considers it advisable for reasons of safety or compliance.

C. All events listed as occurring on Visit 5 (day 2) to Visit 10 (day 7) and Visit 12 (day 9) to Visit 16 (day 13) will be conducted each day during these visits.

D. Height (m) will only be collected once at Visit 1 (day -9 to -3). A full physical examination will be performed at Visit 1 (day -9 to -3), with symptom-directed physical examinations at Visit 17 (day 14), Visit 19 (day 28) and Early Withdrawal Visit (EWV). Symptom directed physical examinations may be conducted as required during the study (visits 4 to 16). **Requirements for the physical examination are specified in the SRM.**

E. Systolic blood pressure (SBP) and diastolic blood pressure (DBP) (mmHg), heart rate (beats per minute [bpm]), body temperature (°C; oral) and weight (kg). To be performed within 4 hours prior to the first daily dosing on Visit 4 (day 1) through to Visit 17 (day 14) and within 2 hours before the time dosing would have occurred on Visit 18 (day 15). On days where the following assessments are done the order should be: vital signs before blood draws for PK or safety assessments.

F. PKs will be performed as noted in the PK table. At EWV a PK sample will be taken pre-dose of the time of the planned first daily dosing. If this is not possible, a spontaneous PK sample will be taken.

G. Sputum smear microscopy and molecular rapid test for confirmation of Mycobacterium tuberculosis and rifampicin susceptibility (GeneXpert). **If required by the investigator**, the first spot sputum **or overnight sputum** does not show unequivocal results, the tests may be repeated on freshly collected spot sputum or overnight sputum and that result used **instead**.

H. Laboratory assessments: haematology (haemoglobin, haematocrit, red blood cell count, white blood cell count with differential, platelet count). Clinical Chemistry (albumin, urea, creatinine, direct, indirect and total bilirubin, total protein, alkaline phosphatase, aspartate aminotransferase (AST), alanine aminotransferase (ALT), gamma-glutamyl transferase (GGT), lactic dehydrogenase (LDH), sodium, potassium, calcium (corrected for albumin), chloride, random/fasting glucose). Urinalysis (pH, specific gravity, protein, glucose, micro-albumin, ketones, bilirubin, creatinine, nitrite, sodium, urobilinogen, blood, leukocytes, microscopy). Coagulation test (INR, PT, APTT). To be performed on Visit 4 (day 1), Visit 6 or 7 (day 3 or 4), Visit 11 (day 8) and within 2 hours before the time dosing would have occurred on Visit 18 (day 15).

I. Urine drug screen: cocaine, amphetamines and opiates. Urine drug screen can be repeated at Visit 2 (day -2) for participants who were not hospitalized at Visit 1 (day -9 to -3).

J. CCI [redacted]

K.

L. Overnight sputum sampling may start at Screening visit (day -9 to -3) or on Visit 2 (day –2) and will continue daily until Visit 7 (day 4). Additional sampling will be conducted at visit 9 (day 6), Visit 11 (day 8), Visit 13 (day 10), Visit 15 (day 12) and Visit 17 (day 14). Sputum sampling will stop on the morning of Visit 18 (day 15). Sputum collection will start in the afternoons at 15:00 hours (±1 hour) and continue for 16 hours overnight. The 16-hour sputum sampling for each of the sampling days must be finished prior to the administration of the next day's IP.

Overnight sputum collection may be collected **as many times as required over the screening period until an eligible sample is obtained**. Of the pre-treatment period samples collected, only the Visit 2 (day-2) and Visit 3(day -1) overnight sputum samples will be used for the efficacy endpoint tests.

The day sputum collection starts reflects the day to which that sample applies. e.g. a sample whose collection starts on day 1 and ends on day 2, is designated as the day 1 overnight sputum sample (and results).

M. Randomization may occur once all the screening results are available and the Investigator has determined that the participant is eligible for the trial.

N. After the first dose of IP, subsequent doses of IP will be administered 23 to 25 hours after the previous dose.

O. Minimum inhibitory concentration (MIC) and speciation of the infecting organism by polymerase chain reaction (PCR) will be estimated from a culture collected on a pre-treatment day (day-1 or day-2) and from the last available culture on a treatment day (day 14 or earlier, but not earlier than day 8). Drug susceptibility testing of *M. tuberculosis* for sensitivity to rifampicin with a molecular method will be tested at Visit 1. Susceptibility to INH, Rif, EMB and PZA will be tested on a culture grown from a baseline sample (day -2 or day -1).

P. Adverse events (AE) will be collected by the Investigator from the time a participant receives his/her first dose of IP through to the Visit 19 (day 28) Follow up Visit.

Q. CCI [redacted]

R. The urine pregnancy test at **Day -2 or Day -1 and Day** 14 must precede the CCI [redacted] and does not have to be repeated on the other days.

S. Only to be performed on participants receiving the anticipated highest dose or on participants with changes in clinical status warranting a follow-up echo in the opinion of the investigator. The dose escalation committee may decide to perform this assessment in additional cohorts within this study.

T. Samples will be assayed for: Troponin I. The first two samples at least 24 hours apart, one during the screening phase and one prior to the first dose of study treatment. Day 14 sample window is ±24 hours. Third sample to be taken on Day 3.

Section 3.3.1 Risk Assessment

Potential Risk of Clinical Significance	Summary of Data/Rationale for Risk	Mitigation Strategy
**Investigational Product (IP) GSK3036656**		
Reduction in red blood cell count	**Non-Clinical**<br><br>In a 4-week rat study (doses 3, 10 & 30 mg/kg/d) - dose-dependent decline in reticulocytes (by up to 52% in males and 62% in females) at day 7 followed by normal levels by day 14 and increased levels by day 29/30 compared to controls. Hgb levels decreased by 10-13% compared to controls on day 14 and day 29/30.<br><br>In a 4 week dog study (doses 5, 10 & 20 mg/kg/d) - mild dose-dependent decrease in Hgb on day 14 (by about 10% compared to controls) with recovery by week 4. Reticulocyte counts unaffected. Dogs not bled on day 7.<br><br>No histopathological changes in bone marrow or other relevant histopathology in the 4-week studies.<br><br>**Clinical**<br><br>**In the 201040 study, a possible trend of decreased haemoglobin from baseline to follow-up was noted in aggregate haematology data.**	Standard safety haematology and clinical chemistry assessments will be performed and both trends and changes outside normal range will be monitored as part of laboratory safety assessments.<br><br>Participants whose haemoglobin drops below pre-specified limits (<8.0 g/dL) will be withdrawn from the study (Section 8.1.3).<br><br>While women of non-childbearing potential are permitted in this study, women who are susceptible to heavy periods or heavy vaginal bleeding or spotting will be excluded in order to minimize blood loss and avoid confounding effects on the interpretation of haematology parameters.

Potential Risk of Clinical Significance	Summary of Data/Rationale for Risk	Mitigation Strategy
	**The mean change from baseline to follow-up was -4.9 g/L in the 15 mg repeat dose group, compared to -1.5 g/L in the placebo group. None of the values hit potential clinical importance (PCI) criteria, and declines were not clinically relevant.**<br><br>**No clinically significant trends in reticulocyte values were observed, and there were no values of potential clinical importance reported for reticulocytes.**	
**GSK3036656 is cleared through the kidneys**	**The main route of clearance for GSK3036656 is renal and there is therefore a risk that participants with low creatinine clearance will accumulate GSK3036656 and exceed predefined exposure limits.**	**Participants with a creatinine clearance less than 75 mL/min have been excluded.**
Reduction in white blood cell count	**Non-Clinical**<br><br>In the 4-week rat study, decrease of up to 37% in total leucocyte count on day 29/30 compared to controls.<br><br>In the 4-week dog study, no reported effects on white blood cells.	White blood cell count will be monitored as part of laboratory safety assessments. Any clinically significant changes will be followed up until levels are not clinically significant.

Potential Risk of Clinical Significance	Summary of Data/Rationale for Risk	Mitigation Strategy
	**Clinical**<br><br>**No clinically significant trends in leukocyte or neutrophil count values were observed. Two subjects showed PCI values. One subject had low neutrophil count (normal range 1.57-6.81 × 10^9/L) from screening (1.38) until follow-up (1.41), and also showed low leukocyte count (normal range 3.3-9.8 × 10^9/L) on Day 4 (2.5), Day 10 (2.6) and Day 14 (2.9) and was within normal range at follow-up. Given the low screening values, this observation was considered likely related to the subject's Black or African American race. A second subject had a single low neutrophil count at follow-up (1.48).**	Participants developing neutrophil counts <500/mm^3 (1.0x10^9/L) will be withdrawn from the study (Section 8.1.3).
Heart valvular and vascular pathology	**Non-Clinical**<br><br>In a 7-day dog dose range finding (DRF) study – minimal focal subendocardial hemorrhage on the atrial surface of the left atrioventricular valve observed in both animals at the top dose of 60 mg/kg/d.	This pathology is not monitorable in humans.<br><br>To avoid risk to humans, dosing will be limited to doses at which individual exposures [AUC (0-24)] do not exceed 4.9 µg.h/ml – 1/30 of the dog NOAEL exposure (from the 4-week GLP study) at which no heart pathology was seen.

Potential Risk of Clinical Significance	Summary of Data/Rationale for Risk	Mitigation Strategy
	In a 10-day dog investigative study at 65 mg/kg/d – vascular lesions (including minimal focal necrosis and inflammatory infiltrate) in 3 out of 8 dogs.<br><br>In the 4-week dog GLP study - no heart or valve changes were observed up to the top dose of 20 mg/kg/day and there was no increase in inflammatory markers.<br><br>Valvular or vascular pathology were not observed in rats. This pathological change is not commonly seen in animals, and its mechanism and significance to humans are not clear. A correlation between the presence of hemodynamic changes and the presence of this pathology is suspected, but currently unproven.<br><br>**Clinical**<br><br>Echocardiograms were performed at screening and follow up in **the 201040 FTIH study in** order to exclude participants with pre-existing valve or other cardiac abnormalities from the study, and to detect the presence of abnormalities after completion of the study. No abnormalities were detected.	In addition, although the pathology is not monitorable in humans, echocardiograms will be performed for all participants at screening and, for subjects receiving the highest dose of GSK3036656 or whose clinical status warrants it, also at follow-up (or early withdrawal). Cardiac troponin will be measured.
Hemodynamic changes	**Non-Clinical**	

Potential Risk of Clinical Significance	Summary of Data/Rationale for Risk	Mitigation Strategy
	Hemodynamic changes in several studies at ≥100 mg/kg in rats and ≥20 mg/kg in dogs - increased heart rate (rats and dogs), increased (rats) or decreased (dogs) blood pressure.<br><br>**Clinical**<br><br>Data from the 201040 FTIH study **showed** no significant changes in vital signs assessments during the study, and no clinically significant changes in ECG or telemetry assessments including QTc and PR intervals.	Vital signs monitoring will be implemented in this study.
Phototoxicity	**Non-Clinical**<br><br>As GSK3036656 absorbs light in the UVB wavelength range (>290 nm), there is a potential for phototoxicity in clinical populations.<br><br>**Clinical**<br><br>There were no adverse events reported **in the 201040 FTIH study** which would be considered consistent with phototoxicity.	Participants with a history of photosensitivity will be excluded from participating in the study (Exclusion Criteria, Section 6.1).<br><br>All study participants will be instructed to minimise exposure to sunlight and other sources of ultraviolet radiation (Section 6.3.1)

CCI

[redacted]

Potential Risk of Clinical Significance	Summary of Data/Rationale for Risk	Mitigation Strategy
		CCI [redacted]
Development of bacterial resistance to GSK3036656.	Administration of GSK3036656 as a monotherapy could potentially lead to the development of resistance to the compound in *M. tuberculosis* during the course of the study.	The risk of development of resistance in the EBA monotherapy study is considered to be low based on in-vitro data with GSK3036656 and historical experience with EBA studies in general. Susceptibility testing on samples of *M. tuberculosis* from participants will be conducted and assessed as part of the study to monitor for the development of resistance to GSK3036656. In-vitro data indicates that resistance to GSK3036656 does not confer any clinically significant cross resistance to SoC antitubercular treatment
Skin depigmentation	**Non-Clinical**<br><br>In a 4-month dog toxicology study (doses 0, 6, 20 & 45 mg/kd/day): loss of pigmentation on the eyelids,	The duration of dosing in this study (14 days) is shorter than that used in the dog toxicology study and the maximum exposure (AUC_{0-24}) has

Potential Risk of Clinical Significance	Summary of Data/Rationale for Risk	Mitigation Strategy
	nose or muzzle of all animals at 45 mg/kg/day group (week 17 $AUC_{0–t}$ mean 405 µg.h/mL) and most animals in the 20 mg/kg/day group (week 17 $AUC_{0–t}$ 176 µg.h/mL). PNL-2 (a melanocyte marker) immunohistochemistry staining demonstrated moderate cytoplasmic positivity in a population of cells in the basal region of the epidermis (interpreted to be melanocytes). The decreased pigmentation was therefore not associated with a loss of melanocytes in affected animals on immunohistochemistry. Depigmentation was not observed in the 0 or 6 mg/kg/day group (59.4 ug/h/mL). Depigmentation was not observed in the two previous 4 week dog toxicology studies up to 45 mg/kg/day (week 4 $AUC_{0-t.}$ 388 ug.h/mL)<br><br>**Clinical**<br><br>No adverse events were reported in the 201040 FTIH study that would be considered consistent with depigmentation.	been capped at 4.9 ug.h/mL (greater than 10-fold margin to the 6 mg/kg/day dose).<br><br>The physical examination of study participants will include visual examination of the skin.<br><br>Participants with vitiligo will be excluded from the study to avoid confounding skin assessment at baseline and follow-up physical examinations.

Section 5.6 Dose Justification

A loading dose of GSK3036656 may be administered on the first day of study treatment administration in each cohort. The long half-life of GSK3036656 (45 hours) means that subjects will take up to 10 days to reach steady-state concentrations, which is not optimal for a 14-day treatment study. A loading dose will allow participants to reach steady-state concentrations in 2 to 3 days for a given maintenance dose regimen.

The probability rates for individual participants to exceed GSK3036656 exposure limits and achieve the efficacy target for proposed loading and maintenance dose regimens are shown in Table 1.

Table 1 Probability rates of individual participants for proposed loading and maintenance doses

Loading Dose (mg)[a]	Probability Day 1[c]	Maintenance Dose (mg)[b]	Probability Day 14[d]	Target Attainment Rate[e]
10	< 1%	2.5	< 1%	< 1%
15	< 1%	5	< 1%	< 1%
20	2.4%	10	7.9%	100%

[a] Day 1

[b] Day 2-14

[c] per trial probability of an individual participant exceeding the exposure limits [AUC_{0-24} = 4.9 ug.h/mL or Cmax = 0.443 ug/mL] on Day 1 (simulations based on a lower limit of 75 mL/min for creatinine clearance)

[d] per trial probability of an individual participant exceeding the exposure limits [AUC_{0-24} = 4.9 ug.h/mL or Cmax = 0.443 ug/mL] on Day 14 (simulations based on a lower limit of 75 mL/min for creatinine clearance)

[e] per trial probability of >90% of participants exceeding the target exposure for efficacy

The starting dose regimen for this study will be 15 mg loading dose on Day 1 and 5 mg maintenance dose on Day 2-14, based on the above information, with the option to investigate a lower dose in subsequent cohorts should the preliminary data indicate this is appropriate.

The loading dose (if applicable) and maintenance dose for each cohort will be chosen on the basis that it is not predicted to exceed the pre-specified safety cap, and the dose used will be decided by the Dose Escalation Committee (Section 11).

Section 6.1. Inclusion Criteria

8. A creatinine clearance ≥75 mL/min (Cockroft-Gault formula).

Section 6.2 Exclusion Criteria

~~3. A previous episode of treated TB less than 3 years ago.~~

Section 8.1.3 8.1.3. Haematology Stopping Criteria

- **A reduction in neutrophil count to below 500/mm^3 (0.5 × 10^9/L).**

Section 9.3 Treatment of Overdose

For this study, any total dose of GSK3036656 greater than the dose selected for that cohort (**taken within a 23-hour period**) will be considered an overdose.

Section 10.3.4 Efficacy Analyses

No formal interim analyses are planned for this study. However, safety, tolerability and pharmacokinetic data will be reviewed before each dose escalation. **In addition, the primary efficacy endpoint may also be analysed after the final visit of the last subject for the cohort which receives the anticipated highest dose**. See Section 11 for further details.

Section 11 Dose Escalation

After the initial dose of GSK3036656, the decision to proceed to each subsequent dose level will be made by the Dose Escalation Committee (DEC) based on safety, tolerability and preliminary GSK3036656 pharmacokinetic data from the prior cohort. **The DEC may also include in their deliberations, efficacy data, if that is available**. Minimum requirements for data are as follows: PK data in at least 10 subjects receiving GSK3036656 and selected safety data from all subjects in a cohort. A dose escalation committee charter will be produced that will contain the following details:

- Committee members
- Data review requirements
- Process for informing the ethics committee of any safety concerns if appropriate
- Facility for requesting additional pertinent information form the investigator

Dose escalation will be based on population PK modelling (if feasible)**.** ~~or on the assumption of dose- exposure proportionality (i.e., doubling the dose gives an approximate doubling of exposure). If prior PK results show less than proportional~~

~~increase in exposure with increasing dose, the prediction will be based on the assumption that the fold exposure increase to fold dose increase ratio will be the same with the current dose escalation as it was with the previous one. If prior PK results show more than proportional increase in exposure with dose, subsequent~~. **Dose escalations will not be higher than 3-fold.**

The population PK model will be a fit for purpose model. Candidate model selection will be primarily based on a significant reduction in the objective function value (≥ 3.32, $\chi 2$ <0.05) and improvement in the fits of diagnostic scatter plots. Inter-individual variability will be modelled using an exponential error model and residual error will be described by a proportional, proportional plus additive, or additive error model, depending on the data. Model performance will be evaluated using a visual predictive check. Simulations for dose recommendations will be conducted to determine the probability of an individual subject exceeding the defined exposure margins for AUC and Cmax.

Alternatively, if a population PK model is not feasible, dose escalation will be based on a dose-exposure model of the form:

log(AUC) = a + b*log(DOSE) + c*log(BWT/70)

log(CMAX) = d + e*log(DOSE) + f*log(BWT/70)

where a and d are AUC and Cmax values, respectively; c = -0.75 and f = -1.00 are set values for allometric scaling; and b and e are scaling factors for dose proportionality.

A proposed dose will be acceptable if the predicted probability of an individual exceeding pre-defined exposure limits (Section 5.6) on any day of the dosing period, as estimated by PK modeling, is no more than 10%.

The primary endpoint may be analysed for all available cohorts after the final visit of the last subject for the cohort which receives the anticipated highest dose (as determined by the DEC). The purpose of this efficacy analysis is to optimise dosing for subsequent cohorts (e.g. to help estimate the dose response curve) as well as to aid internal decision making.

The DEC is empowered to take the following decisions for subsequent cohorts:

- **To select a higher dose within the limits defined in section 5.6.**
- **To select a dose lower than, or the same as, a preceding dose.**
- **To stop dosing.**
- **To include additional pharmacokinetic assessments.**
- **To include additional timepoints for existing safety assessment.**

Section 13.6 Appendix 6

Contraception Guidance

Male participants

- Male participants with female partners of child-bearing potential are eligible to participate if they agree to ONE of the following:

    - Are abstinent from penile-vaginal intercourse as their usual and preferred lifestyle (abstinent on a long term and persistent basis) and agree to remain abstinent **for a period of 90 days after the last dose of study medication (one spermatogenesis cycle)**.
    - Agree to use a male condom plus an additional method of contraception with a failure rate of <1% per year as described in Table 3 when having penile-vaginal intercourse with a woman of childbearing potential.

- Men with a pregnant or breastfeeding partner must agree to remain abstinent from penile-vaginal intercourse or use a male condom during each episode of penile penetration during the treatment period and for 6 **days after (five terminal half-lives).**
- In addition, male participants must refrain from donating sperm during the treatment period and for **90 days** after **(one spermatogenesis cycle)**.

Amendment 1 (20-Feb-2018)

Overall Rationale for the Amendment:

[redacted] Minor typographical corrections throughout the document have also been applied.

Section #	Description of Change	Brief Rationale
3.3.1	[redacted]<br>3. Correction of typographical error regarding menstruation	Clarity and consistency of the document and implementation of additional safety measures for participants
5.1	[redacted]	Design clarity
6.1 & 6.2	1. [redacted] must be 25 years of age or older.<br>2. Wording clarification for inclusion criteria #5<br>3. Wording clarification for inclusion criteria #6<br>4. Wording clarification for exclusion criteria #11<br>[redacted]<br>6. Changed text to match 3.3.1 regarding menstruation	Design clarity & clarity and consistency of the document.
9.1	Clarification wording in section header and definition of timepoint nomenclature	Clarity and consistency of the document
9.6& 13.8	[redacted]	
All document	Minor typographical corrections	Clarity of document

Section 3.3.1 Risk Assessment

CHANGES

CCI

[redacted]

	–The level of risk may therefore be assumed to broadly fall within the ICRP 62 intermediate Category IIb, where the benefit should be more directly aimed at the cure or prevention of disease.	[redacted]<br>[redacted] Clinically justified (therapeutic or diagnostic) exposures are not included in the exposure calculation. Participants are asked about any occupational exposure or previous participation in research studies at screening so that dose estimates can be obtained where necessary.<br><br>The study will exclude women of childbearing potential.<br><br>[redacted]

		[redacted]

Section 5.1 Overall Design

CHANGES

In addition, the minimum age for inclusion for participants recruited to these 2 cohorts will be raised from ≥18 to ≥25 years old.

Section 6.1 Inclusion criteria

CHANGES

Age

1. Participant must be 18 to 65 years of age inclusive, at the time of signing the informed consent.

2. [redacted]

Section 9.1 Microbiological Efficacy/Activity Assessments

CHANGES

Drug activity with CFU or TTP over different treatment periods such as baseline - **Day2**, and **Day**2-14, for cross-comparison with other published data. Data will be displayed graphically, together with modelled estimates.

For the purposes of the microbiological endpoints assessments, the pre-treatment baseline will be defined as either Day -2 and/or Day -1.

Mycobacterial Characterisation:

The identifying organism will be confirmed as Mycobacterium tuberculosis at least once by polymerase chain reaction (PCR) at screening (GeneXpert or Line Probe) and a sample collected before treatment **will be submitted for culture confirmation**.

Section 9.6. Potential Surrogate Disease/Biomarkers

CHANGES

CCI

[redacted]

CCI
[redacted]
