# Supplementary material for: A first-in-class leucyl-tRNA synthetase inhibitor, ganfeborole, for rifampicin-susceptible tuberculosis: a phase 2a open-label, randomized trial
Source: Nat Med. 2024 Feb 16;30(3):896–904. doi: 10.1038/s41591-024-02829-7 (PMC10957473; doi:10.1038/s41591-024-02829-7)
Supplement: Supplementary file 2 — Reporting Summary [file 41591_2024_2829_MOESM2_ESM.pdf]

nature portfolio

Corresponding author(s): David Barros-Aguirre

Last updated by author(s): Dec 19, 2023

Reporting Summary

Nature Portfolio wishes to improve the reproducibility of the work that we publish. This form provides structure for consistency and transparency in reporting. For further information on Nature Portfolio policies, see our Editorial Policies and the Editorial Policy Checklist.

Statistics

For all statistical analyses, confirm that the following items are present in the figure legend, table legend, main text, or Methods section.

n/a	Confirmed	
☐	☒	The exact sample size (*n*) for each experimental group/condition, given as a discrete number and unit of measurement
☐	☒	A statement on whether measurements were taken from distinct samples or whether the same sample was measured repeatedly
☐	☒	The statistical test(s) used AND whether they are one- or two-sided<br>*Only common tests should be described solely by name; describe more complex techniques in the Methods section.*
☐	☒	A description of all covariates tested
☐	☒	A description of any assumptions or corrections, such as tests of normality and adjustment for multiple comparisons
☐	☒	A full description of the statistical parameters including central tendency (e.g. means) or other basic estimates (e.g. regression coefficient) AND variation (e.g. standard deviation) or associated estimates of uncertainty (e.g. confidence intervals)
☒	☐	For null hypothesis testing, the test statistic (e.g. *F*, *t*, *r*) with confidence intervals, effect sizes, degrees of freedom and *P* value noted<br>*Give P values as exact values whenever suitable.*
☒	☐	For Bayesian analysis, information on the choice of priors and Markov chain Monte Carlo settings
☒	☐	For hierarchical and complex designs, identification of the appropriate level for tests and full reporting of outcomes
☒	☐	Estimates of effect sizes (e.g. Cohen's *d*, Pearson's *r*), indicating how they were calculated

Our web collection on statistics for biologists contains articles on many of the points above.

Software and code

Policy information about availability of computer code

Data collection	Post-hoc analysis: For the analysis of the RNA data, the count matrix was first created. This formed the basis for the application of the CIBERSORT algorithm (https://cibersortx.stanford.edu/). Furthermore, an enrichment analysis was performed to distinguish between participants who showed improvement in PET/CT signal and those who showed deterioration.
Data analysis	Post-hoc analysis: The 50 genes with the strongest effect to discriminate between participants with improvement and deterioration were assigned using KEGG Pathway identifiers (https://www.genome.jp/kegg/pathway.html) to link them to their respective pathways

For manuscripts utilizing custom algorithms or software that are central to the research but not yet described in published literature, software must be made available to editors and reviewers. We strongly encourage code deposition in a community repository (e.g. GitHub). See the Nature Portfolio guidelines for submitting code & software for further information.

Data

Policy information about availability of data

All manuscripts must include a data availability statement. This statement should provide the following information, where applicable:

- Accession codes, unique identifiers, or web links for publicly available datasets
- A description of any restrictions on data availability
- For clinical datasets or third party data, please ensure that the statement adheres to our policy

Please refer to GSK weblink to access GSK's data sharing policies and as applicable seek anonymized subject level data via the link https://www.gsk-studyregister.com/en/

Research involving human participants, their data, or biological material

Policy information about studies with human participants or human data. See also policy information about sex, gender (identity/presentation), and sexual orientation and race, ethnicity and racism.

Reporting on sex and gender	All participants enrolled in this study were male due to preclinical teratogenicity data that became available after the phase 1 study.
Reporting on race, ethnicity, or other socially relevant groupings	All participants enrolled in this study were Black which, although not atypical for the TB population in South African clinics, may challenge the generalizability to other populations; thus, additional testing in other race groups is required.
Population characteristics	Covariate-relevant population characteristics including sex, age, and race are included in Table 1.
Recruitment	Participants were recruited at the TASK clinical research center in Cape Town, South Africa, according to the study eligibility criteria. Participants attended the clinic as they had TB and they were asked if they were interested in participating int his trial. The sponsor is unaware of any patient selection bias.
Ethics oversight	The study protocol was reviewed and approved by a national ethics committee or institutional review board, in accordance with the International Council for Harmonisation of Technical Requirements for Pharmaceuticals for Human Use guideline for Good Clinical Practice (GCP) and applicable country-specific requirements. The study was conducted in accordance with ethical principles that have their origins in the Declaration of Helsinki and the principles of GCP. Ethical approval was granted by Pharma-Ethics, South Africa. Regulatory approval was granted by the South African Health Products Regulatory Authority.

Note that full information on the approval of the study protocol must also be provided in the manuscript.

Field-specific reporting

Please select the one below that is the best fit for your research. If you are not sure, read the appropriate sections before making your selection.

☒ Life sciences ☐ Behavioural & social sciences ☐ Ecological, evolutionary & environmental sciences

For a reference copy of the document with all sections, see nature.com/documents/nr-reporting-summary-flat.pdf

Life sciences study design

All studies must disclose on these points even when the disclosure is negative.

Sample size	The original estimated enrollment totaled 100 participants. As no formal statistical hypothesis was tested, no formal sample size calculation was performed. However, simulations were conducted to determine the impact of the size of the study on the ability to detect a treatment effect based on the mean daily decline from baseline in log10CFU/mL sputum in participants treated with GSK3036656. Based on earlier EBA studies, a standard deviation (SD) of 0.2 was used for the change from baseline in log10CFU for the simulations. Assuming a true underlying change from baseline of −0.06 and a sample size of 15 participants per group, there was approximately 90% probability of the observed mean change being a negative value (i.e., a positive response indicated by a decline in bacterial load being observed). Due to a slower than anticipated rate of recruitment (during the COVID-19 pandemic), a review of the SD of daily change in log10CFU data from participants in the first two cohorts concluded that the observed SD was smaller than that used in the initial sample size simulation. Based on subsequent simulations, it was estimated that assuming a true underlying change from baseline of −0.06, a SD of 0.15, and a sample size of nine participants per group, there was approximately 90% chance of the observed mean change being a negative value; therefore, nine participants were selected for ongoing and future GSK3036656 cohorts.
Data exclusions	No data were excluded from the analysis.
Replication	Not replicated.
Randomization	Participants were randomized 3:1 per cohort to receive GSK3036656 or SOC for drug-susceptible TB. On or before Day 1, eligible participants were assigned a unique randomization number in ascending numerical order at the study site. The randomization number encoded the participant's assignment to GSK3036656 or SOC, according to the randomization schedule generated prior to the study.

Blinding	The study was open label, so neither the participants nor the primary investigators were blinded. However, all laboratory staff involved in analyzing and reporting the log10CFU counts and TTP endpoints results were blinded to treatment allocation.

Reporting for specific materials, systems and methods

We require information from authors about some types of materials, experimental systems and methods used in many studies. Here, indicate whether each material, system or method listed is relevant to your study. If you are not sure if a list item applies to your research, read the appropriate section before selecting a response.

Materials & experimental systems

n/a	Involved in the study
☒	☐ Antibodies
☒	☐ Eukaryotic cell lines
☒	☐ Palaeontology and archaeology
☒	☐ Animals and other organisms
☐	☒ Clinical data
☒	☐ Dual use research of concern
☒	☐ Plants

Methods

n/a	Involved in the study
☒	☐ ChIP-seq
☒	☐ Flow cytometry
☒	☐ MRI-based neuroimaging

Clinical data

Policy information about clinical studies
All manuscripts should comply with the ICMJE guidelines for publication of clinical research and a completed CONSORT checklist must be included with all submissions.

Clinical trial registration	NCT03557281
Study protocol	The study protocol has been submitted with this manuscript.
Data collection	This study was conducted from March 2019 to December 2021 at the TASK clinical research center in Cape Town, South Africa.
Outcomes	The primary objective of the study was to determine the EBA of GSK3036656 QD over 14 days of repeat dosing, as assessed by the rate of change in log10CFU/mL sputum from baseline to Day 14 (EBA CFU0–14).<br>The secondary EBA objectives were to determine the EBA of GSK3036656 QD over the first 2 days and the last 12 days. They were assessed by the change in log10CFU/mL sputum from baseline to Day 2 (EBA CFU0–2) and Day 2 to Day 14 (EBA CFU2–14), and by the rate of change in time to sputum culture positivity from baseline to Day 14 (EBA TTP0–14), baseline to Day 2 (EBA TTP0–2), and Day 2 to Day 14 (EBA TTP2–14).<br>Additional secondary objectives were to evaluate the safety and tolerability of GSK3036656 QD over 14 days, determined by AE reporting and monitoring of clinical laboratory values, vital signs, and electrocardiogram parameters. Furthermore, the aim of the study was to characterize the PK of GSK3036656 in participants following repeat QD dosing for 14 days. PK parameters of interest were derived using non-compartmental analysis including AUC, Cmax, and tmax.<br>Characterizing the radiological features of TB lesions by PET/CT scan to detect treatment-induced changes was an exploratory objective. Changes in lesion volume, total glycolytic activity, and impact on extent of disease as measured by lesion characteristics (consolidations, infiltrates, cavities) were determined. Furthermore, the changes in PET/CT signal were correlated with the RNA profiles of the participants at baseline and Day 14.
